# Chemical Composition and Antibacterial Activity of Essential Oils from Different Organs of *Pistacia falcata*

**DOI:** 10.3390/molecules31193487

**Published:** 2026-10-01

**Authors:** Ammar Bader, Bashaer Alsharif, Katia Scortecci, Guido Flamini, Fabio Boylan, Sinéad C. Corr

**Affiliations:** 1 Department of Pharmaceutical Sciences, Faculty of Pharmacy, Al-Ahliyya Amman University, Amman 19328, Jordan; 2 Department of Pharmaceutical Sciences, Faculty of Pharmacy, Umm Al-Qura University, Makkah 21955, Saudi Arabia; basharif@uqu.edu.sa; 3 Departamento de Biologia Celular e Genética, Universidade Federal do Rio Grande do Norte, Natal 59072-970, Brazil; katia.scortecci@ufrn.br; 4 Department of Pharmacy, University of Pisa, Via Bonanno 6, 56126 Pisa, Italy; guido.flamini@unipi.it; 5 School of Pharmacy and Pharmaceutical Sciences, Panoz Institute, and Trinity Biomedical Sciences Institute, Trinity College Dublin, Dublin 2, D02 PN40 Dublin, Ireland; fabio.boylan@tcd.ie; 6 School of Microbiology/APC Microbiome Ireland, University College Cork, T12 YT20 Cork, Ireland; scorr@ucc.ie

**Keywords:** *Pistacia falcata*, essential oils, GC–MS, monoterpenes, antibacterial activity, *Staphylococcus aureus*

## Abstract

*Pistacia falcata* Becc. ex Martelli (Anacardiaceae) is a medicinal species distributed across parts of the Middle East and Africa. This study characterized the essential oils from different organs of *P. falcata* and evaluated their antibacterial and antibiofilm activities. Essential oils from leaves, roots, inflorescences and fruiting inflorescences were obtained by hydrodistillation and analyzed by GC–MS. The essential oils were dominated by monoterpene hydrocarbons, mainly α-pinene (15.9–80.4%), myrcene (1.3–45.1%), (Z)-β-ocimene (0–14.3%), and α-phellandrene (0.40–19.7%). Bornyl acetate was the principal oxygenated monoterpene (up to 4.8%). Among sesquiterpene hydrocarbons, bicyclogermacrene (up to 2.4%) and germacrene D (up to 2.1%) were the most abundant, whereas oxygenated sesquiterpenes were represented mainly by (*E*)-nerolidol (0.1–7.1%). The diterpenes m-camphorene and p-camphorene were detected only in the leaves essential oil. Antibacterial activity was evaluated against *Staphylococcus aureus* NCTC 6571, *Escherichia coli* MG1655, and *Pseudomonas aeruginosa* PAO1. The inflorescence and fruiting-inflorescence oils showed the greatest activity against *S. aureus* (MIC and MBC = 5.3 mg/mL), whereas the leaf oil was less active (MIC and MBC = 10 mg/mL), and the root oil was inactive at the highest concentration tested (MIC > 20 mg/mL). No inhibition was detected against *E. coli* or *P. aeruginosa*, although changes in *P. aeruginosa* pigmentation were observed at 5.3–10 mg/mL. Growth-kinetics assays confirmed sustained inhibition of *S. aureus* by the inflorescence oil at 5.3 mg/mL over 24 h. In contrast, the oils did not significantly inhibit *P. aeruginosa* biofilm formation. These findings provide the first chemical profile of *P. falcata* essential oils and indicate activity varied among oils obtained from different organs, mainly against Gram-positive bacteria.

## 1. Introduction

The genus *Pistacia* has considerable medicinal, economic and nutritional importance [1]. Several *Pistacia* species have been used traditionally to treat a range of conditions, including microbial infections. Their antimicrobial activity has been attributed not only to essential oils [2] but also to other secondary metabolites, including monoterpenoid glycosides, flavonoids [3], phenolic derivatives [4], and tannins [5]. Essential oils from different *Pistacia* species have been tested against Gram-negative and Gram-positive bacteria, as well as fungi [6]. Antimicrobial activity has been assessed using agar disk diffusion, minimum inhibitory concentration (MIC) [6], minimum bactericidal concentration (MBC), membrane permeability and integrity assays [7] and antibiofilm models [8].

In Saudi Arabia, only two *Pistacia* species grow wild: *Pistacia falcata* Becc. ex Martelli and *P. khinjuk* Stocks [9]. *P. falcata* remains poorly investigated. In a previous study, the leaf extract was analyzed by HPLC–DAD–MS/MS and was shown to be rich in phenolic acids and glucuronidated flavonoids; the extract also exhibited acetylcholinesterase and monoamine oxidase inhibitory activity [10]. However, the composition of the essential oil of *P. falcata* has not yet been reported.

Antimicrobial resistance is a major global health problem, driven in part by diagnostic uncertainty and the misuse or overuse of antibiotics [11,12]. Biofilm formation is one of the mechanisms that contribute to reduced antimicrobial susceptibility [13]. Because biofilm-associated cells are more tolerant to conventional antibiotics, the identification of alternative or adjuvant antimicrobial strategies remains a priority. Essential oils are complex mixtures of terpenoids and other volatile compounds that may interfere with bacterial membranes, adhesion, quorum sensing and biofilm development. Several medicinal plants from the Middle Eastern flora have recently been examined for antimicrobial and antibiofilm activities [13,14,15]. In addition, essential oils from *Pistacia* spp., including *P. lentiscus*, *P. atlantica* and *P. vera*, have shown antibacterial and antibiofilm properties against a range of Gram-positive and Gram-negative pathogens, as well as *Candida* species [8,16]. To the best of our knowledge, however, no data are available on the chemical composition or antimicrobial and antibiofilm activities of *P. falcata* essential oils.

Therefore, this study aimed to characterize the chemical composition of essential oils obtained from different organs of *P. falcata* and to evaluate their antimicrobial and antibiofilm activities against selected bacterial strains.

## 2. Results

### 2.1. Essential Oil Analysis

GC–MS analysis of the essential oils obtained from leaves, roots, inflorescences, and fruiting inflorescences of *P. falcata* revealed the presence of 67 volatile constituents (Table 1). The oils were dominated by monoterpene hydrocarbons (74.6–94.5%), followed by oxygenated monoterpenes (1.7–8.7%), sesquiterpene hydrocarbons (0–7.0%), and oxygenated sesquiterpenes (0.1–7.2%). Diterpenes were detected only in the leaf essential oil (1.0%), whereas non-terpenoid compounds occurred in trace amounts (0–0.8%).

All organs contained α-pinene as a major constituent, but each oil showed a distinct compositional profile. The fruiting-inflorescence and root essential oils had the simplest profiles, with 23 listed constituents each. The fruiting-inflorescence oil was dominated by α-pinene (27.8%), α-phellandrene (19.7%), limonene (12.3%), myrcene (11.2%), camphene (6.2%), and β-pinene (5.2%), while sesquiterpenes were present only in minor amounts. The root essential oil was also rich in monoterpene hydrocarbons (90.7%) but was chemically distinct from the fruiting-inflorescence oil. It was dominated by α-pinene (80.4%) and β-pinene (4.0%). Oxygenated sesquiterpenes reached their highest percentage in this sample, mainly because of (E)-nerolidol (7.1%).

The inflorescence oil was the most chemically diverse sample, with 44 components identified and a more balanced distribution of terpene classes. Monoterpene hydrocarbons remained the predominant class (74.6%), but oxygenated monoterpenes (8.7%), sesquiterpene hydrocarbons (7.0%), and oxygenated sesquiterpenes (6.2%) were more abundant than in the other organs. The main constituents were α-pinene (28.3%), α-phellandrene (10.1%), limonene (9.5%), camphene (5.5%), p-cymene (4.9%), β-pinene (4.8%), and bornyl acetate (4.8%). Relevant amounts of sesquiterpenes included germacrene D (2.1%), bicyclogermacrene (2.4%), α-cadinol (2.5%), spathulenol (0.9%), and viridiflorol (0.9%).

The leaf essential oil showed a distinct profile characterized by a high proportion of monoterpene hydrocarbons (89.9%). Myrcene was the main constituent (45.1%), followed by α-pinene (15.9%), (Z)-β-ocimene (14.3%), (E)-β-ocimene (4.3%), α-phellandrene (3.8%), and limonene (3.5%). The leaf oil also contained sesquiterpene hydrocarbons (3.3%), including germacrene D (1.2%), and oxygenated sesquiterpenes (2.4%), including small percentages of viridiflorol and α-cadinol. It was the only sample in which diterpenes were detected (1.0%).

The monoterpene-rich profile observed here is consistent with previous reports for *Pistacia* species, in which α-pinene is frequently a predominant constituent (e.g., *P. lentiscus*, *P. atlantica*, *P. palaestina*, and *P. vera*) [17,18,19,20,21]. In the present samples, α-pinene was the major constituent in all organs except the leaves, where myrcene predominated. A similar pattern, with myrcene together with α- and β-pinene as major constituents, has been reported for *P. lentiscus* var. chia [22]. Myrcene also occurs in Chios mastic gum as cis-1,4-poly-β-myrcene [23].

**Figure 1:**
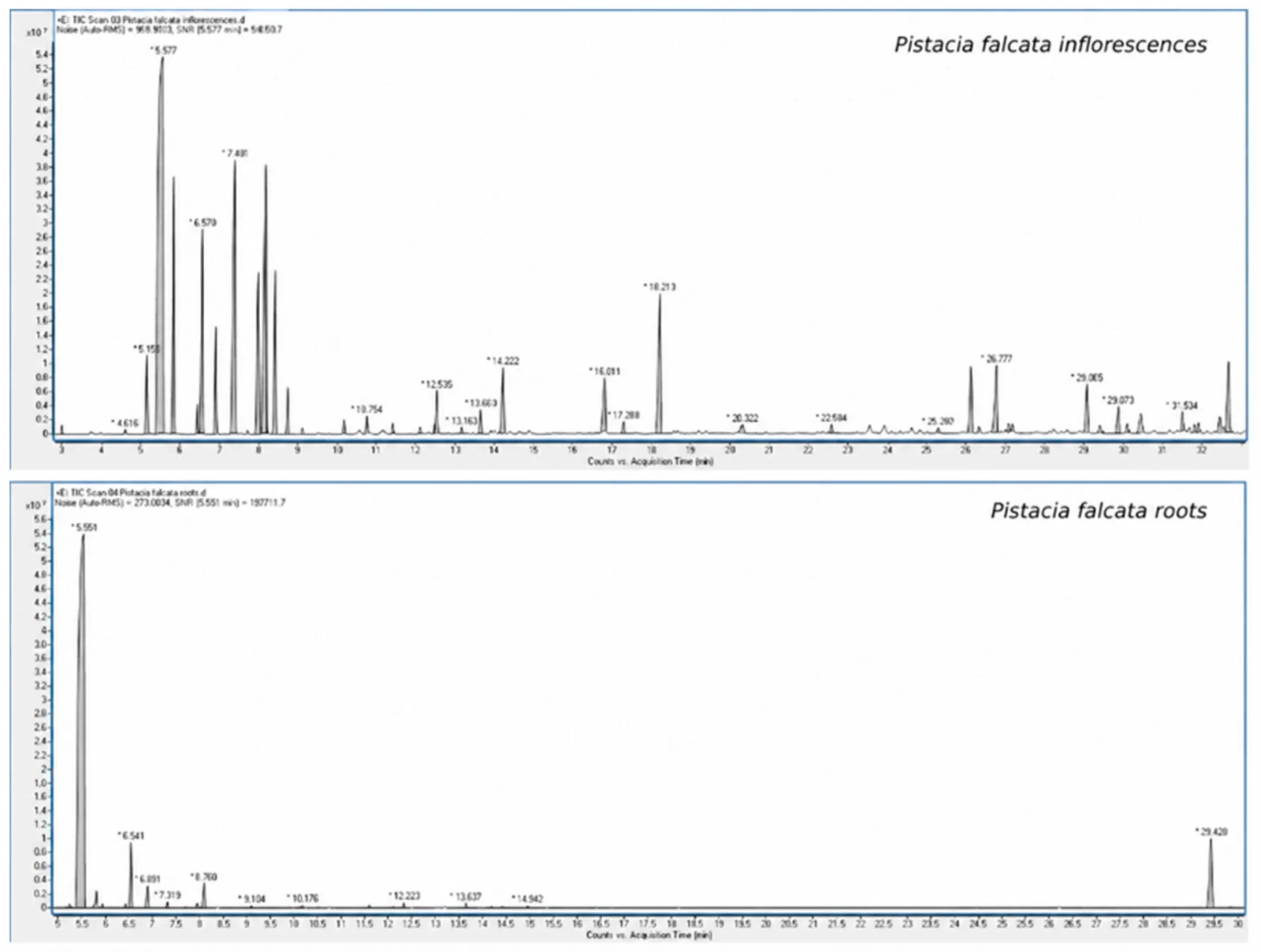

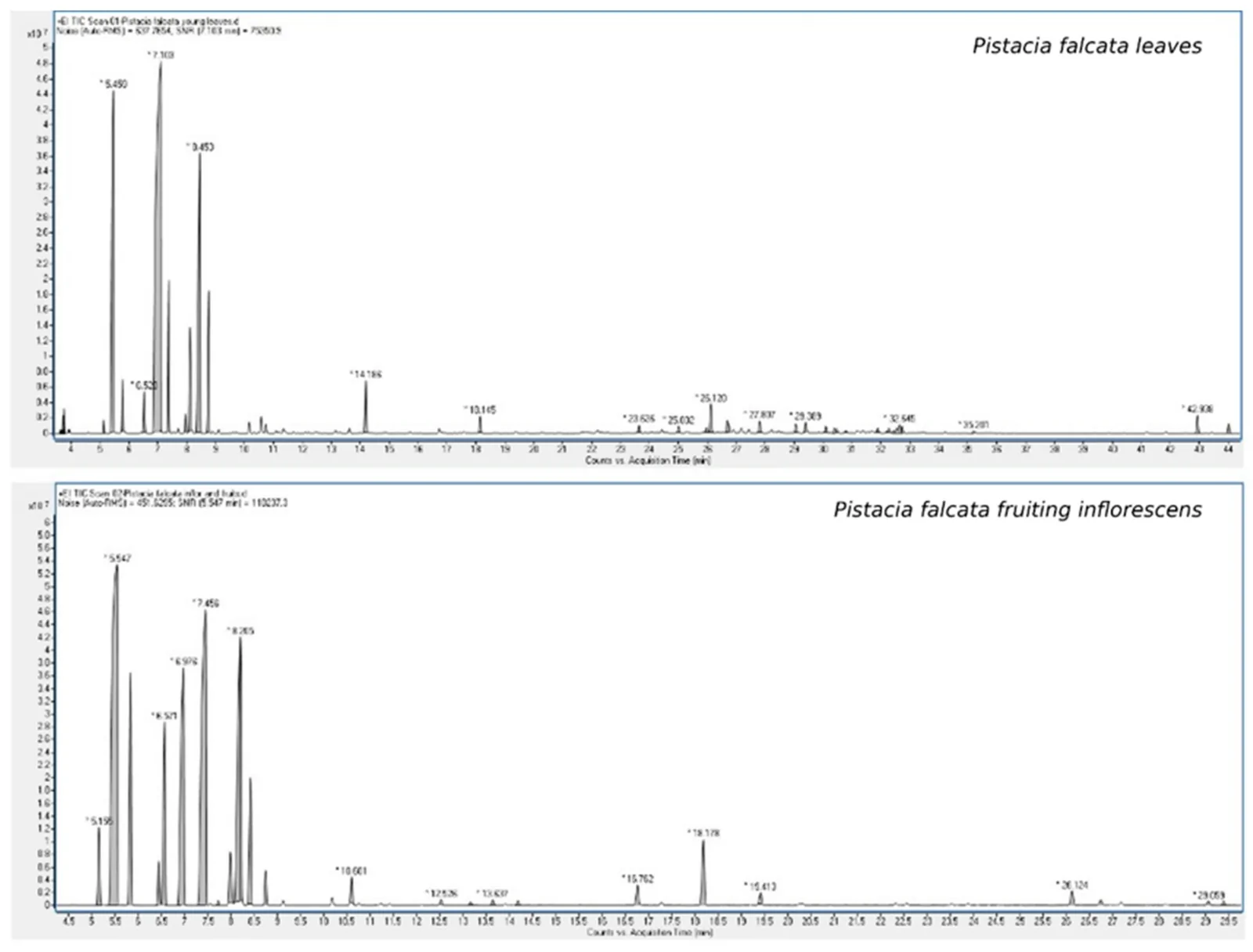
Total ion chromatograms (TICs) of the essential oils obtained from different organs of *P. falcata*.

Among the distinctive constituents of *P. falcata* were (E)-nerolidol, which reached 7.1% in the root oil, and the diterpene hydrocarbons m-camphorene and p-camphorene, which were detected only in the leaf oil (1.0% in total). Their occurrence is reported here as a compositional feature; no inference is made regarding the rarity of diterpenes within *Pistacia*, because their detection may also depend on the plant material and analytical conditions. The total ion chromatograms (TICs) of the essential oils is shown in Figure 1.

### 2.2. Antibacterial Activity and Growth Kinetics

#### 2.2.1. Antibacterial Activity (MIC and MBC)

The antibacterial activity of *P. falcata* essential oils was evaluated against *Staphylococcus aureus* NCTC 6571, *Escherichia coli* MG1655, and *Pseudomonas aeruginosa* PAO1. Activity was detected only against the Gram-positive strain *S. aureus.* No inhibitory effect was observed against *E. coli* or *P. aeruginosa* at the concentrations tested, although changes in *P. aeruginosa* pigmentation were noted at 5–10 mg/mL.

MIC and MBC values are summarized in Table 2. The inflorescence and fruiting inflorescence oils showed antibacterial activity, with MIC and MBC values of 5.3 mg/mL. The leaf essential oil was less active (MIC and MBC = 10 mg/mL), whereas the root essential oil was inactive at the highest concentration tested (MIC > 20 mg/mL). For all active oils, MBC values were identical to MIC values, suggesting a bactericidal effect under the conditions used.

Bacterial aggregation was observed in *S. aureus* cultures treated with essential oils from the inflorescences, fruiting inflorescences, and leaves at concentrations ranging from 5.3 to 20 mg/mL. No aggregation was observed with the root essential oil or in the control groups (medium only or medium with bacteria).

#### 2.2.2. Growth Kinetics Assay

**Figure 2:**
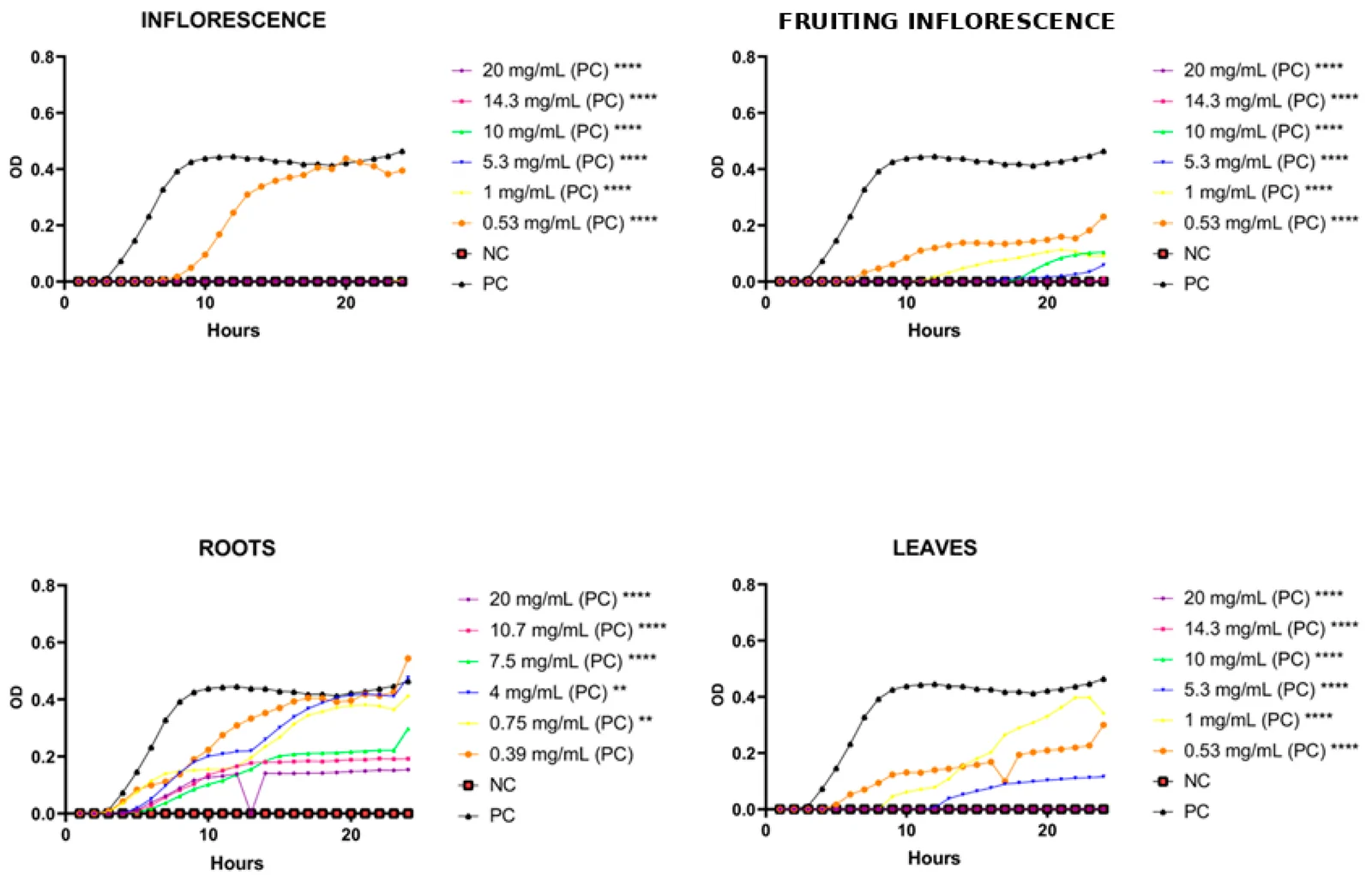
Growth kinetics of *P. falcata* essential oils against *Staphylococcus aureus*. Growth was monitored at 600 nm in a 96-well plate reader over 24 h. Data represent four independent experiments. Statistical analysis was performed using one-way ANOVA followed by Tukey’s test (** *p* < 0.01 and **** *p* < 0.0001).

Time-dependent effects of the essential oils on the growth of *S. aureus* were evaluated over 24 h (Figure 2).

The essential oil obtained from inflorescences produced a sustained inhibition of bacterial growth, particularly at concentrations of 5.3 mg/mL or higher, where growth remained suppressed throughout the 24 h period. At 1.0 mg/mL, a prolonged lag phase was followed by delayed growth, indicating a concentration-dependent effect. A similar profile was observed for the fruiting inflorescence oil, which maintained suppression at higher concentrations.

For the root essential oil (Figure 2, ROOTS), although the visual MIC was determined as >20.0 mg/mL, higher concentrations (10.7−0.0 mg/mL) consistently maintained ${\mathrm{OD}}_{600}$ values below those of the untreated growth control throughout the 24 h evaluation, indicating partial growth suppression and delayed kinetic progression.

The leaf essential oil showed intermediate activity. Sustained inhibition was observed at 10–20 mg/mL, whereas lower concentrations produced partial or delayed inhibition and a slower growth rate than the untreated control.

#### 2.2.3. Biofilm Formation Assay

**Figure 3:**
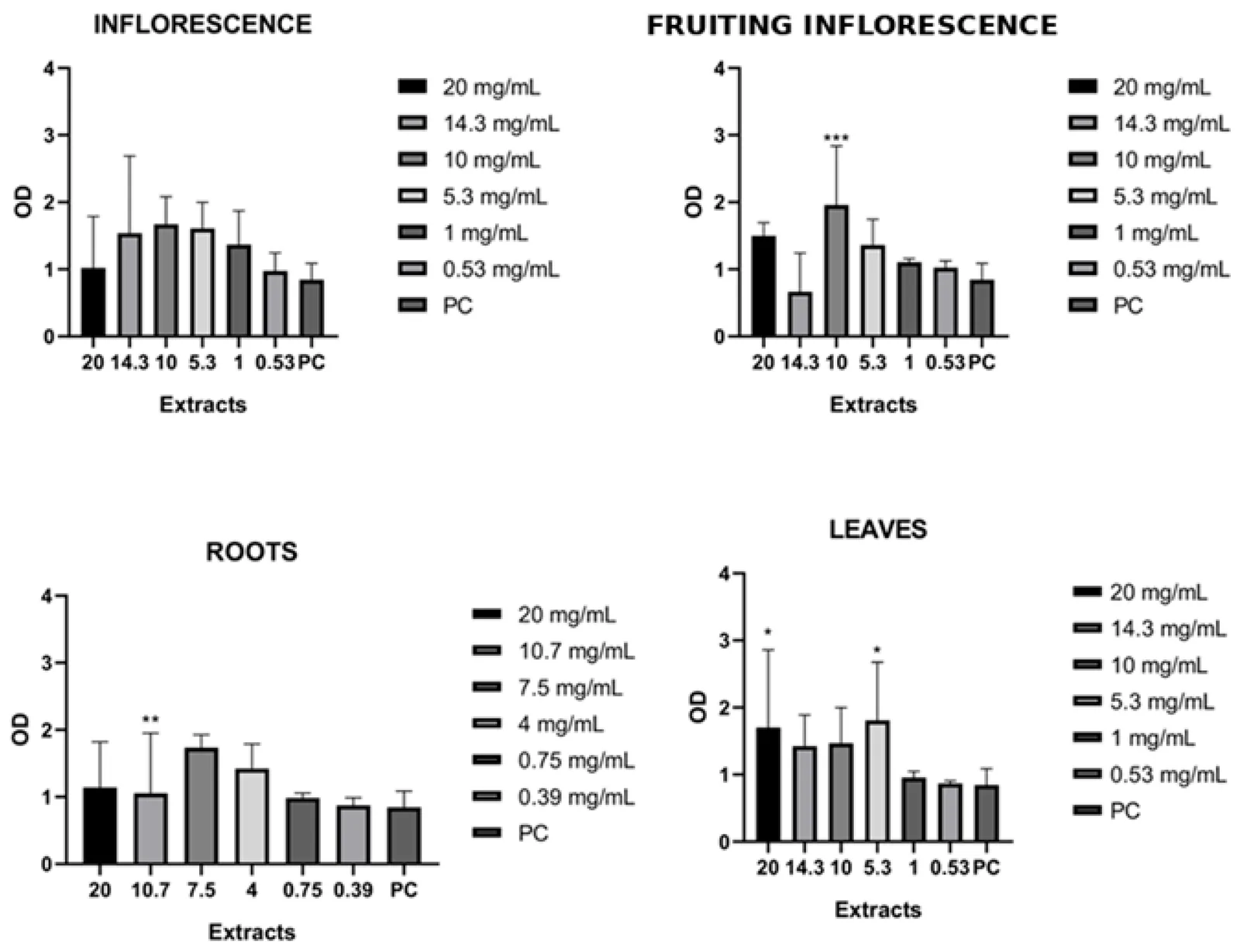
Biofilm biomass quantification of *Pseudomonas aeruginosa* treated with different concentrations of essential oils. Biofilm biomass was quantified using the Crystal Violet assay following 24 h exposure. All treated groups were statistically compared against the untreated growth control (Positive Control) using one-way ANOVA with Tukey’s post hoc test (* *p* < 0.05, ** *p* < 0.01, *** *p* < 0.001). Data represent mean ± standard deviation of four independent experiments (*n* = 4).

The effect of *P. falcata* essential oils on *P. aeruginosa* biofilm formation was evaluated using the crystal violet staining assay (Figure 3).

None of the tested essential oils produced a significant or concentration-dependent reduction in biofilm formation compared with the positive control. Minor variations in biofilm biomass were observed at individual concentrations, including slightly higher OD values at 10 mg/mL for the fruiting-inflorescence and root oils; however, these effects were not consistent across the different plant organs. Overall, the data indicate that *P. falcata* essential oils did not show significant antibiofilm activity against *P. aeruginosa* under the tested conditions.

Quantification of *P. aeruginosa* biofilm biomass revealed high experimental variability and minor localized increases in OD_595_ at certain intermediate essential oil concentrations. These localized elevations are attributed to non-specific Crystal Violet dye retention by lipophilic oil residues adhering to well surfaces rather than true biological biofilm stimulation. Overall, none of the essential oils demonstrated statistically robust or concentration-dependent reduction in established *P. aeruginosa* biofilms under the tested conditions.

## 3. Discussion

The essential oils of *P. falcata* showed marked compositional differences among the examined plant organs, although monoterpene hydrocarbons predominated in all samples. The root oil was strongly dominated by α-pinene (80.4%) and contained a relatively high proportion of *(E)*-nerolidol (7.1%), whereas the leaf oil was characterized mainly by myrcene (45.1%), α-pinene (15.9%), and *(Z)*-β-ocimene (14.3%). In comparison, the inflorescence and fruiting-inflorescence oils displayed more balanced monoterpene profiles, with α-pinene, α-phellandrene, limonene, camphene, and β-pinene among their principal constituents. Similar organ- and developmental-stage-related variation has been reported for Jordanian *P. palaestina*, in which α-pinene and myrcene predominated in the leaves, whereas ripe and unripe fruits contained higher proportions of *(E)*- and *(Z)*-ocimene and sabinene [20]. Seasonal changes reported for *P. lentiscus* essential oil also indicate that the volatile composition of *Pistacia* species may vary according to phenological and environmental conditions [17]. The differences observed here therefore represent compositional patterns associated with the sampled organs and should not, by themselves, be interpreted as evidence of organ-specific biological activity.

An important consideration is that the root material was obtained from a single individual, whereas the other plant organs were pooled from four individuals, because accessible aerial roots were observed only in one tree. Sampling roots from additional individuals would have required destructive excavation and could have caused damage or death to the plants. Therefore, root collection was intentionally limited to the available aerial roots in order to minimize harm to the natural population. Consequently, differences involving the root oil may reflect both tissue-related and inter-individual variation and cannot be attributed exclusively to organ type. This limitation is particularly relevant when comparing the chemical profile and biological activity of the root oil with those of the aerial organs.

The antibacterial assays showed a selective and relatively modest effect, restricted to *S. aureus* among the bacterial strains examined. No relevant activity was observed against the Gram-negative strains *E. coli* and *P. aeruginosa* under the conditions tested. Greater susceptibility of Gram-positive bacteria to essential oils has also been reported for other members of the genus, including *P. lentiscus* [23]. This difference is commonly associated with structural characteristics of the bacterial envelope. In Gram-negative bacteria, the outer membrane may act as an additional permeability barrier and restrict the access of hydrophobic constituents to cellular targets [24].

The high proportion of monoterpenes in the oils may have contributed to the activity observed against *S. aureus*. Terpenes and related volatile compounds have been reported to affect membrane integrity and permeability and, in some experimental systems, to interfere with cellular processes associated with adhesion, quorum sensing, and biofilm development [7,25]. Several constituents identified in *P. falcata*, including α-pinene, β-pinene, limonene, and *(E)*-nerolidol, have previously been associated with antimicrobial or antibiofilm effects [26,27,28,29,30,31,32]. These reports provide a plausible basis for considering such compounds as contributors to the activity of the oils; however, the mechanisms involved were not investigated in the present study. It therefore cannot be concluded that *P. falcata* essential oils act through the same mechanisms reported for their isolated constituents.

Differences in antibacterial activity among the oils further indicate that activity cannot be explained simply by the abundance of a single major compound. The inflorescence and fruiting-inflorescence oils were the most active against *S. aureus* (MIC = 5 mg/mL) and contained α-pinene together with appreciable proportions of α-phellandrene, limonene, camphene, β-pinene, and other constituents. The leaf oil, dominated by myrcene, α-pinene, and *(Z)*-β-ocimene, showed weaker activity (MIC = 10 mg/mL). In contrast, the root oil was inactive at the highest concentration tested (MIC > 20 mg/mL), despite containing 80.4% α-pinene and 7.1% *(E)*-nerolidol. The lack of activity of the α-pinene-rich root oil is particularly relevant because it indicates that the antibacterial effect cannot be directly related to the concentration of α-pinene alone. The overall chemical profile, including minor constituents and possible interactions among compounds, may influence the biological response. Nevertheless, isolated constituents and reconstituted mixtures were not evaluated in this study, and synergistic or antagonistic interactions therefore remain hypotheses that require experimental confirmation.

The growth kinetics results were consistent with the MIC findings and provided additional information on the duration of the antibacterial effect. The inflorescence oil produced sustained inhibition of *S. aureus* growth over 24 h, in agreement with its lower MIC value. In contrast, the root oil caused only an early transient reduction in growth, after which bacterial proliferation resumed, whereas the leaf oil produced an intermediate and concentration-dependent response. These patterns suggest that exposure to some oil constituents may delay bacterial growth without maintaining inhibition over longer periods, particularly at sub-inhibitory concentrations. Further experiments addressing membrane damage, cellular viability, leakage of intracellular components, or other bacterial responses would be useful to determine how these oils affect *S. aureus* growth.

Although activity was observed against planktonic *S. aureus*, none of the oils significantly inhibited *P. aeruginosa* growth, but changes in biofilm formation for *P. aeruginosa* were observed. This finding reinforces the distinction between inhibition of planktonic bacterial growth and interference with biofilm development. Biofilm formation involves several processes, including initial attachment, cell-to-cell communication, extracellular matrix production, and maturation, and the chemical requirements for disrupting these processes may differ from those required to inhibit planktonic growth. Although some compounds identified in the oils have previously been associated with antibiofilm activity [8,31,32,33,34,35,36], their presence was insufficient to produce a measurable effect against *P. aeruginosa* biofilm formation under the conditions used here. This may reflect their concentrations in the complete oils, limited access to relevant cellular or extracellular targets, interactions with other constituents, or the intrinsic resistance of *P. aeruginosa*. Studies using purified compounds, defined mixtures, broader concentration ranges, and complementary biofilm assays would help clarify these possibilities. Formulation approaches designed to improve the stability, dispersion, or delivery of volatile compounds may also be investigated, but their value should be established experimentally rather than assumed. Because the crude oils did not significantly inhibit *P. aeruginosa* biofilm formation, future studies could examine whether improved delivery systems enhance the availability of their volatile constituents. Nanomedicine-based approaches are being explored for biofilm-associated infections [33], while green-synthesized nanoparticles represent a separate antimicrobial and antibiofilm strategy [34].

From a plant perspective, the marked variation in volatile composition among roots, leaves, inflorescences, and fruiting inflorescences may also reflect differences in the physiological or ecological functions of these tissues. Terpenoid volatiles can participate in plant interactions with microorganisms, herbivores, pollinators, and the surrounding environment, and variation among organs may therefore be related to their distinct biological roles. The present chemical profiles provide a basis for future studies examining these functions in *P. falcata*. At the same time, the antibacterial activity detected in vitro indicates that the oils contain compounds of potential applied interest. Such findings should be regarded as an initial basis for further investigation rather than evidence of therapeutic efficacy. Evaluation against a broader panel of microorganisms, together with toxicity, selectivity, mechanistic, fractionation, and compound-combination studies, would be required before any potential biomedical application could be considered.

Overall, *P. falcata* essential oils were chemically variable among the examined organs and showed selective activity against the Gram-positive strain *S. aureus*, while little or no antibacterial effect was detected against the Gram-negative strains tested. The greater activity of the inflorescence and fruiting-inflorescence oils, together with the lack of activity of the α-pinene-rich root oil, suggests that antibacterial efficacy is related to the overall composition of the oil rather than to the abundance of a single constituent. The results expand the chemical and biological information available for *P. falcata* and identify its floral oils as suitable candidates for further investigation, while the contribution of individual constituents and their interactions remains to be established.

## 4. Materials and Methods

### 4.1. Bacterial Strains and Culture Media

The antibacterial and antibiofilm activities of the essential oils were evaluated against *Staphylococcus aureus* NCTC 6571, *Escherichia coli* MG1655, and *Pseudomonas aeruginosa* PAO1. Bacteria were cultured on Mueller–Hinton agar (MHA), and Mueller–Hinton broth (MHB) was used for minimum inhibitory concentration (MIC) assays. The antibiotics chloramphenicol, kanamycin, vancomycin and ciprofloxacin (Sigma-Aldrich, Arklow, Ireland) were used as positive controls.

### 4.2. Plant Material

**Figure 4:**
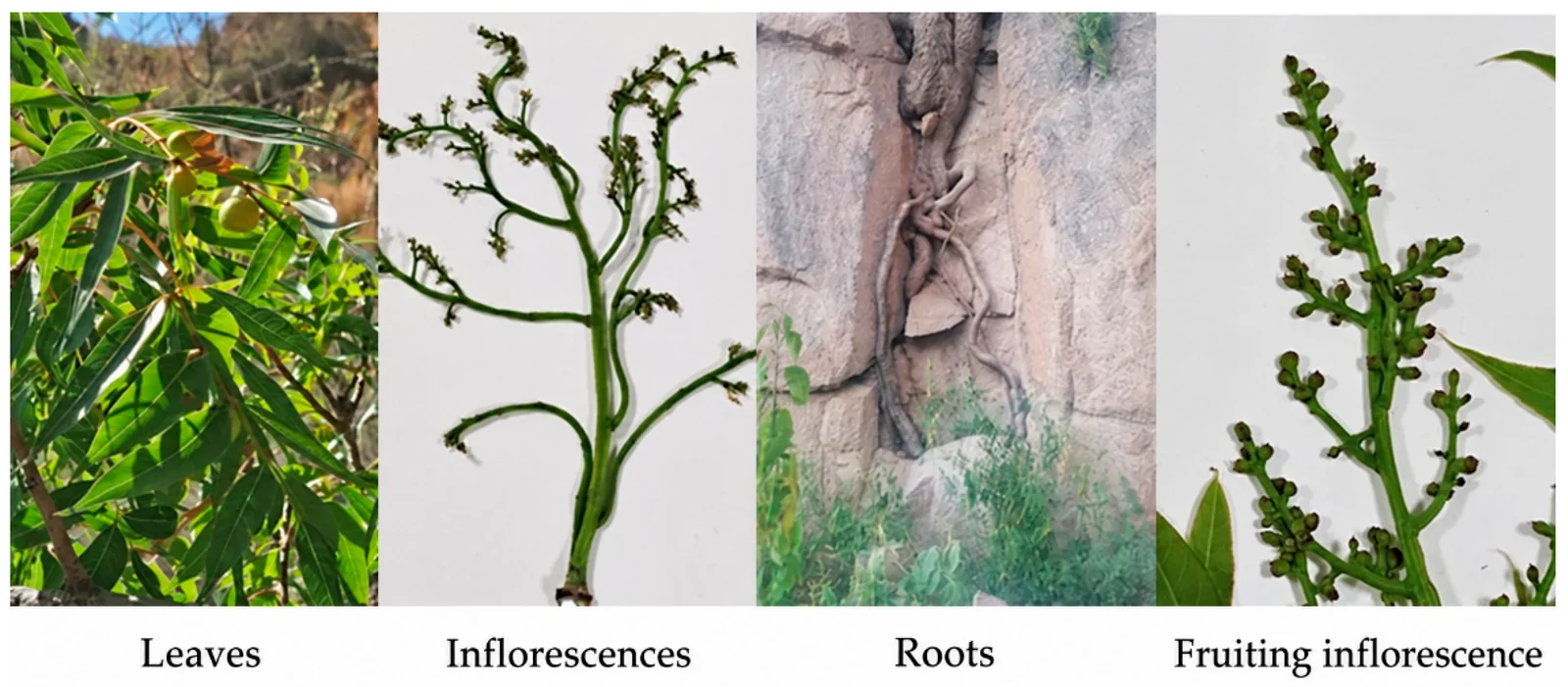
Different organs of *P. falcata*.

Leaves, inflorescences, and fruiting inflorescences of *P. falcata* Figure 4 were collected from four adult, well-developed individuals growing at Wadi Thee Gazal GPS coordinates: 21°05′31.31″ N, 40°21′28.13″ E; elevation: 2012 m a.s.l, approximately 30 km south-west of Taif city in the Makkah Region, Saudi Arabia. Samples from the four individuals were pooled separately according to plant organ. Root material was collected from one individual only, using a naturally exposed root growing over the rock surface, thereby avoiding excavation and minimizing damage to the plant. A voucher specimen (SA-IE/2023-1) was deposited in the Herbarium of Pharmacognosy Lab at the Faculty of Pharmacy, Umm Al-Qura University.

### 4.3. Essential Oil Hydrodistillation

Fresh plant material (200 g from each organ) was finely cut and subjected separately to hydrodistillation in a Clevenger-type apparatus. Essential oil yields were 0.22% (leaves), 0.17% (roots), 0.29% (inflorescences), and 0.27% (fruiting inflorescences).

### 4.4. Gas Chromatography-Mass Spectrometry Analyses and Peak Identification

GC–EIMS analysis was carried out using an Agilent 7890B gas chromatograph coupled to a 5977B single-quadrupole mass spectrometer (Agilent Technologies, Santa Clara, CA, USA). Separation was achieved on an HP-5MS capillary column (30 m × 0.25 mm i.d., 0.25 µm film thickness; Agilent Technologies). Instrumental settings and operating conditions followed a previously published method [35]. Each essential-oil sample was analysed by GC–MS in triplicate (*n* = 3), and relative abundances are reported as mean ± SD. Experimental LRIs were calculated according to van den Dool and Kratz [36] using a homologous C8–C28 *n*-alkane series analysed under the same chromatographic conditions.

Chromatographic peaks were integrated manually, and the EI mass spectrum of each retained peak was individually inspected and searched against the NIST 14 and Adams libraries; evident siloxane and phthalate contaminants were excluded. Compounds identification was further supported by comparison of experimental retention indices with literature values, including those reported by Adams [37] and Davies [38].

Candidate identities were retained when the manually reviewed EI mass spectrum and experimental LRI were concordant with library and literature data, rather than on the basis of a library match value alone. Authentic reference standards previously analysed under the GC–MS method were used to construct an in-house library of retention times, LRIs, and EI mass spectra. When a reference product contained multiple constituents, these were chromatographically resolved, individually identified, and entered as separate library records. Assignments not additionally verified by an authentic reference standard are reported as tentative. Results are presented in Table 1.

### 4.5. Antibacterial and Antibiofilm Assays

#### 4.5.1. Minimum Inhibitory Concentration (MIC) and Minimum Bactericidal Concentration (MBC) Assays

Bacterial inocula were prepared from strains stored at −80 °C. Each strain was first streaked onto Mueller–Hinton agar (MHA) plates and incubated overnight at 37 °C. A fresh subculture was then prepared on a new MHA plate and incubated under the same conditions. This fresh culture was used for all subsequent assays.

For inoculum preparation, colonies were suspended in 6 mL of Mueller–Hinton broth (MHB), and the bacterial suspension was adjusted to a 0.5 McFarland standard (OD600 ≈ 0.08–0.10). This suspension was adjusted with sterile 0.9% (*w*/*v*) saline solution. After that, it was subsequently diluted 1:180 into fresh Mueller–Hinton Broth (MHB) to yield the working inoculum. Each microplate well received 90 µL of this inoculated MHB, resulting in a final bacterial density of approximately 5 × 10^5^ CFU/mL per well.

The antibacterial activity of the essential oils was evaluated using a modified broth microdilution assay in 96-well microplates according to CLSI guidelines with adaptations. To minimize potential toxic effects of the solvent on bacterial growth, essential oils were initially solubilized in dimethyl sulfoxide (DMSO) to obtain standardized stock solutions and intermediate working dilutions (Table 3).

Briefly, 90 µL of sterile Mueller–Hinton Broth (MHB) was added to each well before sample inoculation. To establish the testing gradient, specific aliquots of Working Dilution 1 (15, 10, and 5 µL) and Working Dilution 2 (10 and 5 µL) were transferred into the wells, yielding final assay volumes ranging from 95 to 105 µL. Exact final concentrations and residual DMSO percentages (*v*/*v*) per well were calculated based on the total final volume in each well, as detailed in (Table S1) For clarity and reporting consistency throughout the manuscript and figures, rounded concentrations of ≈14.3, 10.0, 5.3, 1.0, and 0.53 mg/mL were assigned (or ≈10.7, 7.5, 4.0, 0.75, and 0.39 mg/mL for sample D3).

To evaluate the antibacterial activity at a higher maximum concentration of 20.0 mg/mL, specific aliquots of each crude stock solution were added directly to wells containing 90 µL of inoculated MHB (MHB mixed with the bacterial suspension): 8.3 µL for inflorescences (D1, stock 237.90 mg/mL), 2.9 µL for fruiting inflorescence (D2, stock 650.80 mg/mL, 22.5 µL for roots (D3, stock 100.00 mg/m), and 2.7 µL for leaves (D4, stock 681.00 mg/mL), giving final well volumes ranging between 92.7 and 112.5 µL. The remaining testing concentrations (14.3; 10.0; 5.3; 1 and 0.53 mg/mL) were obtained by adding volumes of Working Dilution 1 and Working Dilution 2 into 90 µL of inoculated MHB, as summarized in Table 3 and detailed in Table S1.

Untreated bacterial culture in MHB served as a positive growth control (Table S1). Vehicle controls were evaluated by adding 5 µL, 10 µL, and 15 µL of pure DMSO into 90 µL of inoculated MHB, corresponding to final solvent concentrations of 5.26%, 10.0%, and 14.28% v/v, respectively. Vehicle controls up to 10.0% v/v DMSO supported normal bacterial growth, establishing the threshold for non-inhibitory solvent exposure. While solvent exposure at 14.28% v/v caused partial growth inhibition, the highest tested condition for root essential oil ($D_{3}$, 20.0 mg/mL, containing 16.67% v/v DMSO) still exhibited bacterial growth, leading to its classification as inactive (MIC>20.0 mg/mL). Consequently, high solvent presence did not induce false-positive antibacterial efficacy, although solvent contribution to baseline optical density changes at this specific concentration is acknowledged as a limitation.

Reference antibiotics were included as positive controls and tested using standard two-fold serial dilutions according to CLSI guidelines. Vancomycin was tested over a concentration range of 1–32 µg/mL against *S. aureus* NCTC 6571, kanamycin at 1.5–50 µg/mL against *E. coli* MG1655, chloramphenicol at 3.12–50 µg/mL against both *S. aureus* NCTC 6571 and *E. coli* MG1655, and ciprofloxacin at 0.5–32 µg/mL against *P. aeruginosa* PAO1. MIC values for the reference antibiotics were determined using the same endpoint criterion applied to the essential oils.

Plates were incubated at 37 °C for 18 h, and MIC values were defined as the lowest concentrations showing no visible bacterial growth.

For MBC determination, 5 µL from each well showing no visible growth was transferred onto MHA plates and incubated at 37 °C for 18 h. Controls included untreated bacteria, DMSO-treated bacteria, and standard antibiotics. All assays were performed in triplicate.

#### 4.5.2. Growth Kinetics Assay

Time-dependent effects on bacterial growth were assessed using the same microdilution conditions described for the MIC assay. The 96-well plates were incubated in a plate reader at 37 °C for 24 h. MIC values were determined visually after 18 h incubation at 37 °C based on bacterial turbidity in comparison to growth controls. For real-time growth kinetics (Figure 2), ${\mathrm{OD}}_{600}$ was measured hourly over 24 h using a microplate reader. Immediately prior to each hourly reading, the microplate was automatically agitated with 10 s of orbital shaking to ensure homogeneous suspension and minimize essential oil aggregation or settling. Background absorbance was subtracted using uninoculated media-oil blanks at matching concentrations. Because ${\mathrm{OD}}_{600}$ readings reflect culture turbidity and cannot fully distinguish viable cell proliferation from bacterial aggregation or physical emulsion effects, kinetic profiles were evaluated in conjunction with visual MIC and MBC determinations. The initial reading (time zero) was used as the baseline, and subsequent readings were expressed relative to this value.

#### 4.5.3. Biofilm Formation Assay

The effect of essential oils (EOs) on biofilm biomass was evaluated using a microtiter plate assay based on Shukla and Rao [18], performed immediately following the minimum inhibitory concentration (MIC) determination. Briefly, serial dilutions of the EOs, as described at 4.5.1, as well as a reference antibiotic as a positive control, were prepared in 96-well plates containing Mueller–Hinton broth.

Bacterial cultures were inoculated as described for the MIC assay. Wells containing culture medium and bacterial inoculum without essential oil served as the growth control (untreated control), while wells containing only sterile culture medium served as the negative/blank control to account for background absorbance from non-specific crystal violet binding.

Biofilm formation by *Pseudomonas aeruginosa* PAO1 was evaluated directly in 96-well microtiter plates following a 18 h incubation at 37 °C. After recording planktonic kinetic growth, non-adherent bacteria were gently removed by aspiration, and wells were washed three times with 200 µL of sterile 0.9% NaCl solution. Adherent biofilms were fixed, stained with 0.1% (*w*/*v*) crystal violet for 15 min, and washed with distilled water. The bound dye was solubilized in absolute ethanol, and absorbance was measured at 595 nm (OD_595_) using a microplate reader. Uninoculated broth wells containing matching essential oil concentrations served as background controls. All assays were conducted in four independent experiments (*n* = 4) performed on different days.

## 5. Conclusions

This study provides the first report on essential oils obtained from different organs of *P. falcata*. The chemical composition varied substantially among plant parts but was consistently dominated by monoterpene hydrocarbons.

The inflorescence and fruiting-inflorescence oils showed the highest antibacterial activity against *S. aureus*, whereas the leaf oil was less active and the root oil was inactive under the tested conditions. No meaningful inhibition of *E. coli* and *P. aeruginosa* was observed. However, it was only observed for *P. aeruginosa* biofilm formation. Nevertheless, these findings help define the biological activity of the essential oils and indicate that their effects were selective rather than broadly antibacterial or antibiofilm. Their activity appears to be selective, with a greater effect against *S. aureus* than against Gram-negative bacteria. The differences among plant organs also suggest that the antibacterial effect depends on the overall composition of each oil rather than on one major compound alone.

Future studies should test the oils against a wider range of Gram-positive bacteria, including clinical and antibiotic-resistant *S. aureus* strains. It would also be useful to examine the activity of individual compounds, possible interactions among them, and their effects when combined with conventional antibiotics. Time-kill and viability assays could provide a clearer picture of how the oils act. Since the crude oils had limited effects on *P. aeruginosa* biofilms, further work should focus on purified constituents, selected compound combinations, improved delivery systems, and other biofilm models.

## Figures and Tables

**Table 1:** Chemical composition of the essential oils from different organs of *P. falcata*.

Constituent	RT (min)	LRI_exp_	LRI_lit_	Leaves (%)	Fruiting Inflorescences (%)	Inflorescences (%)	Roots (%)
*n*-octane	2.99	800	800 ^a^	-	-	0.1 ± 0.0	-
*(E)*-2-hexenal ^c^	3.72	856	855 ^a^	0.3 ± 0.2	-	-	-
*(Z)*-3-hexen-1-ol ^c^	3.76	857	857 ^a^	0.4 ± 0.2	-	-	-
*(E)*-2-hexen-1-ol ^c^	3.92	863	862 ^a^	0.1 ± 0.0	-	-	-
heptanal ^c^	4.62	901	901 ^a^	-	-	0.1 ± 0.0	-
tricyclene	5.13	928	926 ^a^	0.3 ± 0.1	2.1 ± 0.3	1.8 ± 0.3	0.1 ± 0.0
α-thujene	5.24	933	931 ^a^	-	-	-	0.4 ± 0.1
α-pinene ^c^	5.46	941	939 ^a^	15.9 ± 2.2	27.8 ± 0.7	28.3 ± 1.0	80.4 ± 1.2
α-fenchene	5.77	954	953 ^a^	-	-	-	0.2 ± 0.0
camphene ^c^	5.78	955	953 ^a^	1.1 ± 0.3	6.2 ± 0.3	5.5 ± 0.5	0.9 ± 0.0
sabinene ^c^	6.45	977	976 ^a^	-	1.1 ± 0.1	0.6 ± 0.1	0.3 ± 0.1
β-pinene ^c^	6.53	982	980 ^a^	1.2 ± 0.2	5.2 ± 0.4	4.8 ± 0.4	4.0 ± 0.1
myrcene ^c^	7.10	993	991 ^a^	45.1 ± 2.8	11.2 ± 0.3	2.1 ± 0.3	1.3 ± 0.1
α-phellandrene ^c^	7.37	1006	1005 ^a^	3.8 ± 0.4	19.7 ± 0.4	10.1 ± 0.5	0.4 ± 0.0
α-terpinene ^c^	7.72	1020	1019 ^a^	-	0.1 ± 0.0	0.1 ± 0.0	0.1 ± 0.0
*p*-cymene ^c^	7.95	1028	1028 ^a^	0.5 ± 0.0	1.7 ± 0.0	4.9 ± 0.4	0.3 ± 0.0
limonene ^c^	8.11	1032	1031 ^a^	3.5 ± 0.1	12.3 ± 1.1	9.5 ± 0.6	1.8 ± 0.0
β-phellandrene ^c^	8.21	1033	1031 ^a^	-	2.6 ± 0.4	1.4 ± 0.1	-
*(Z)*-β-ocimene ^d^	8.45	1042	1039 ^a^	14.3 ± 0.6	3.6 ± 0.3	4.1 ± 0.4	-
*(E)*-β-ocimene ^d^	8.76	1052	1050 ^a^	4.3 ± 0.2	0.9 ± 0.1	1.0 ± 0.2	-
γ-terpinene ^c^	9.12	1062	1060 ^a^	-	-	0.1 ± 0.0	0.2 ± 0.0
terpinolene ^c^	10.18	1090	1088 ^a^	-	-	0.3 ± 0.0	0.3 ± 0.0
linalool ^c^	10.60	1101	1098 ^a^	-	0.7 ± 0.0	0.1 ± 0.0	-
nonanal ^c^	10.76	1102	1102 ^a^	-	-	0.4 ± 0.1	-
*cis*-*p*-menth-2-en-1-ol	11.42	1123	1121 ^a^	-	-	0.3 ± 0.1	-
*trans*-pinocarveol ^c^	12.09	1141	1141 ^a^	-	-	-	0.2 ± 0.1
*trans*-*p*-menth-2-en-1-ol	12.11	1142	1136 ^b^	-	-	0.2 ± 0.0	-
*cis*-verbenol ^c^	12.19	1142	1142 ^a^	-	-	-	0.1 ± 0.0
*trans*-verbenol	12.32	1143	1144 ^a^	-	-	-	0.4 ± 0.1
camphene hydrate	12.49	1150	1149 ^a^	-	-	0.2 ± 0.1	-
borneol ^c^	13.16	1167	1166 ^a^	-	0.1 ± 0.0	0.2 ± 0.0	-
4-terpineol ^c^	13.64	1179	1179 ^a^	-	0.2 ± 0.0	0.7 ± 0.1	0.4 ± 0.0
α-terpineol ^c^	14.19	1191	1191 ^a^	-	0.1 ± 0.0	1.9 ± 0.1	0.2 ± 0.0
myrtenal ^c^	14.41	1194	1195 ^b^	-	-	-	0.2 ± 0.1
verbenone ^c^	14.94	1205	1205 ^a^	-	-	-	0.2 ± 0.0
*cis*-carvenone oxide	17.29	1263	1261 ^a^	-	-	0.3 ± 0.2	-
bornyl acetate	18.15	1287	1286 ^a^	1.9 ± 0.8	2.3 ± 0.0	4.8 ± 0.4	-
pinanediol	19.41	1317	1316 ^a^	-	0.4 ± 0.1	-	-
δ-elemene	20.32	1338	1335 ^b^	-	-	0.2 ± 0.2	-
β-elemene	22.58	1392	1392 ^a^	-	-	0.3 ± 0.1	-
β-caryophyllene ^c^	23.63	1419	1419 ^a^	0.3 ± 0.3	-	-	-
α-humulene ^c^	25.00	1455	1455 ^a^	0.2 ± 0.3	-	-	-
alloaromadendrene	25.30	1462	1461 ^a^	-	-	0.1 ± 0.1	-
γ-muurolene	25.96	1478	1477 ^a^	0.2 ± 0.2	-	-	-
germacrene D	26.12	1482	1480 ^a^	1.2 ± 1.2	0.5 ± 0.1	2.1 ± 0.4	-
β-selinene	26.33	1487	1493 ^a^	-	-	0.2 ± 0.0	-
viridiflorene	26.69	1494	1494 ^a^	0.4 ± 0.4	-	-	-
bicyclogermacrene	26.71	1496	1495 ^a^	0.3 ± 0.3	0.2 ± 0.0	2.4 ± 0.4	-
cuparene	27.09	1505	1505 ^a^	-	-	0.2 ± 0.0	-
δ-cadinene	27.81	1524	1524 ^a^	0.4 ± 0.4	-	-	-
germacrene B	29.06	1557	1557 ^a^	0.3 ± 0.4	0.1 ± 0.1	1.6 ± 0.3	-
*(E)*-nerolidol ^d^	29.39	1564	1564 ^a^	0.4 ± 0.5	0.1 ± 0.1	0.3 ± 0.0	7.1 ± 1.3
ledol	29.45	1565	1565 ^a^	-	-	0.1 ± 0.0	-
spathulenol	29.87	1576	1578 ^a^	-	-	0.9 ± 0.2	0.1 ± 0.0
globulol	30.09	1584	1589 ^a^	0.2 ± 0.4	-	0.3 ± 0.0	-
viridiflorol	30.38	1591	1592 ^a^	0.2 ± 0.3	-	0.9 ± 0.2	-
rosifoliol	30.78	1601	1603 ^a^	0.1 ± 0.1	-	-	-
1-*epi*-cubenol	31.81	1629	1628 ^a^	-	-	0.3 ± 0.1	-
γ-eudesmol	31.88	1630	1632 ^a^	0.2 ± 0.2	-	0.3 ± 0.1	-
T-cadinol	32.27	1641	1641 ^a^	0.2 ± 0.3	-	-	-
T-muurolol	32.44	1642	1640 ^b^	0.1 ± 0.1	-	0.6 ± 0.1	-
β-eudesmol ^c^	32.54	1650	1649 ^a^	0.2 ± 0.3	-	-	-
α-eudesmol	32.65	1652	1653 ^a^	0.4 ± 0.4	-	-	-
α-cadinol	32.73	1653	1654 ^a^	0.3 ± 0.3	-	2.5 ± 0.4	-
*(E,E)*-farnesol ^c^	35.19	1723	1722 ^a^	0.1 ± 0.2	-	-	-
*m*-camphorene	42.93	1960	1960 ^a^	0.7 ± 0.8	-	-	-
*p*-camphorene	44.01	1995	1995 ^a^	0.3 ± 0.1	-	-	-
Monoterpene hydrocarbons				89.9 ± 6.3	94.5 ± 0.4	74.6 ± 3.1	90.7 ± 1.4
Oxygenated monoterpenes				1.9 ± 0.8	3.8 ± 0.1	8.7 ± 0.4	1.7 ± 0.2
Sesquiterpene hydrocarbons				3.3 ± 3.4	0.8 ± 0.2	7.0 ± 1.5	0.0 ± 0.0
Oxygenated sesquiterpenes				2.4 ± 2.9	0.1 ± 0.1	6.2 ± 1.0	7.2 ± 1.3
Diterpenes				1.0 ± 0.7	0.0 ± 0.0	0.0 ± 0.0	0.0 ± 0.0
Non-terpene derivatives				0.8 ± 0.4	0.0 ± 0.0	0.6 ± 0.1	0.0 ± 0.0
Total identified				99.3 ± 0.3	99.2 ± 0.3	97.1 ± 0.4	99.6 ± 0.1

Values are relative percentages (mean ± SD, *n* = 3); –, not detected. RT, retention time under the reported GC conditions; LRIexp, experimental linear retention index calculated according to van den Dool and Kratz using an n-alkane series from C8 to C28 analysed under the same chromatographic conditions; LRIlit, literature retention index. Identification was based on concordant LRI and EI-MS data. ^a^ NIST Chemistry WebBook: temperature-programmed non-polar capillary data on phases comparable to HP-5MS. ^b^ Adams, Identification of Essential Oil Components by Gas Chromatography/Mass Spectrometry, ed. 4.1 (DB-5). ^c^ Additional verification against an in-house RT/LRI/EI-MS entry generated from an injected authentic reference standard. ^d^ Additional verification against a separate in-house entry generated after chromatographic resolution and individual identification of the constituent in a reference mixture.

**Table 2:** MIC and MBC of *P. falcata* essential oils against *S. aureus*.

Sample	MIC (mg/mL)	MBC (mg/mL)
Inflorescences	5.3 (range: 10–5.3)	5.3 (range: 10–5.3)
Fruiting inflorescences	5.3	5.3
Roots	>20	>20
Leaves	10	10

**Table 3:** Stock solutions from crude stock and working dilutions of essential oils.

				Crude Stock Solution	Working Dilution 1	Working Dilution 1	Working Dilution 2	Working Dilution 2
Sample	Plant Part	Mass (mg)	DMSO (μL)	Conc. (mg/mL)	Conc. (mg/mL)	DMSO (%)	Conc. (mg/mL)	DMSO (%)
D1	Inflorescence	237.9	762.1	237.90	100.00	32.77%	10.23	3.28%
D2	Fruiting inflorescences	650.8	350.0	650.80	100.00	5.60%	10.41	0.56%
D3	Roots	100.0	900.0	100.00	75.00	67.50%	7.50	6.75%
D4	Leaves	681.0	319.0	681.00	100.00	5.10%	10.90	0.51%

MHB: Mueller–Hinton Broth; DMSO: Dimethyl sulfoxide. a Working Dilution 1 (Dil. 1): Prepared in 200 μL total volume with MHB. b Working Dilution 2 (Dil. 2): Prepared via 1:10 serial dilution of Dilution 1 in MHB.

## Data Availability

All data are included in the article.

## Supplementary Materials

The following supporting information can be downloaded at: https://www.mdpi.com/article/10.3390/molecules31193487/s1, File S1: experimental EI mass spectra available from the archived GC–MS data for 67 identified constituents (PDF) (Table S1 and Figure S1–S67); Data S1, corresponding spectra in NIST MSP format.
