# Supplementary material for: Chemical Composition and Antibacterial Activity of Essential Oils from Different Organs of Pistacia falcata
Source: Molecules. 2026 Oct 1;31(19):3487. doi: 10.3390/molecules31193487 (PMC13666642; doi:10.3390/molecules31193487)

Chemical Composition and Antibacterial Activity of Essential Oils from Different Organs of *Pistacia falcata*

Content:

1. **Table S1. Final concentrations of essential oils and residual DMSO in 96-well microplate assay wells (Table S1)**
2. **Data Figures:S1-S67: Experimental EI mass spectra available from the archived GC–MS data; Data S1: corresponding mass spectra in NIST MSP format.**

1. Table S1. Final concentrations of essential oils and residual DMSO in 96-well microplate assay wells.

Calculated exact and rounded nominal concentrations per well after adding specific sample aliquots to 90 µL of MHB broth.

	**15 µL of Dil. 1 (105 µL Total)[a]**		**10 µL of Dil. 1 (100 µL Total)[b]**		**5 µL of Dil. 1 (95 µL Total)[c]**		**10 µL of Dil. 2 (100 µL Total)[d]**		**5 µL of Dil. 2 (95 µL Total)[e]**	
**Sample**	**Exact (Nominal)**	**DMSO (%)**	**Exact (Nominal)**	**DMSO (%)**	**Exact (Nominal)**	**DMSO (%)**	**Exact (Nominal)**	**DMSO (%)**	**Exact (Nominal)**	**DMSO (%)**
**D1**	14.29 (**14.3**) mg/mL	14.29%	10.00 (**10.0**) mg/mL	10.00%	5.26 (**5.3**) mg/mL	5.26%	1.00 (**1.0**) mg/mL	1.00%	0.53 (**0.53**) mg/mL	0.53%
**D2**	14.29 (**14.3**) mg/mL	14.29%	10.00 (**10.0**) mg/mL	10.00%	5.26 (**5.3**) mg/mL	5.26%	1.00 (**1.0**) mg/mL	1.00%	0.53 (**0.53**) mg/mL	0.53%
**D3**	10.71 (**10.7**) mg/mL	14.29%	7.50 (**7.5**) mg/mL	10.00%	3.95 (**4.0**) mg/mL	5.26%	0.75 (**0.75**) mg/mL	1.00%	0.39 (**0.39**) mg/mL	0.53%
**D4**	14.29 (**14.3**) mg/mL	14.29%	10.00 (**10.0**) mg/mL	10.00%	5.26 (**5.3**) mg/mL	5.26%	1.00 (**1.0**) mg/mL	1.00%	0.53 (**0.53**) mg/mL	0.53%

MHB: Mueller-Hinton Broth; **DMSO:** Dimethyl sulfoxide.

[a] 15 µL of Dilution 1 added to 90 µL of MHB broth (Total volume = 105 µL).

[b] 10 µL of Dilution 1 added to 90 µL of MHB broth (Total volume = 100 µL).

[c] 5 µL of Dilution 1 added to 90 µL of MHB broth (Total volume = 95 µL).

[d] 10 µL of Dilution 2 added to 90 µL of MHB broth (Total volume = 100 µL).

[e] 5 µL of Dilution 2 added to 90 µL of MHB broth (Total volume = 95 µL).

2. Figures S1-S67

n-octane Experimental LRI: 800

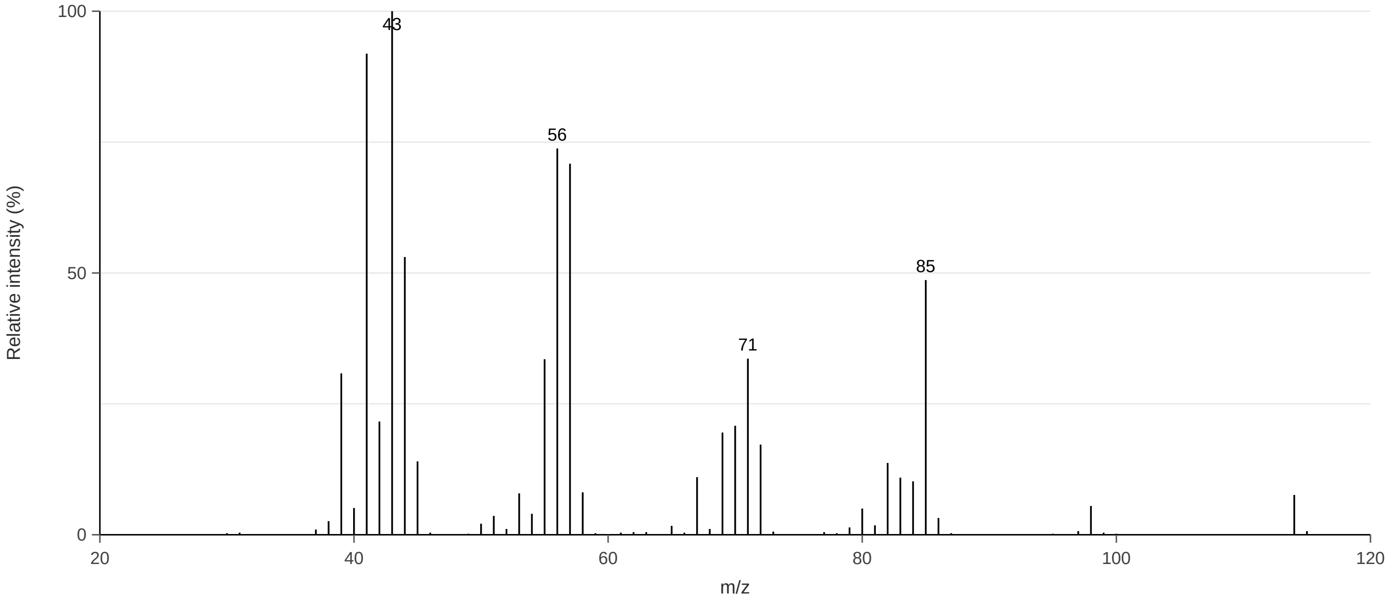

(E)-2-hexenal Experimental LRI: 856

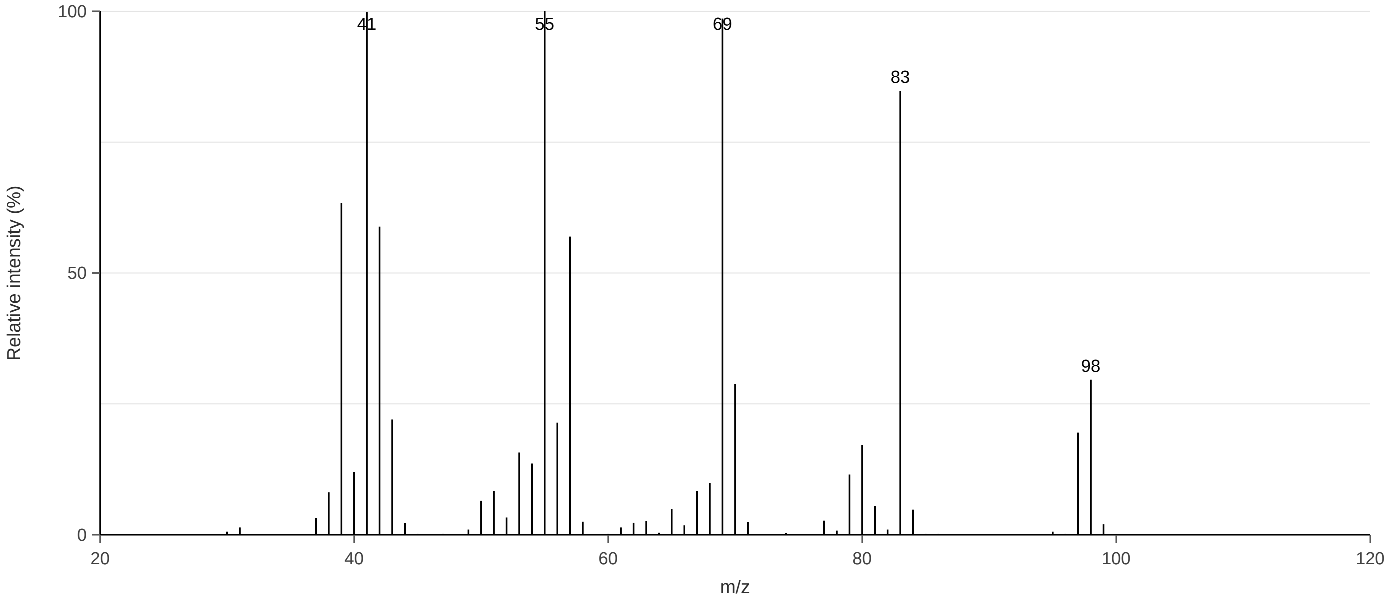

(Z)-3-hexen-1-ol Experimental LRI: 857

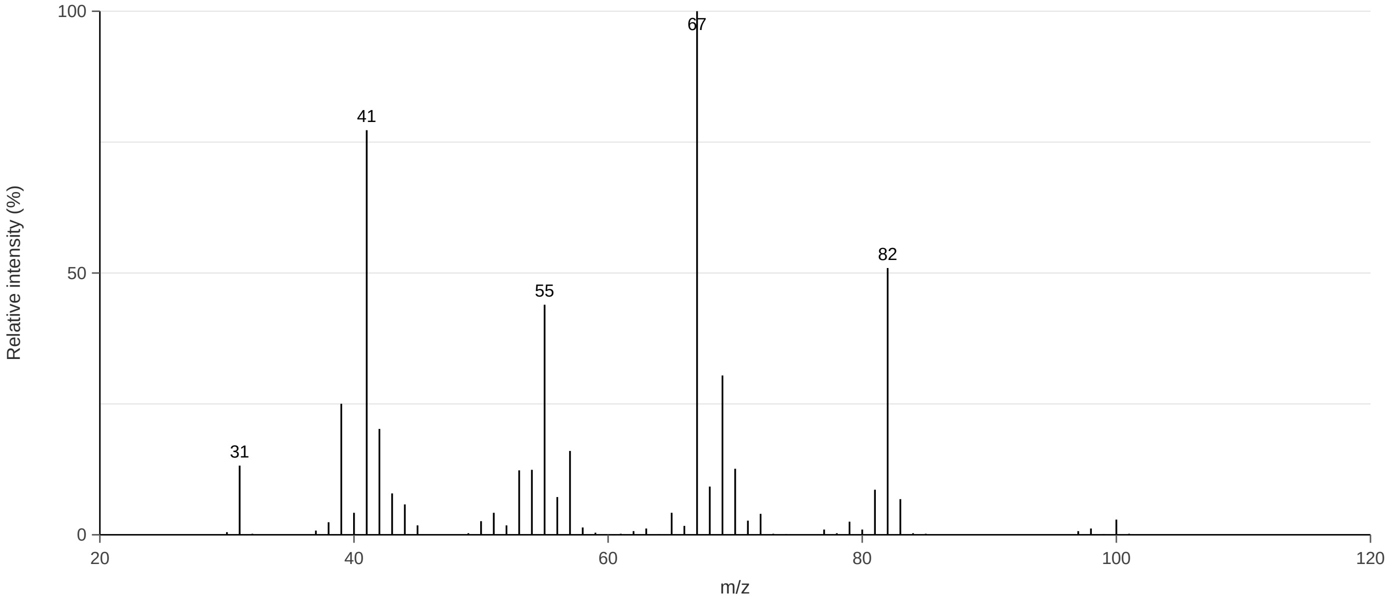

(E)-2-hexen-1-ol Experimental LRI: 863

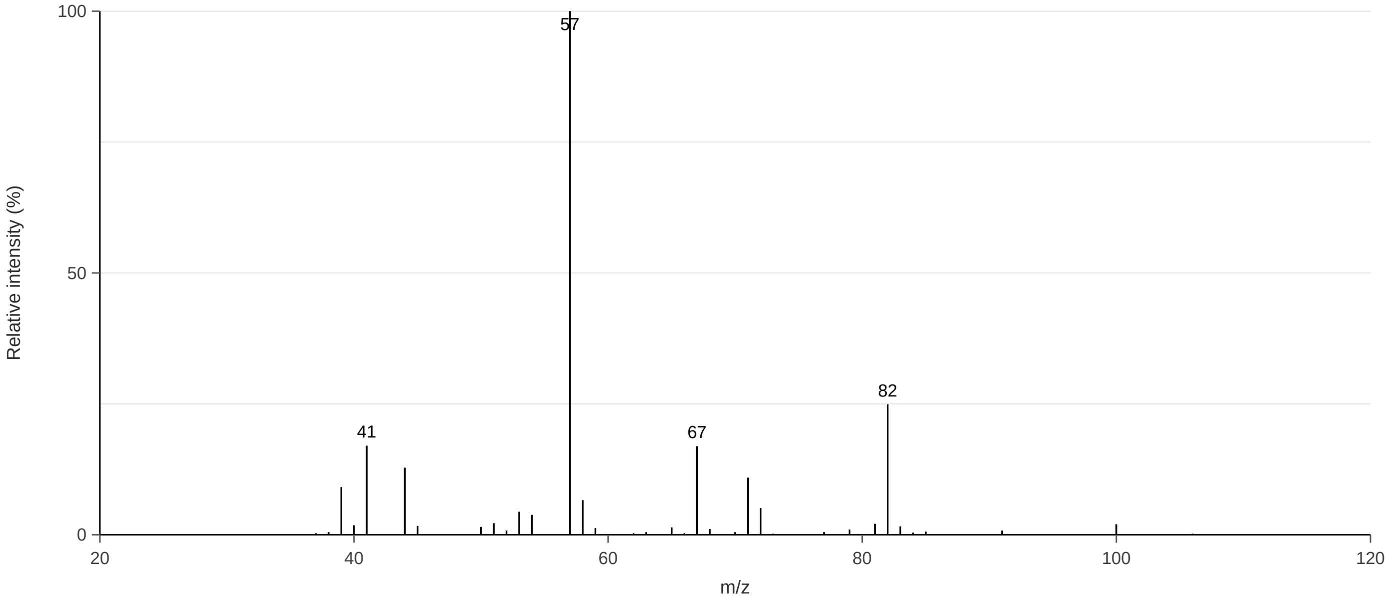

heptanal Experimental LRI: 901

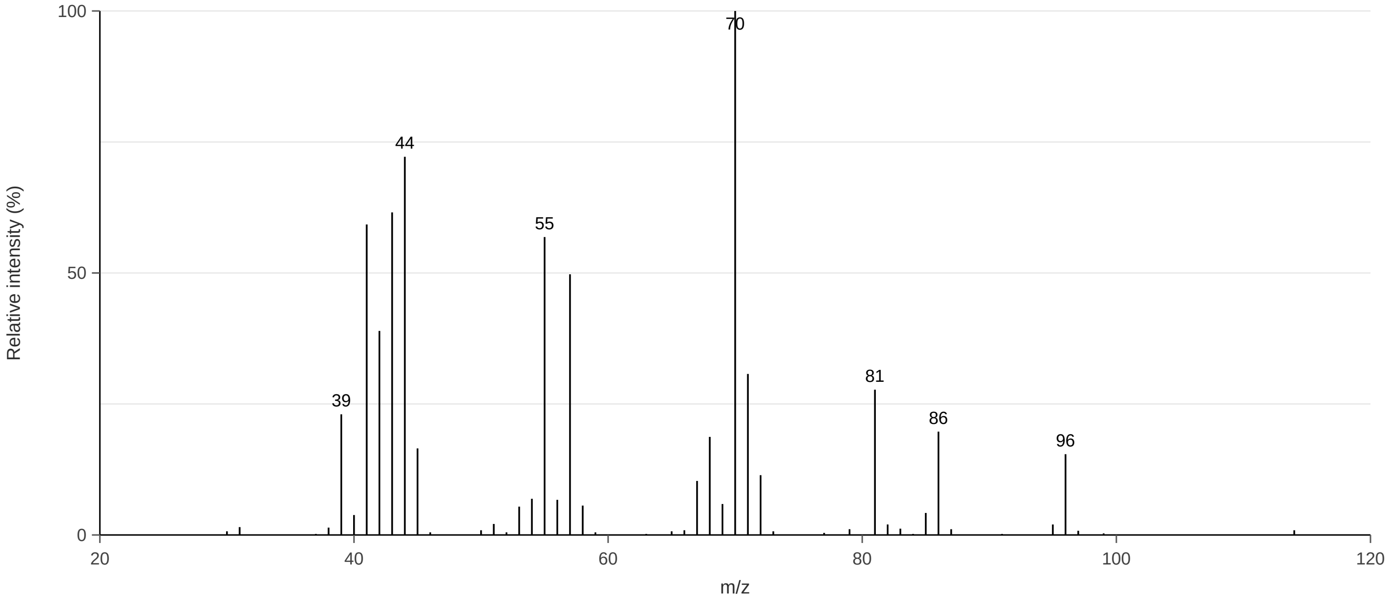

tricyclene Experimental LRI: 928

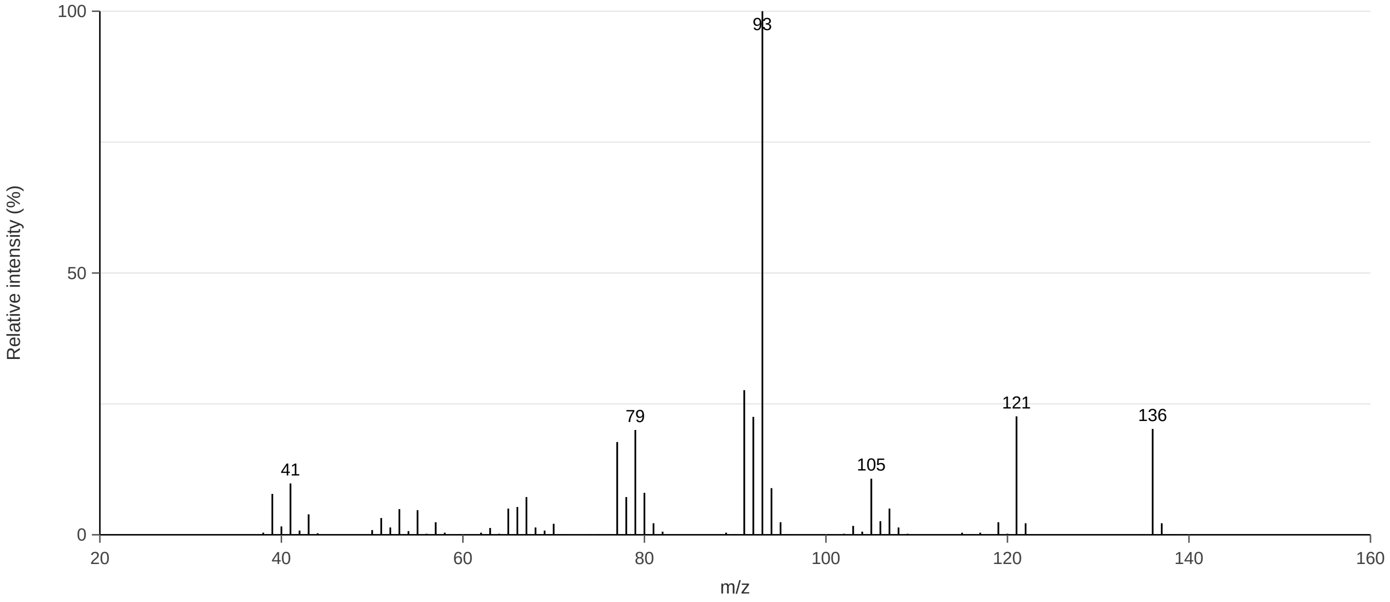

alpha-thujene

Experimental LRI: 933

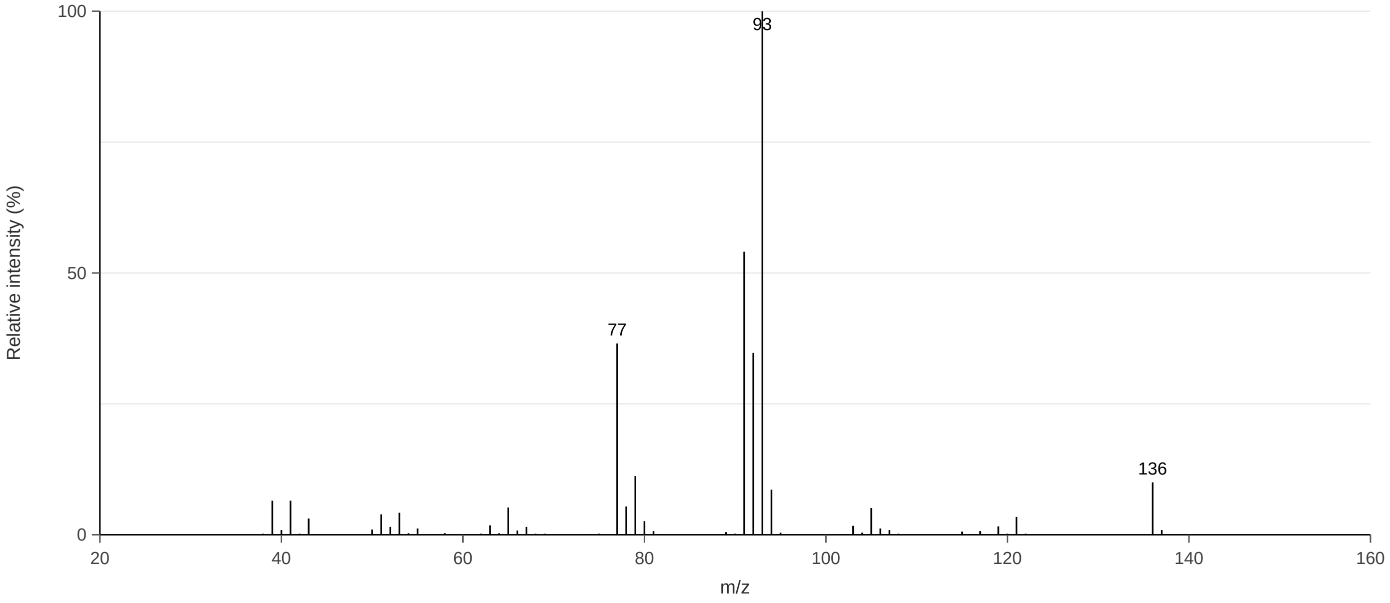

alpha-pinene

Experimental LRI: 941

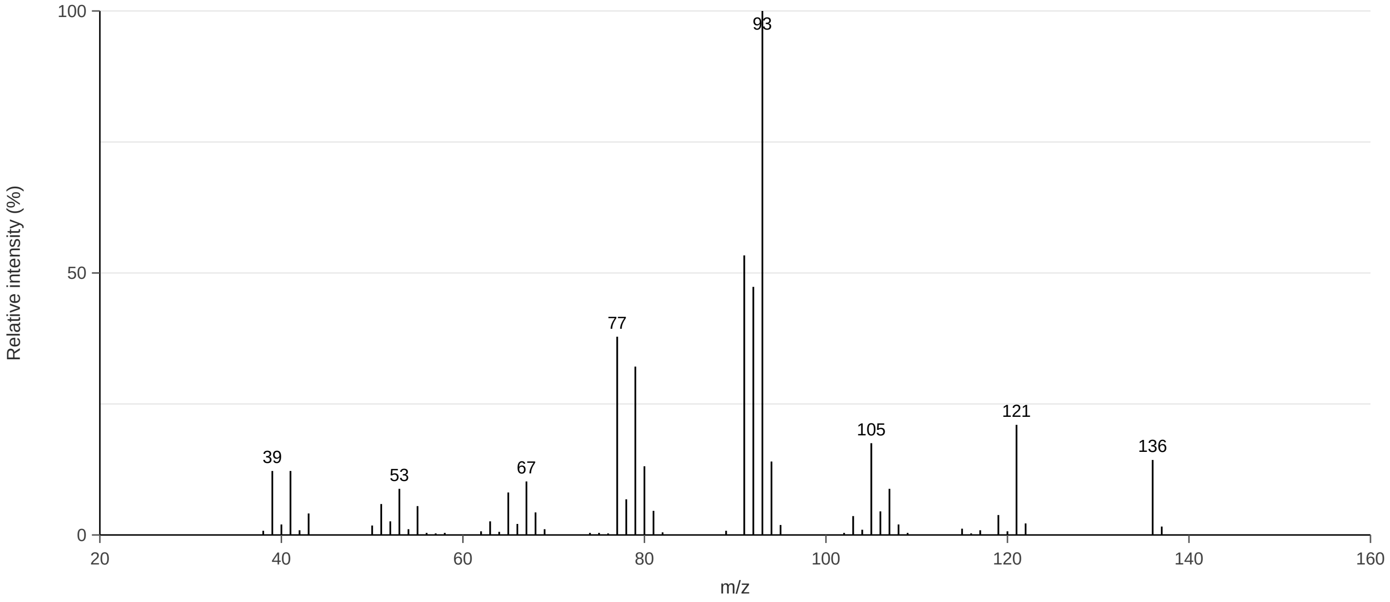

alpha-fenchene

Experimental LRI: 954

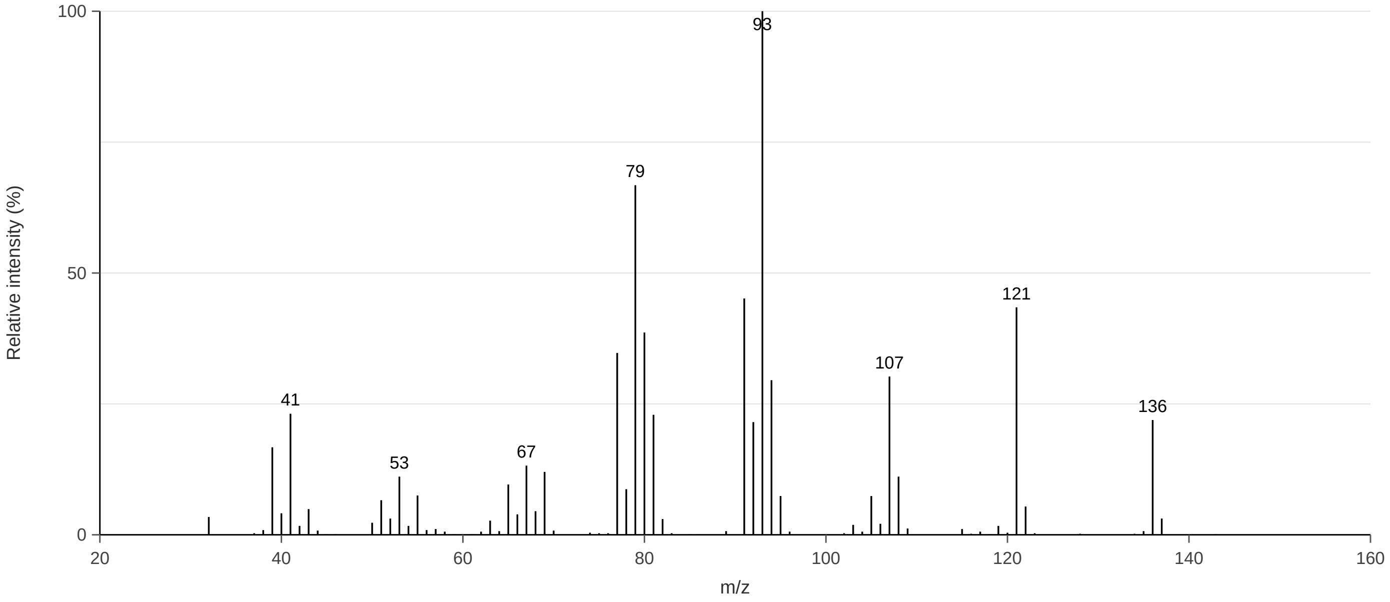

camphene

Experimental LRI: 955

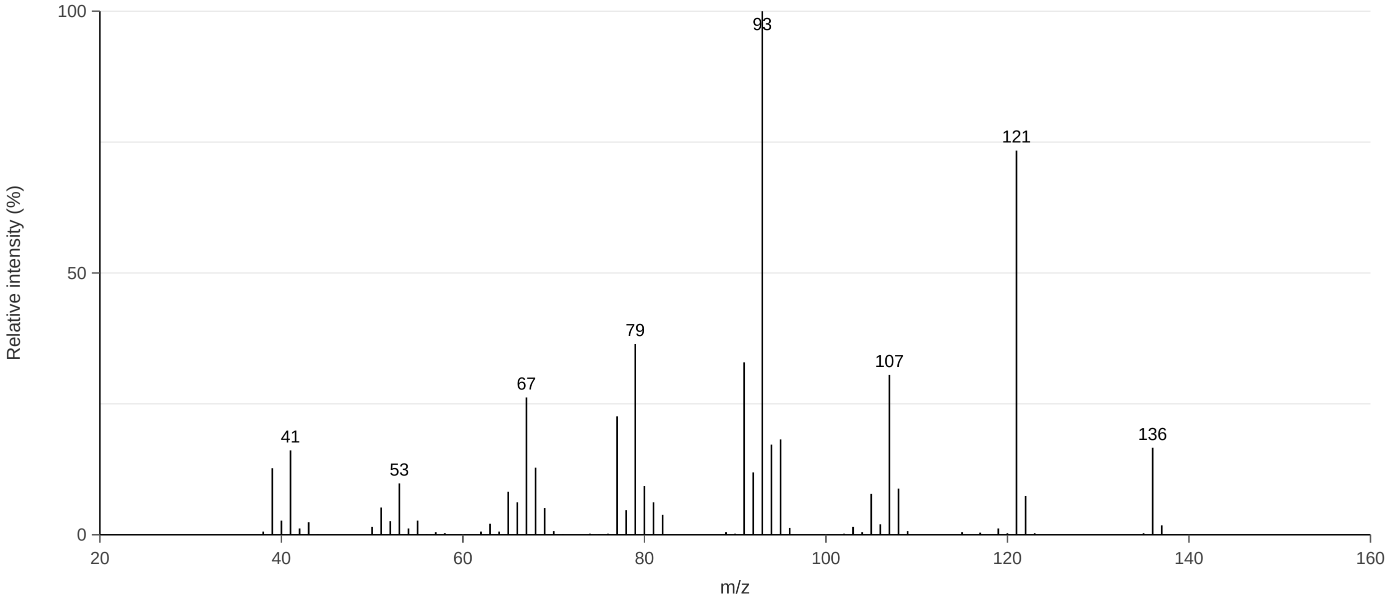

sabinene

Experimental LRI: 977

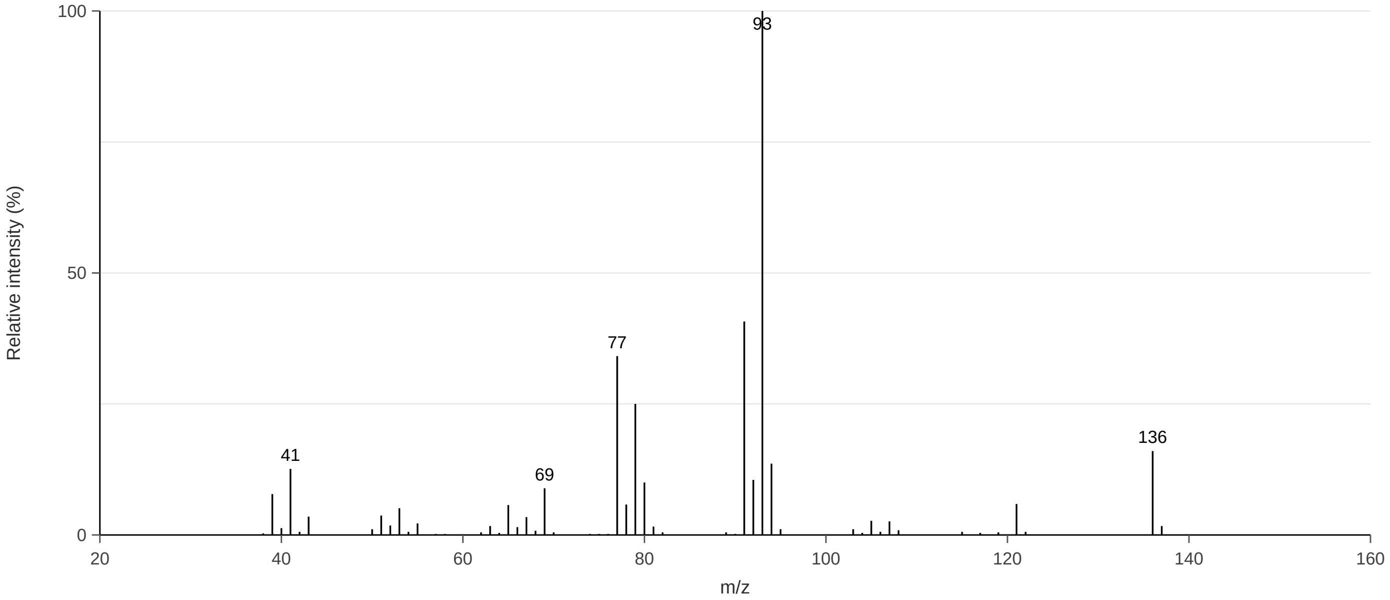

beta-pinene

Experimental LRI: 982

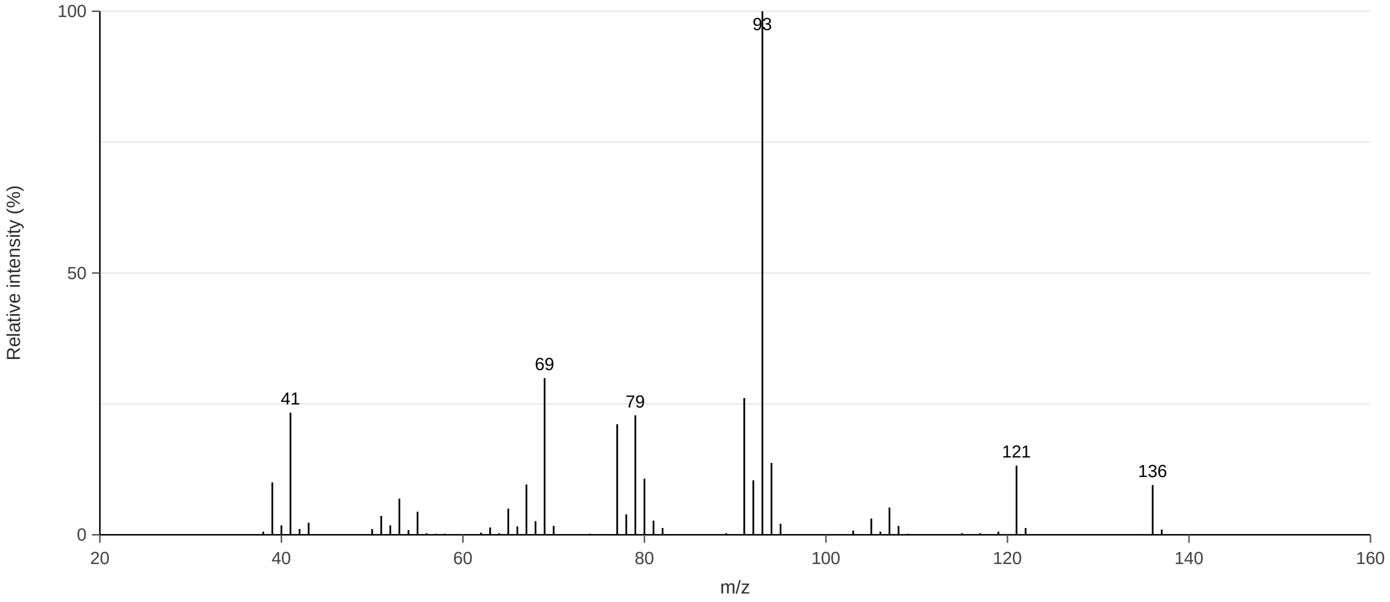

myrcene

Experimental LRI: 993

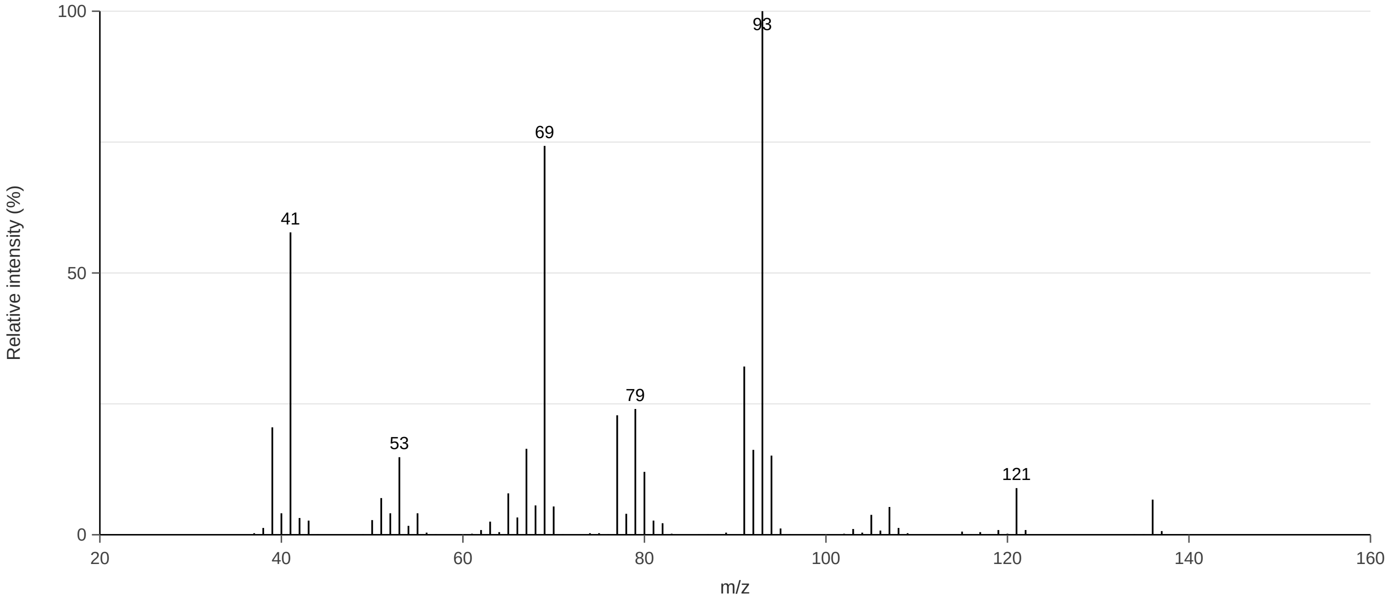

alpha-phellandrene

Experimental LRI: 1006

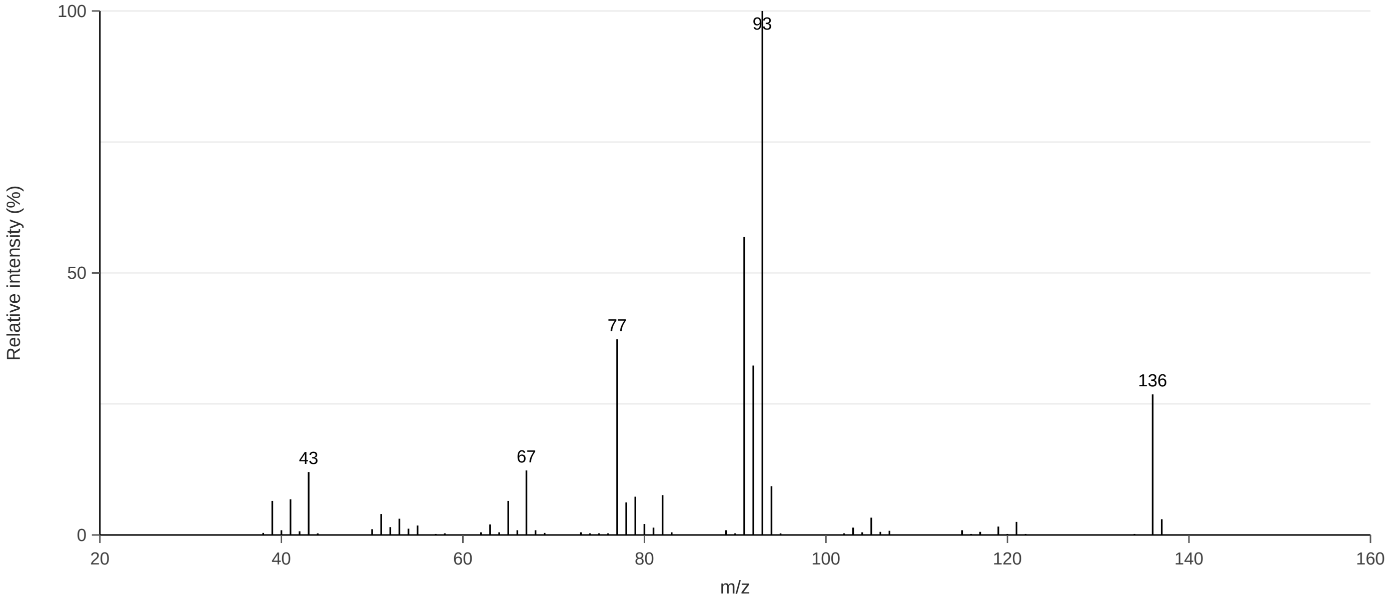

alpha-terpinene

Experimental LRI: 1020

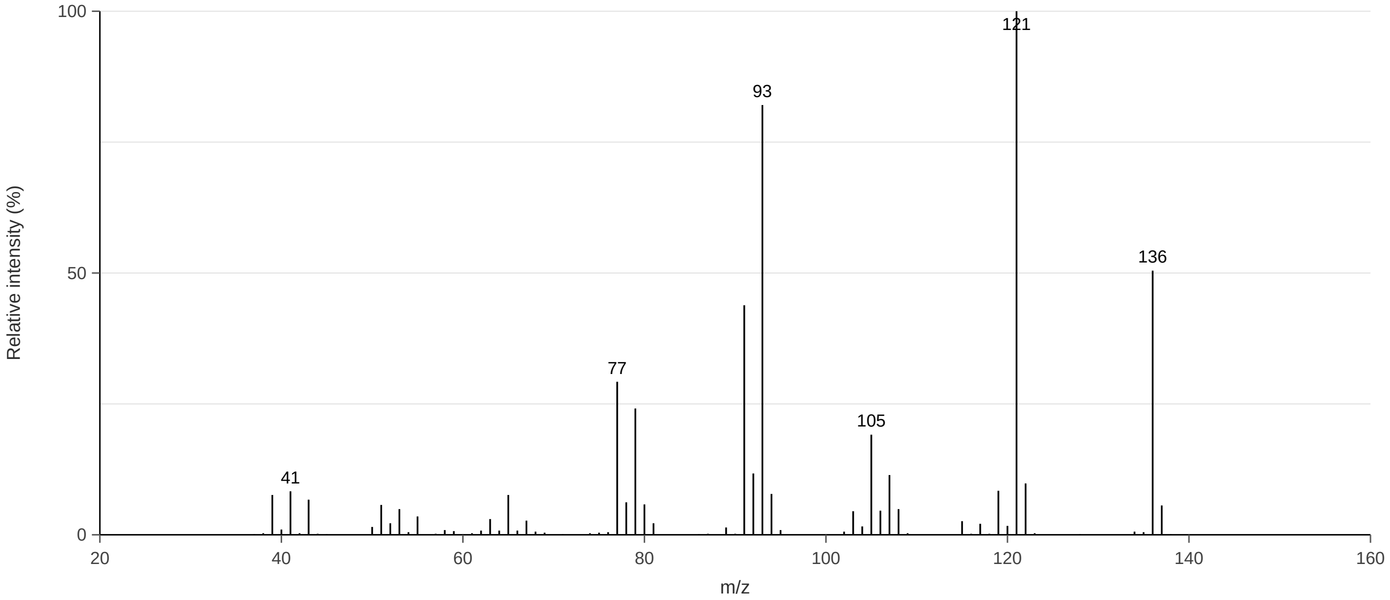

p-cymene

Experimental LRI: 1028

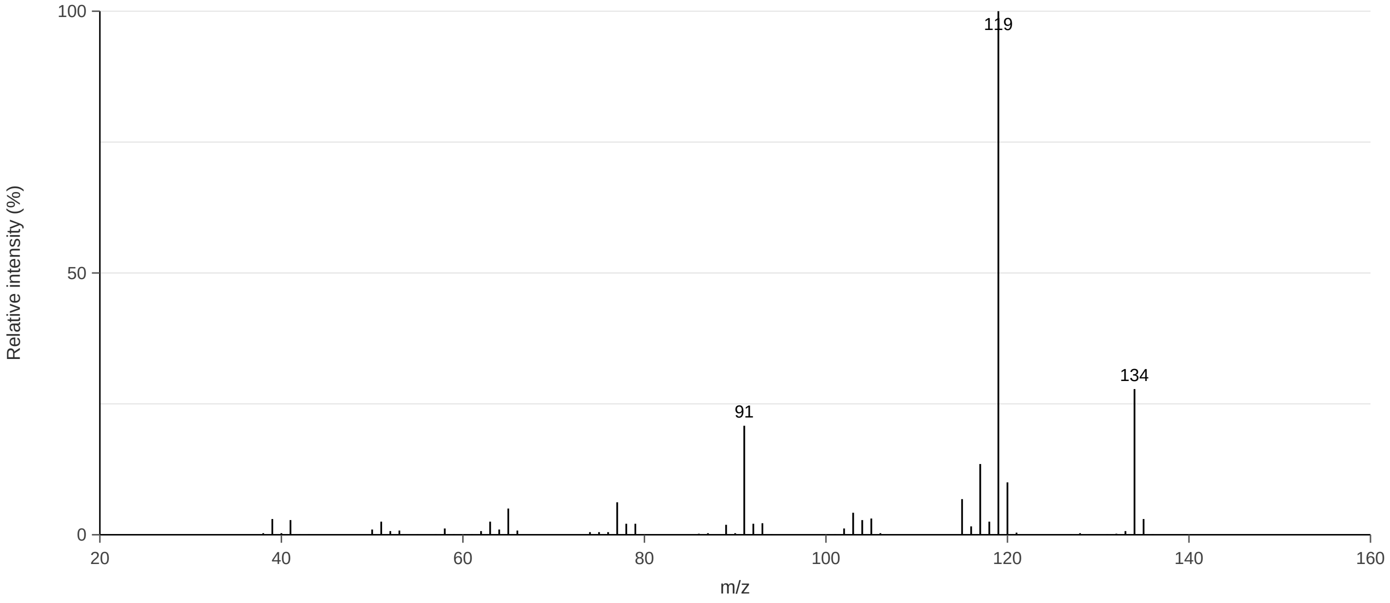

limonene

Experimental LRI: 1032

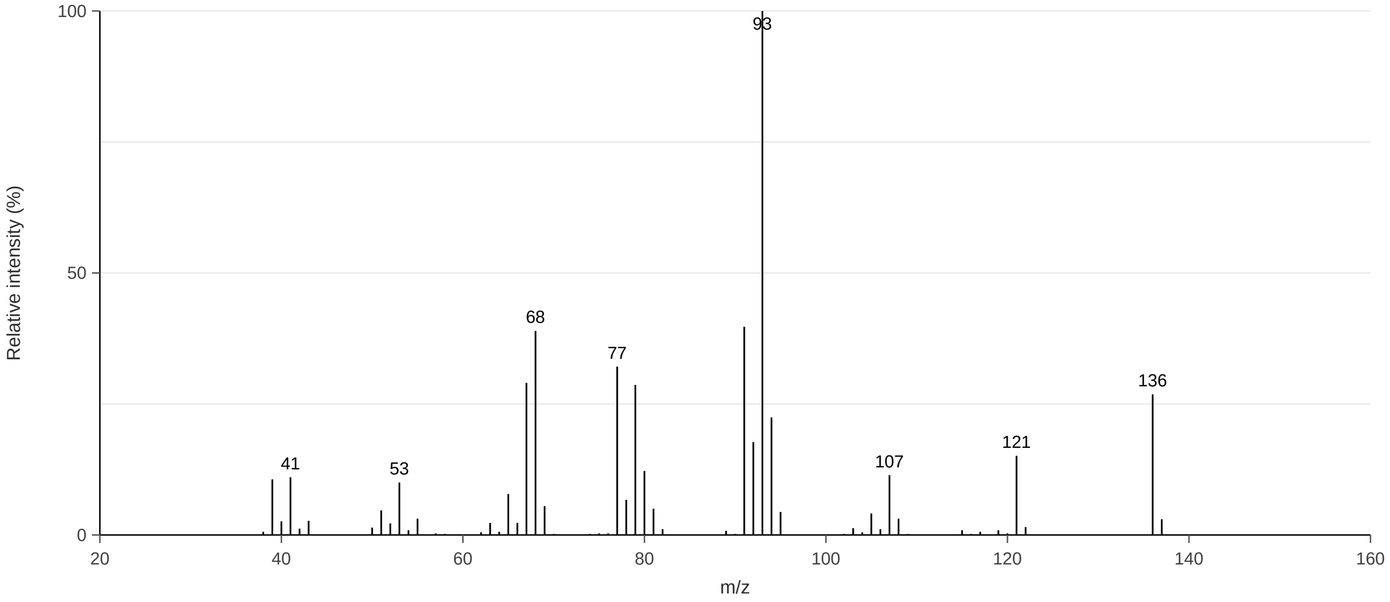

beta-phellandrene

Experimental LRI: 1033

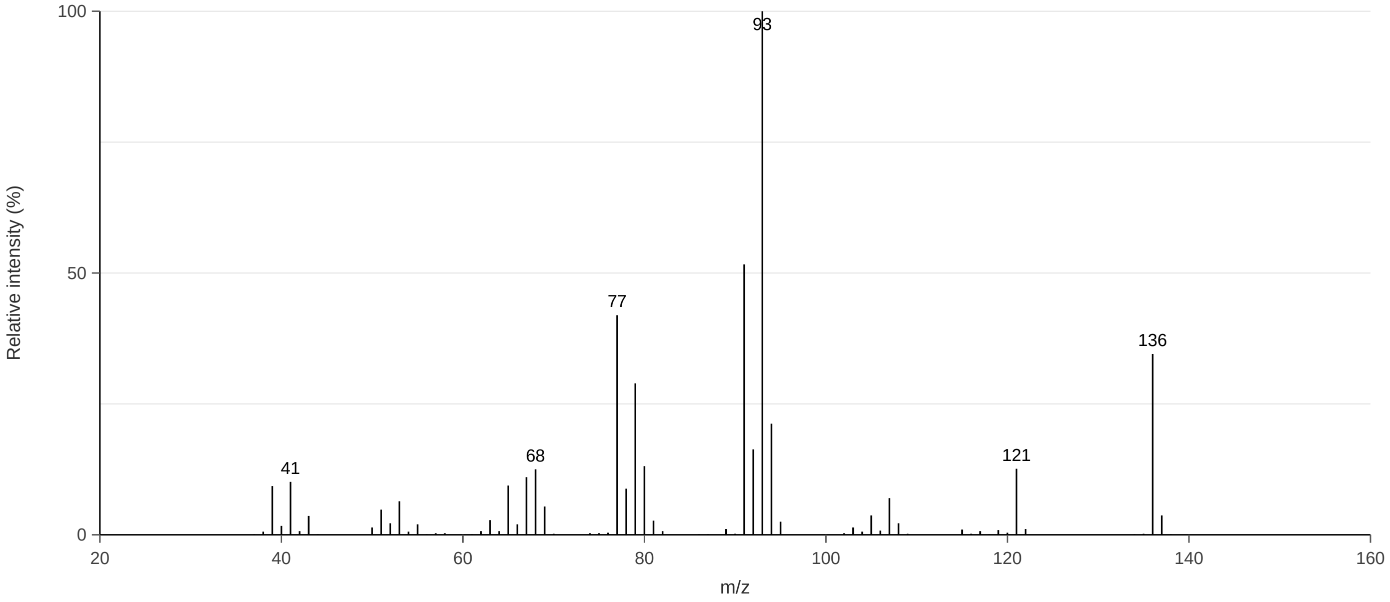

(Z)-beta-ocimene

Experimental LRI: 1042

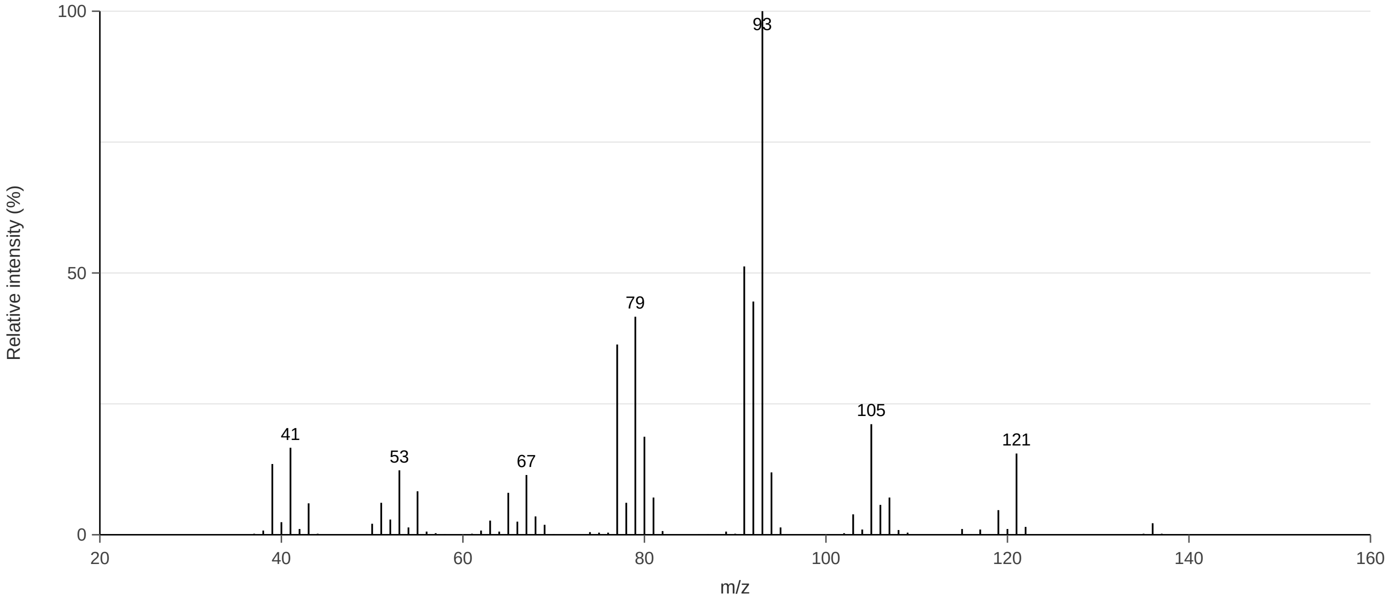

(E)-beta-ocimene

Experimental LRI: 1052

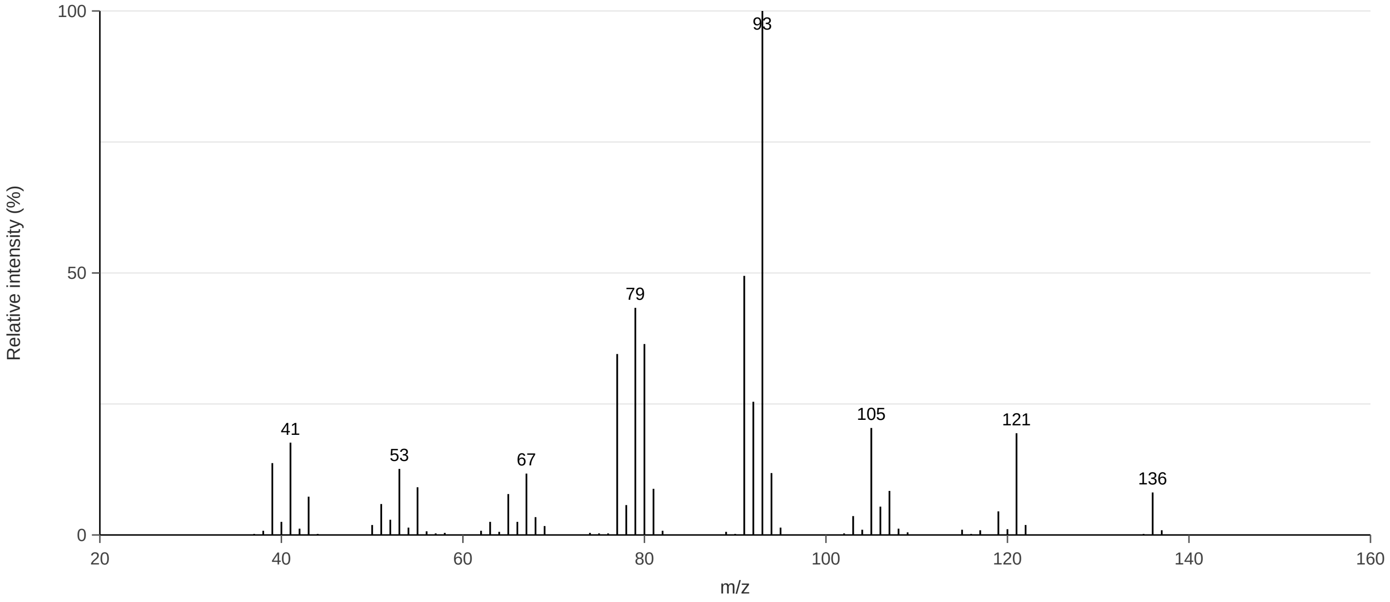

gamma-terpinene

Experimental LRI: 1062

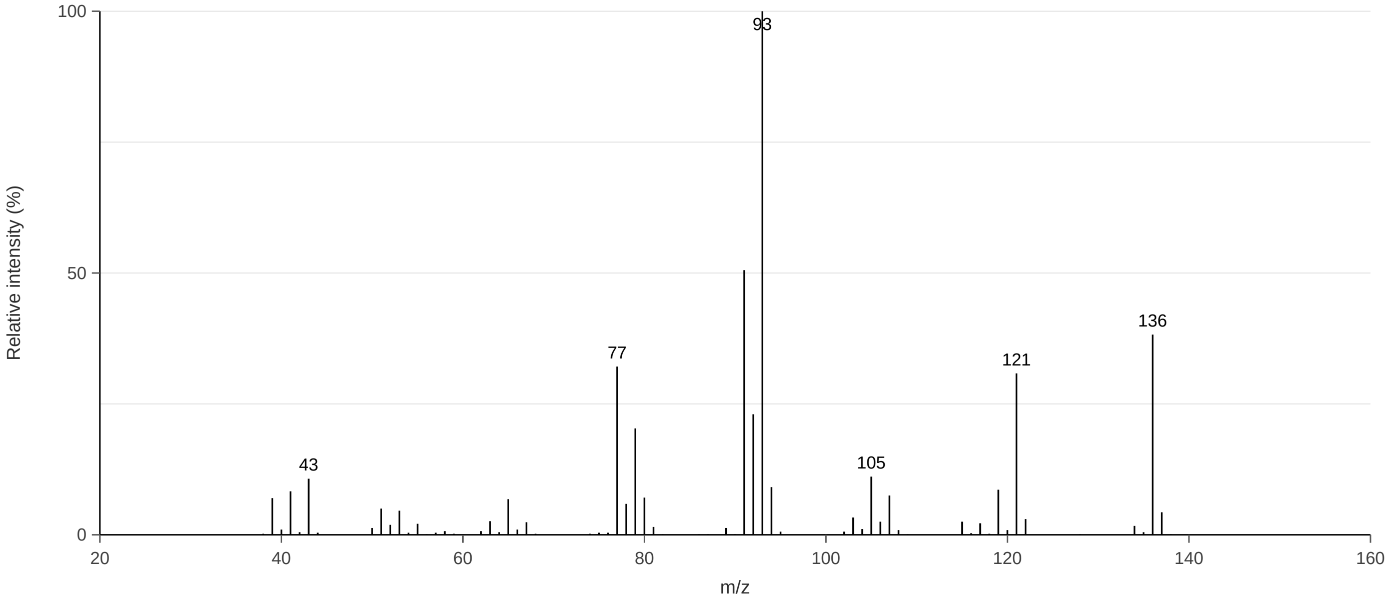

terpinolene

Experimental LRI: 1090

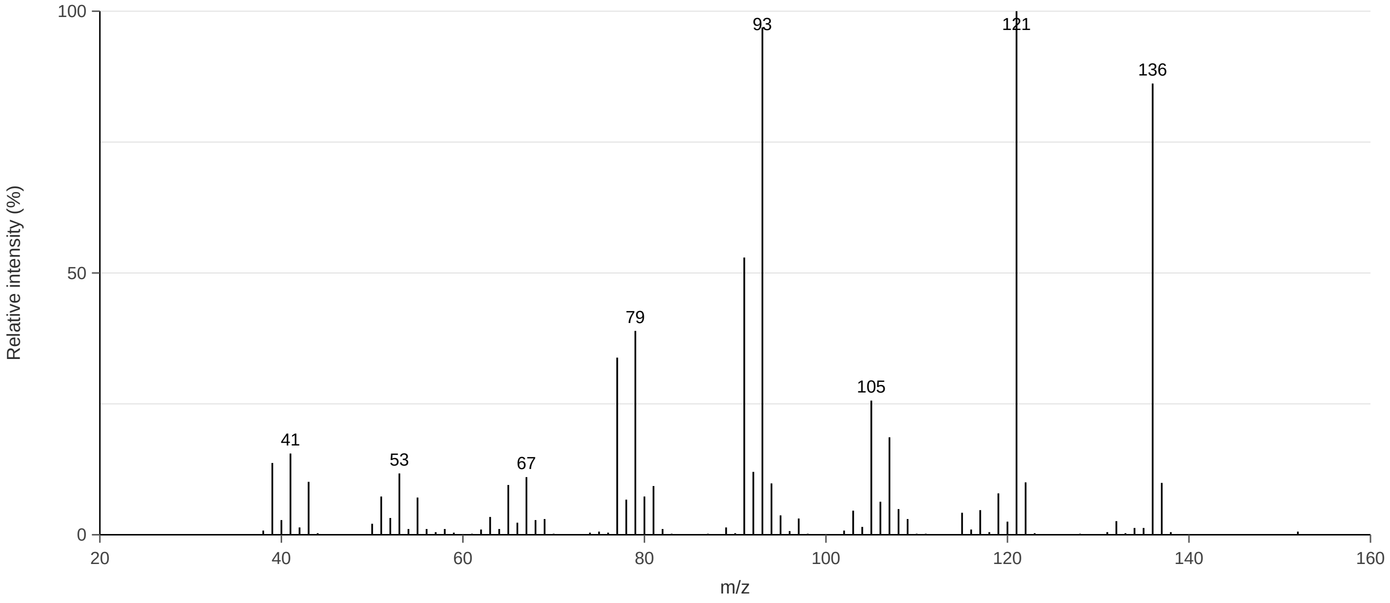

linalool

Experimental LRI: 1101

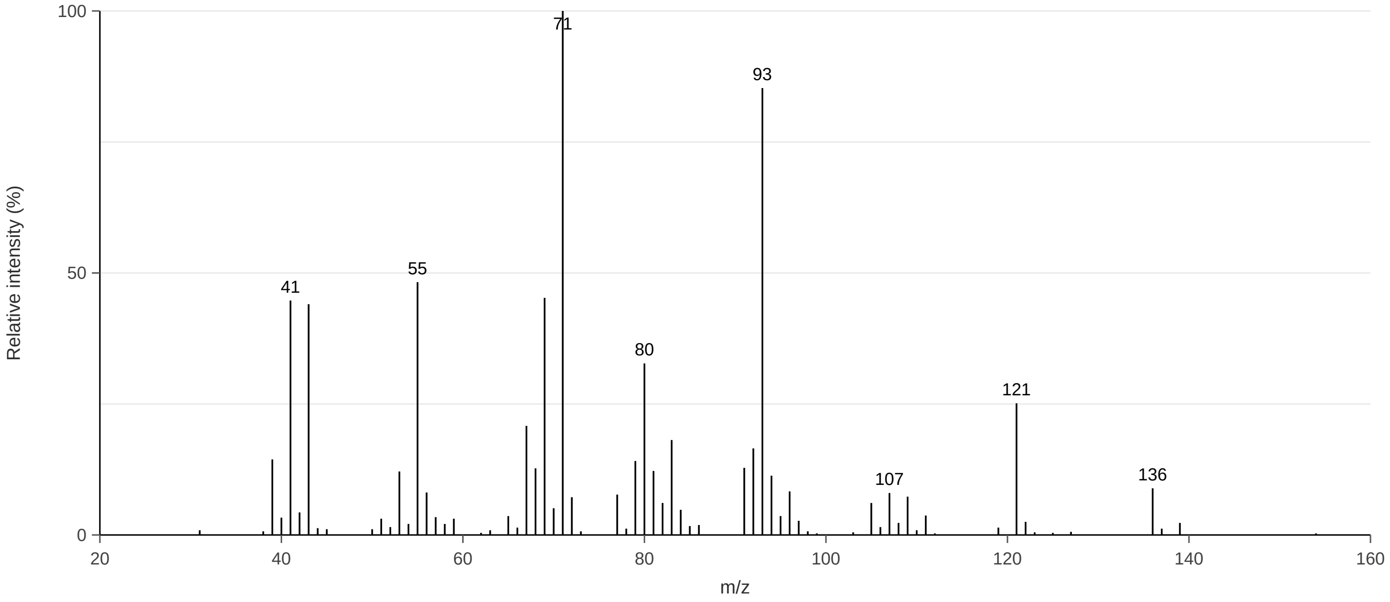

nonanal

Experimental LRI: 1102

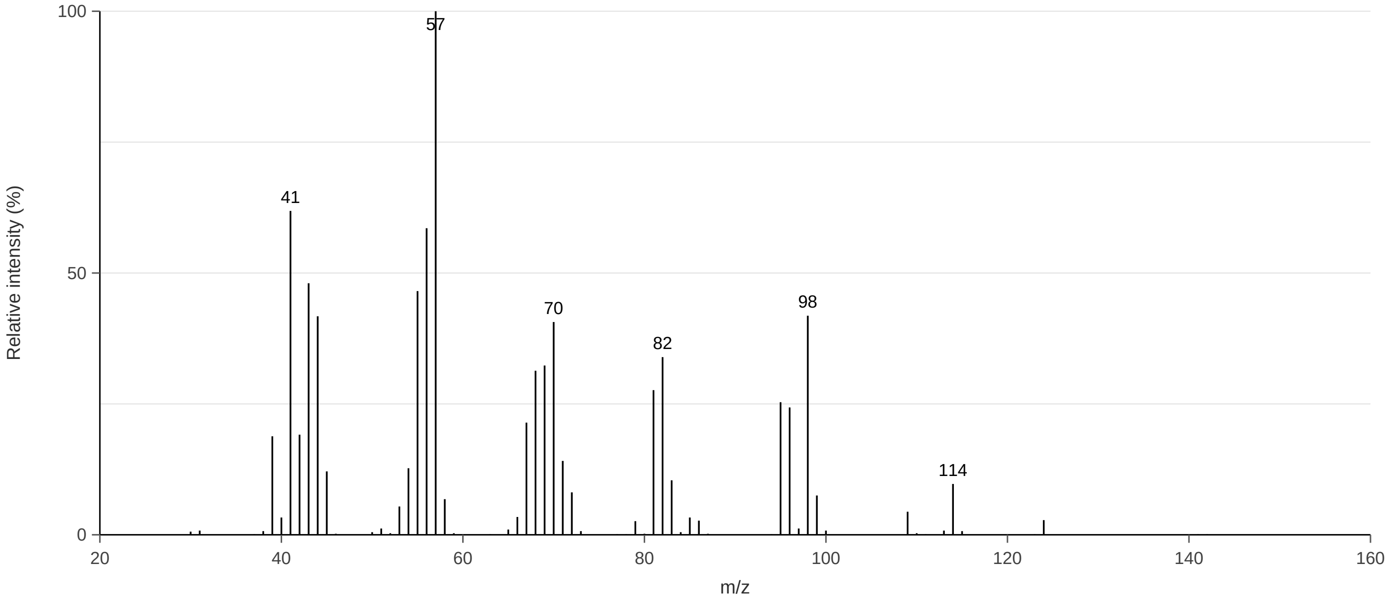

cis-p-menth-2-en-1-ol

Experimental LRI: 1123

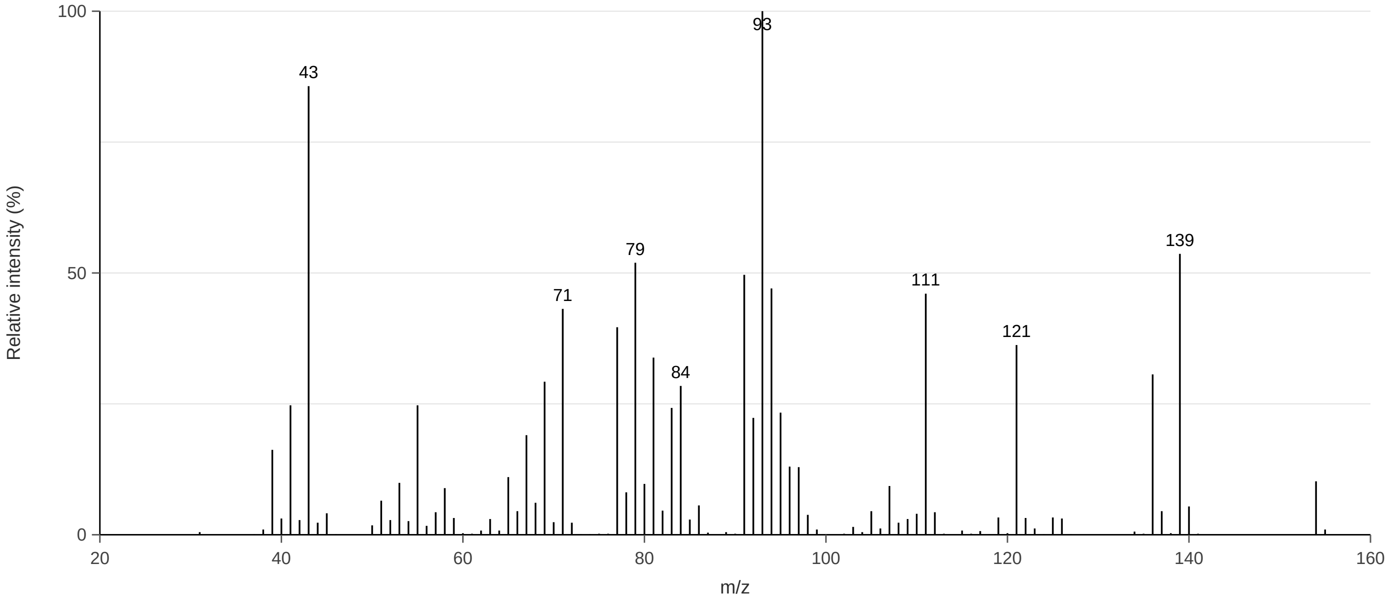

trans-pinocarveol

Experimental LRI: 1141

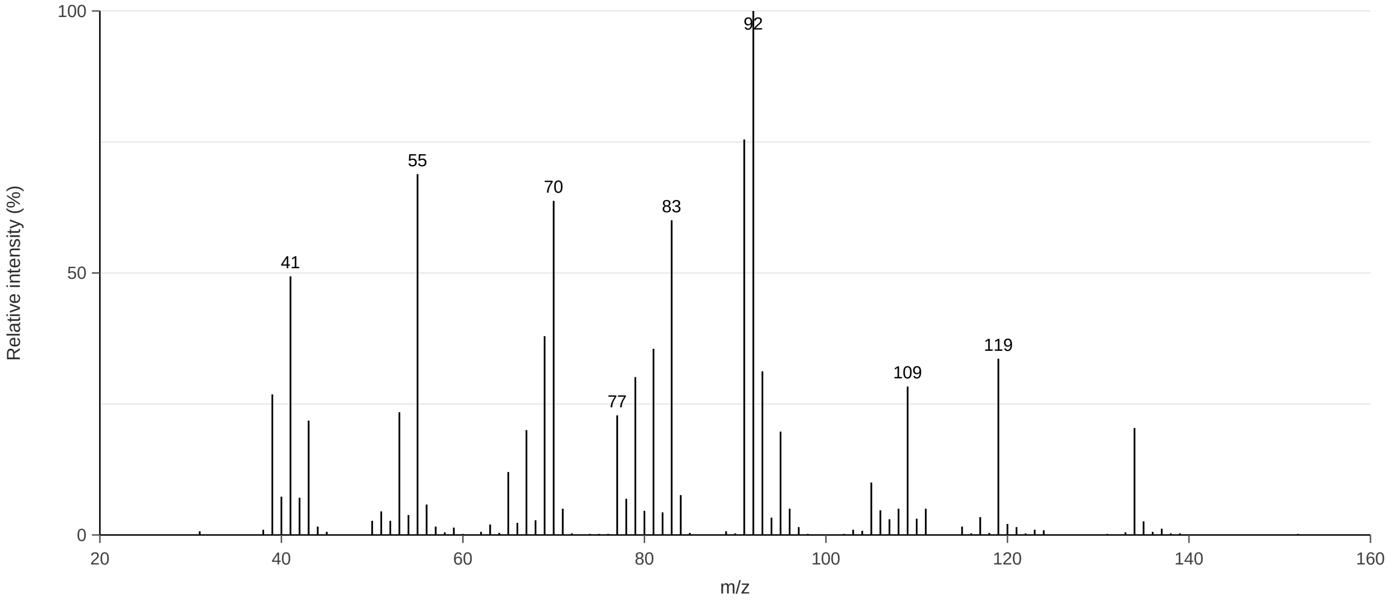

cis-verbenol

Experimental LRI: 1142

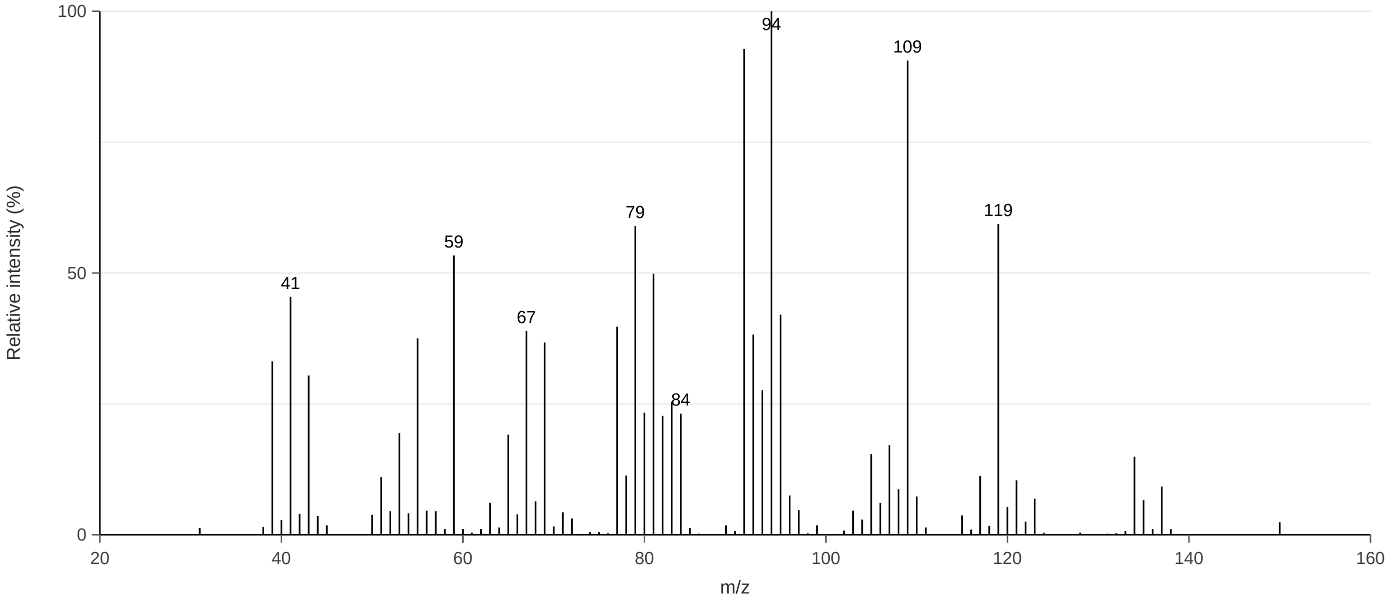

trans-p-menth-2-en-1-ol

Experimental LRI: 1142

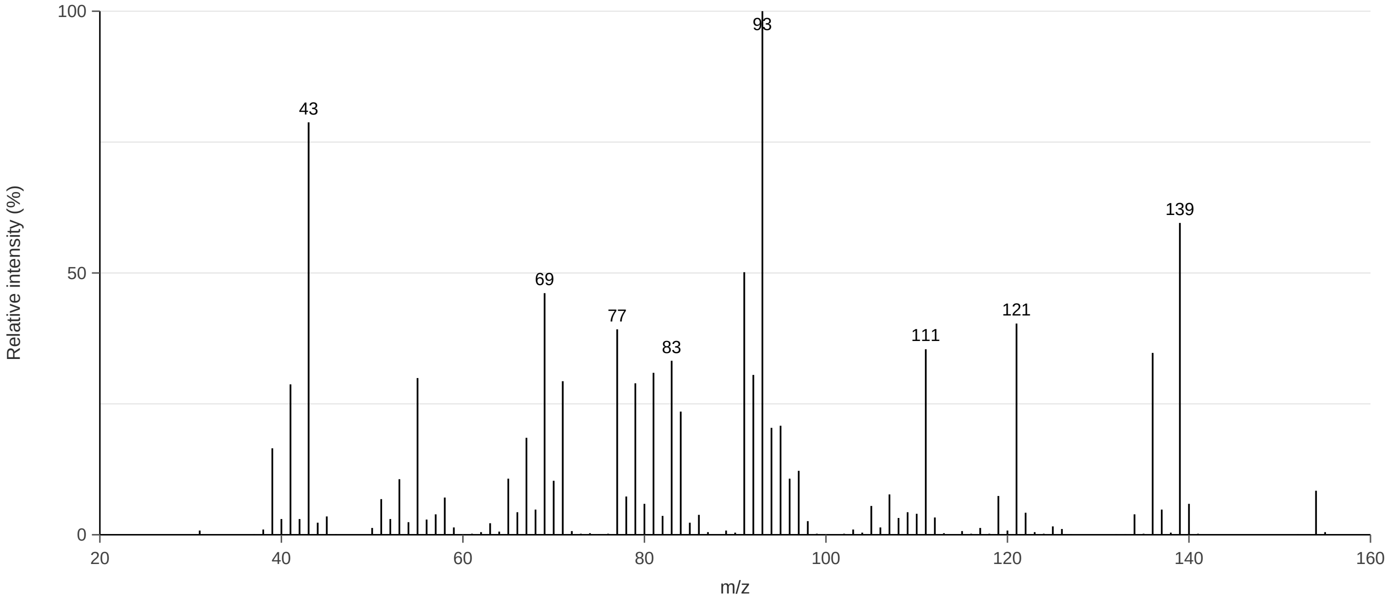

trans-verbenol

Experimental LRI: 1143

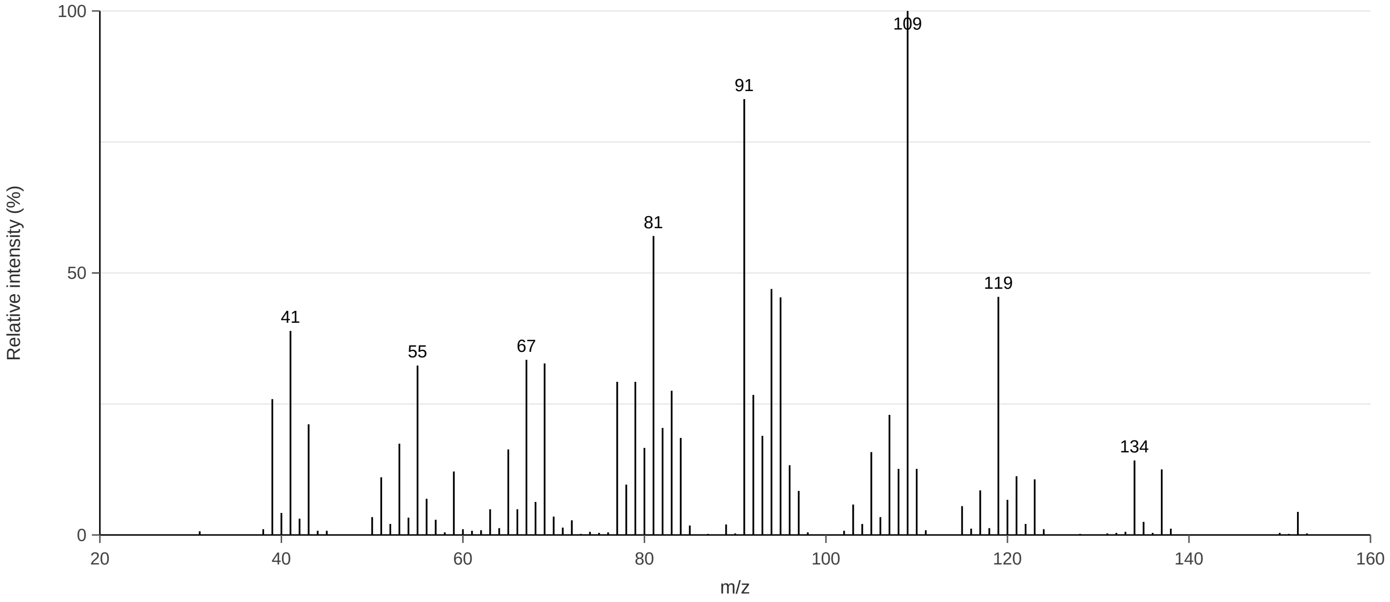

camphene hydrate

Experimental LRI: 1150

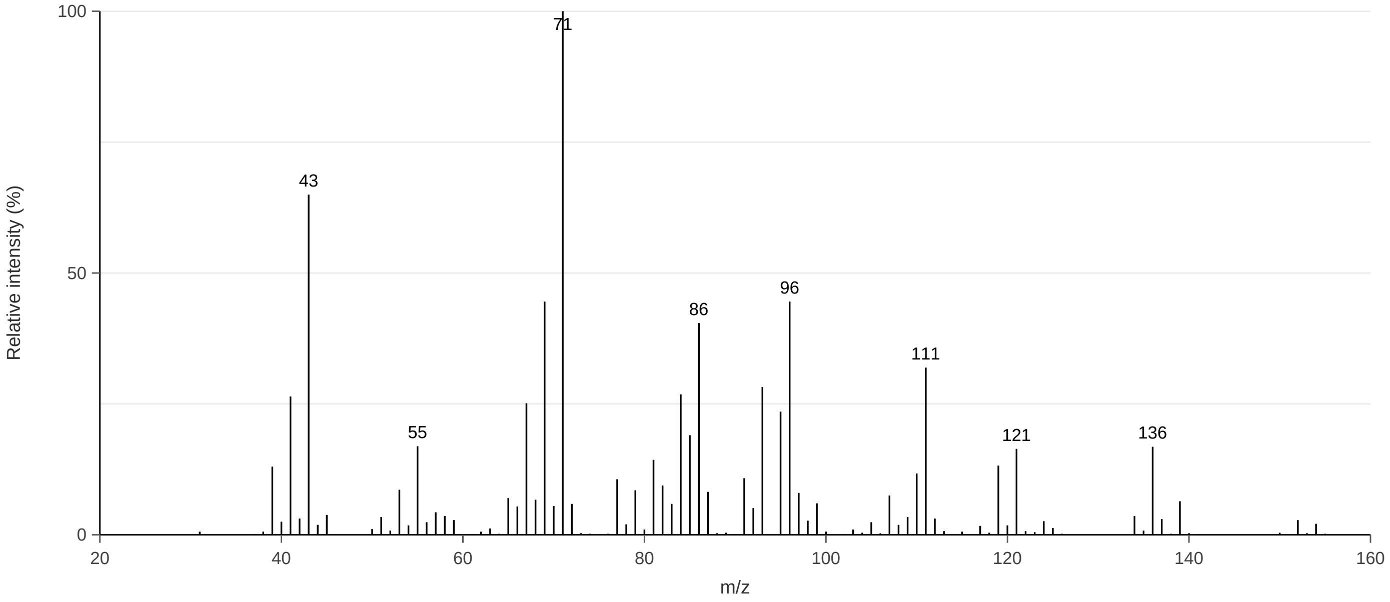

borneol

Experimental LRI: 1167

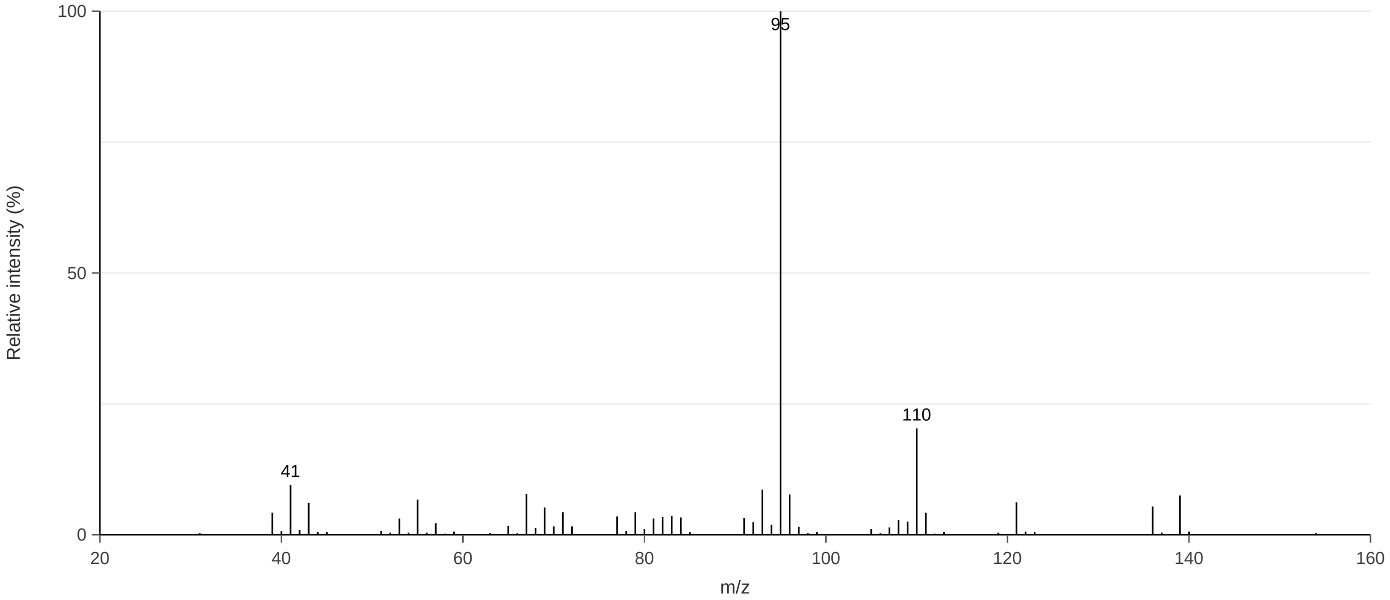

4-terpineol

Experimental LRI: 1179

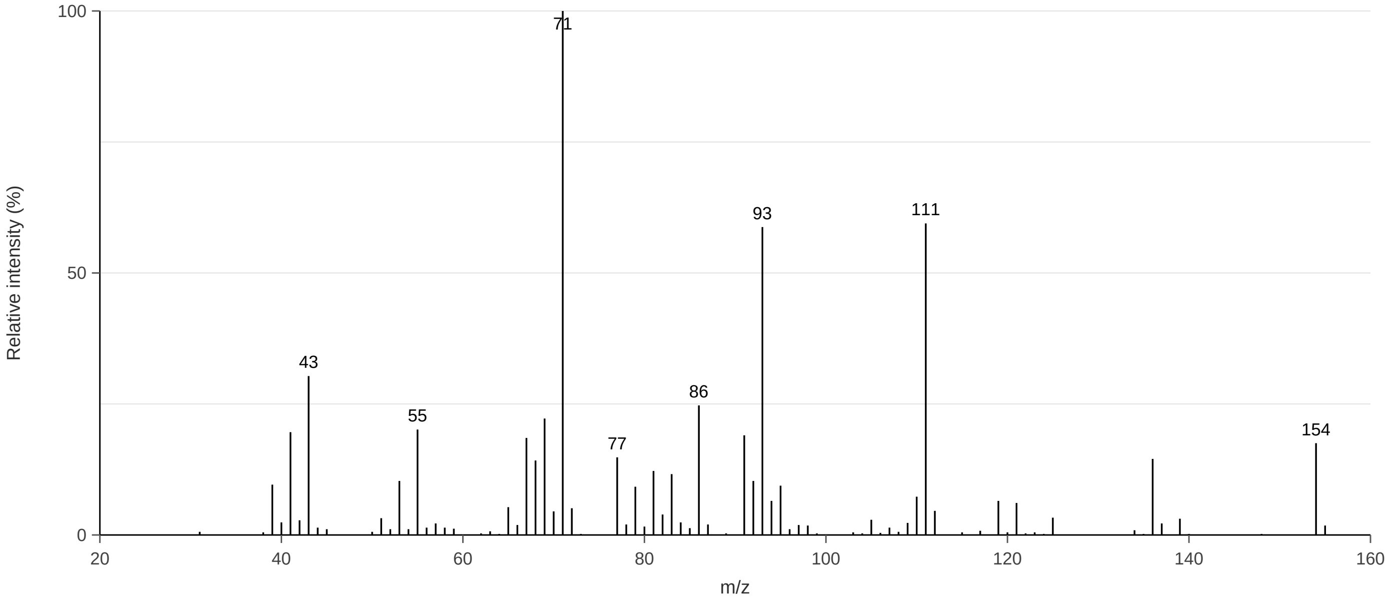

alpha-terpineol

Experimental LRI: 1191

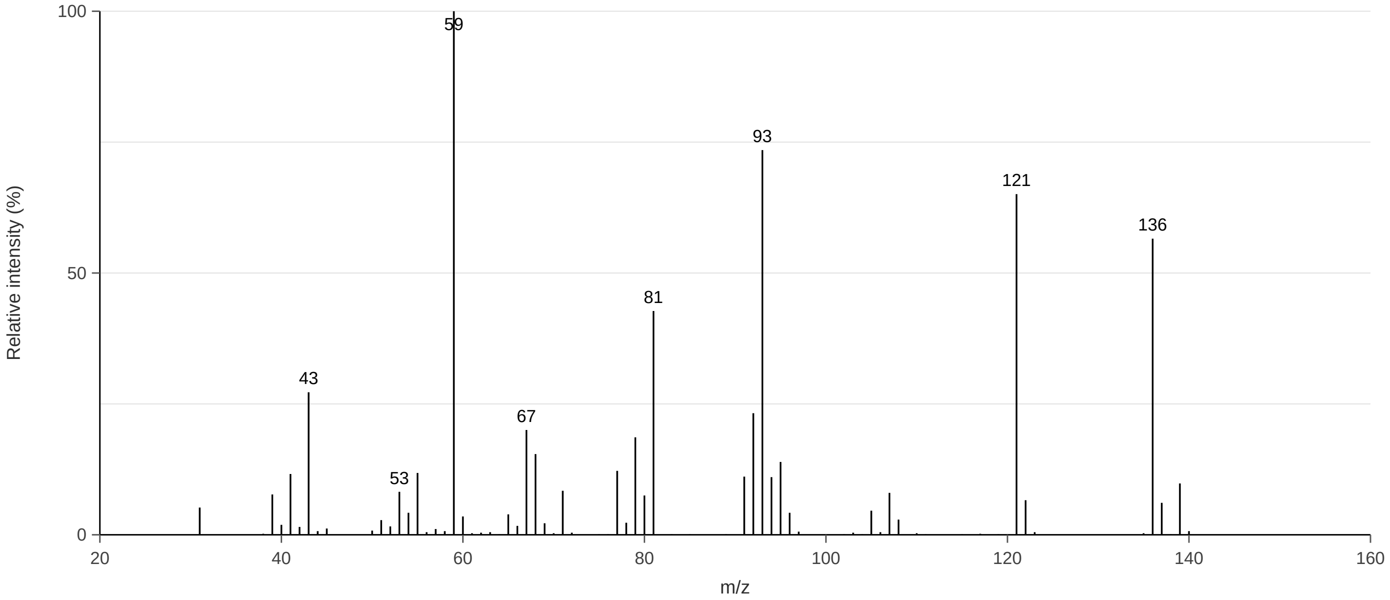

myrtenal Experimental LRI: 1194

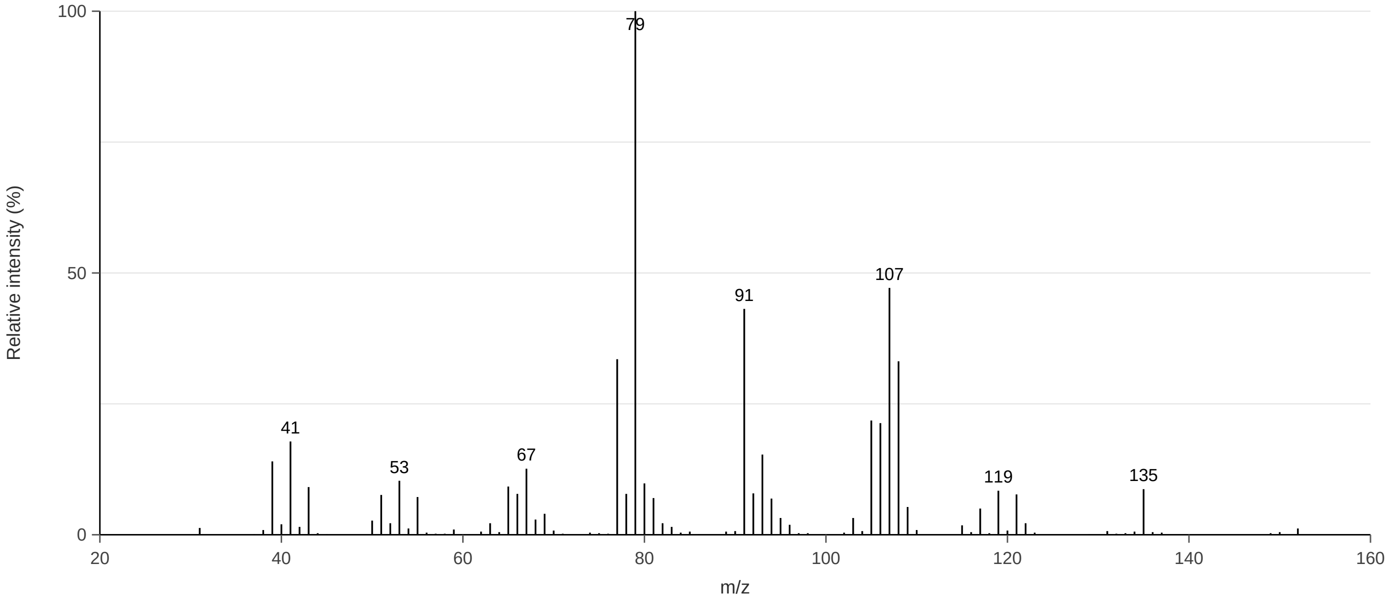

verbenone Experimental LRI: 1205

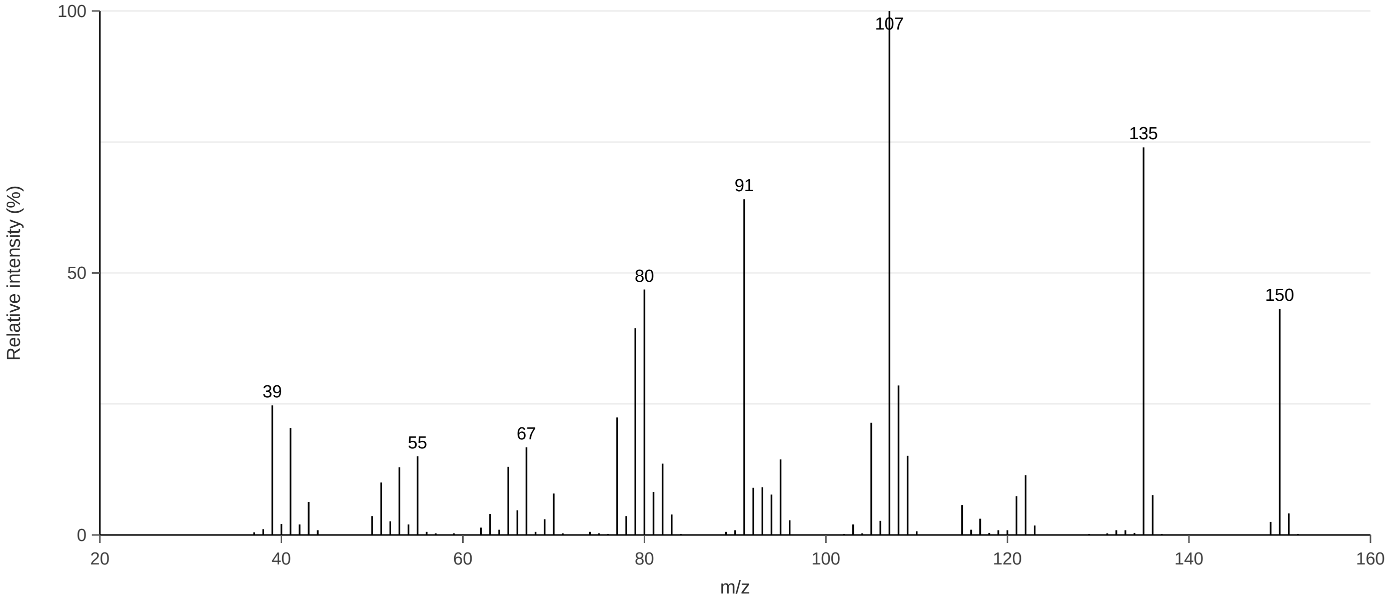

cis-carvenone oxide Experimental LRI: 1263

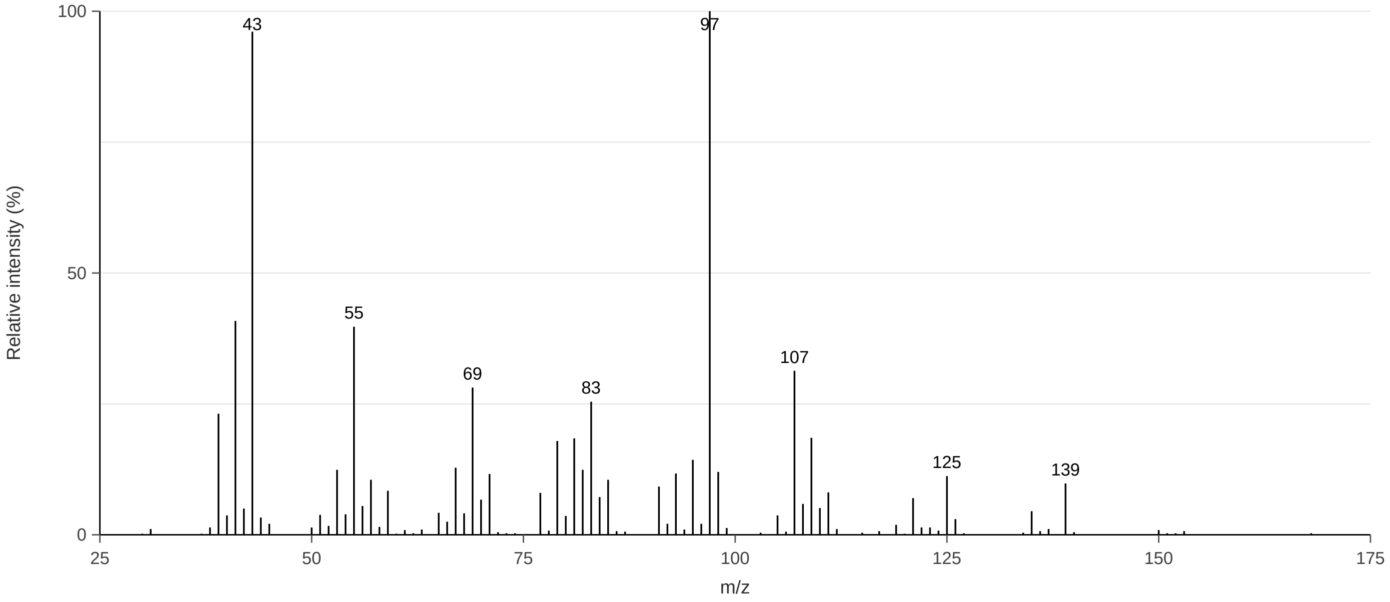

bornyl acetate

Experimental LRI: 1287

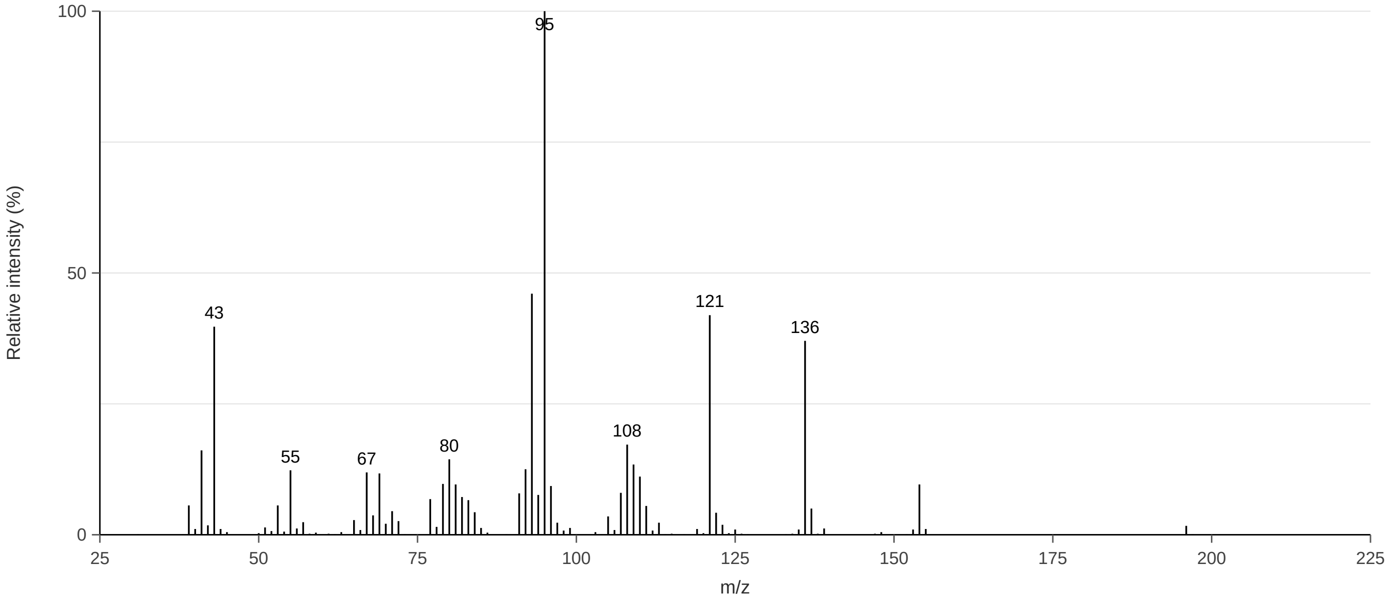

pinanediol

Experimental LRI: 1317

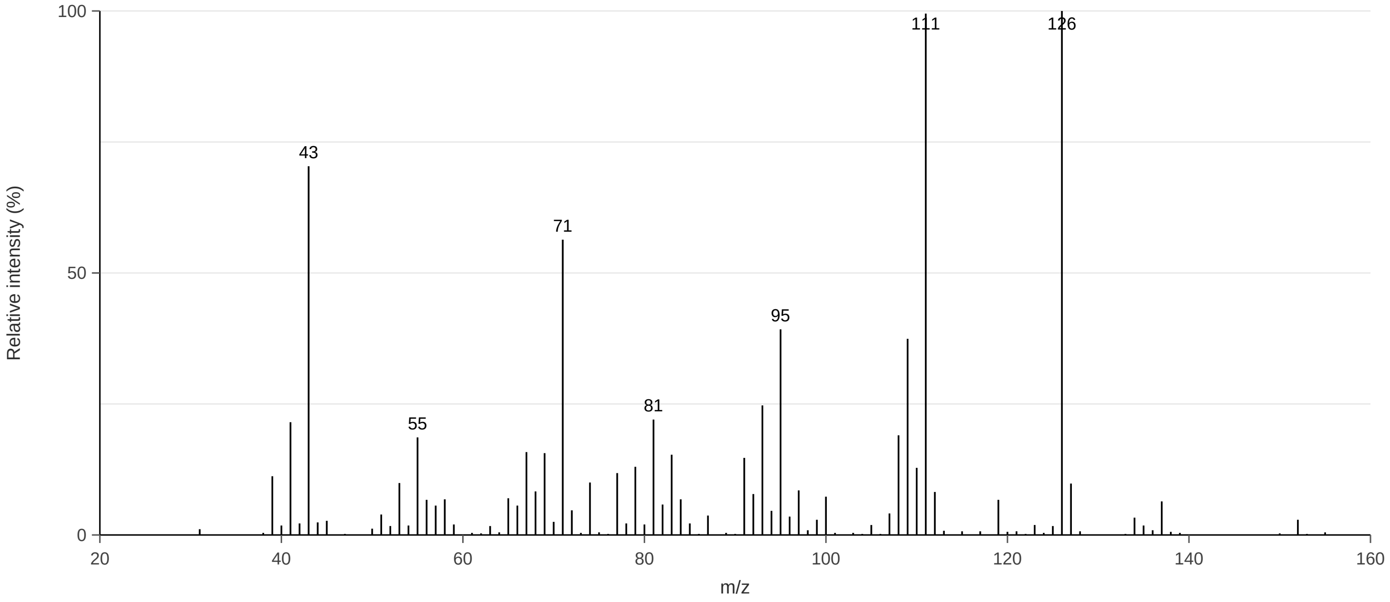

delta-elemene

Experimental LRI: 1338

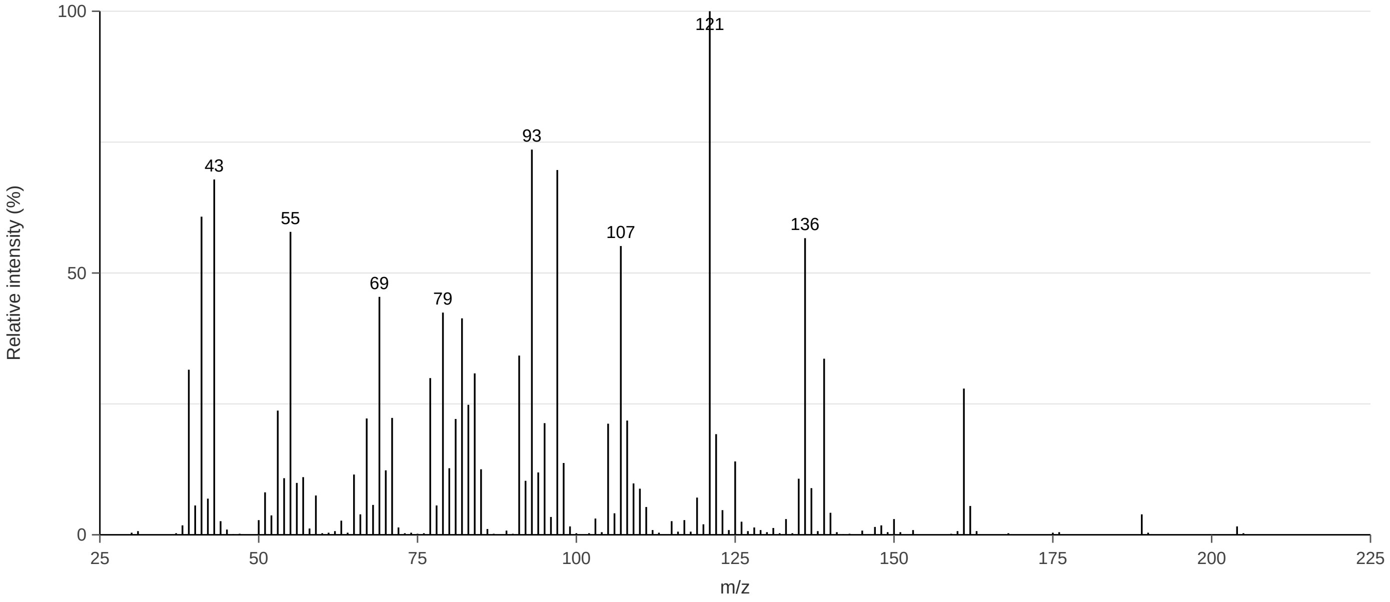

beta-elemene

Experimental LRI: 1392

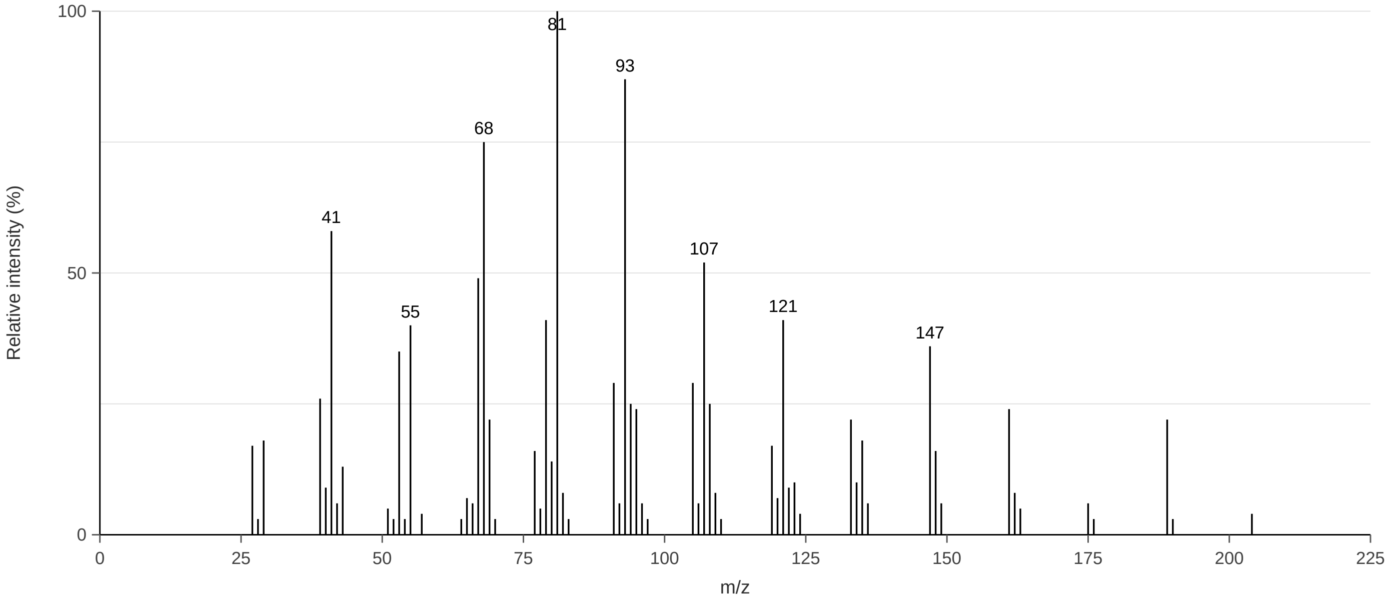

beta-caryophyllene

Experimental LRI: 1419

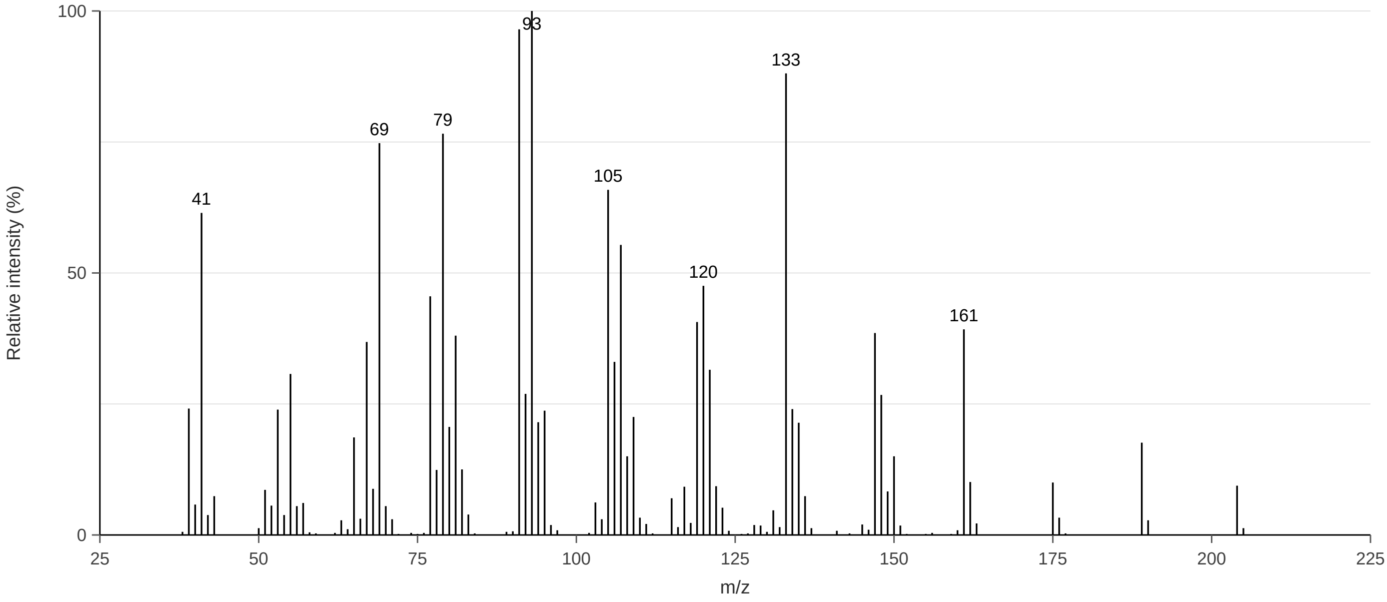

alpha-humulene

Experimental LRI: 1455

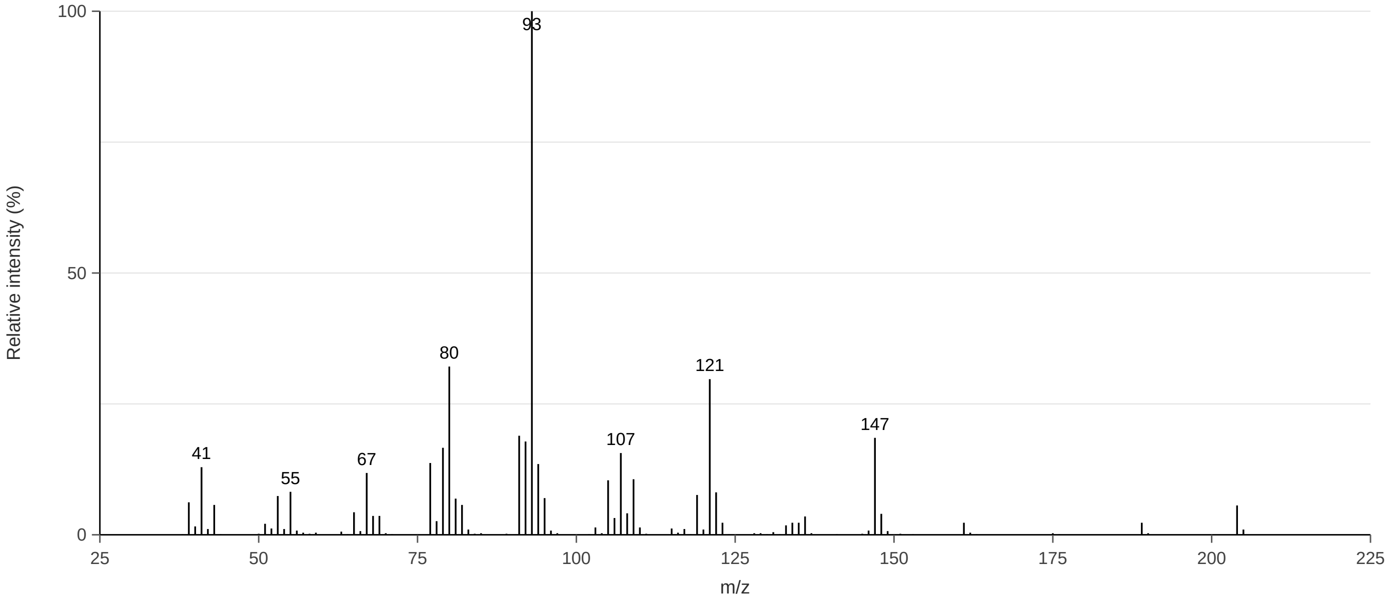

alloaromadendrene

Experimental LRI: 1462

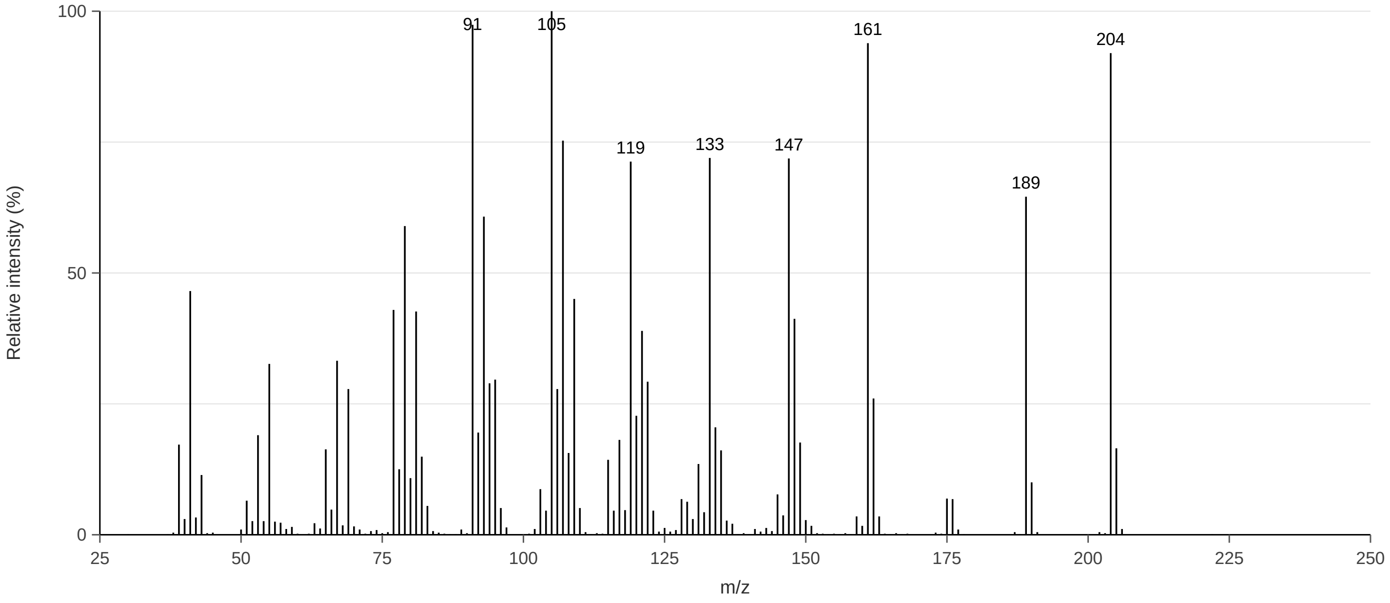

gamma-muurolene

Experimental LRI: 1478

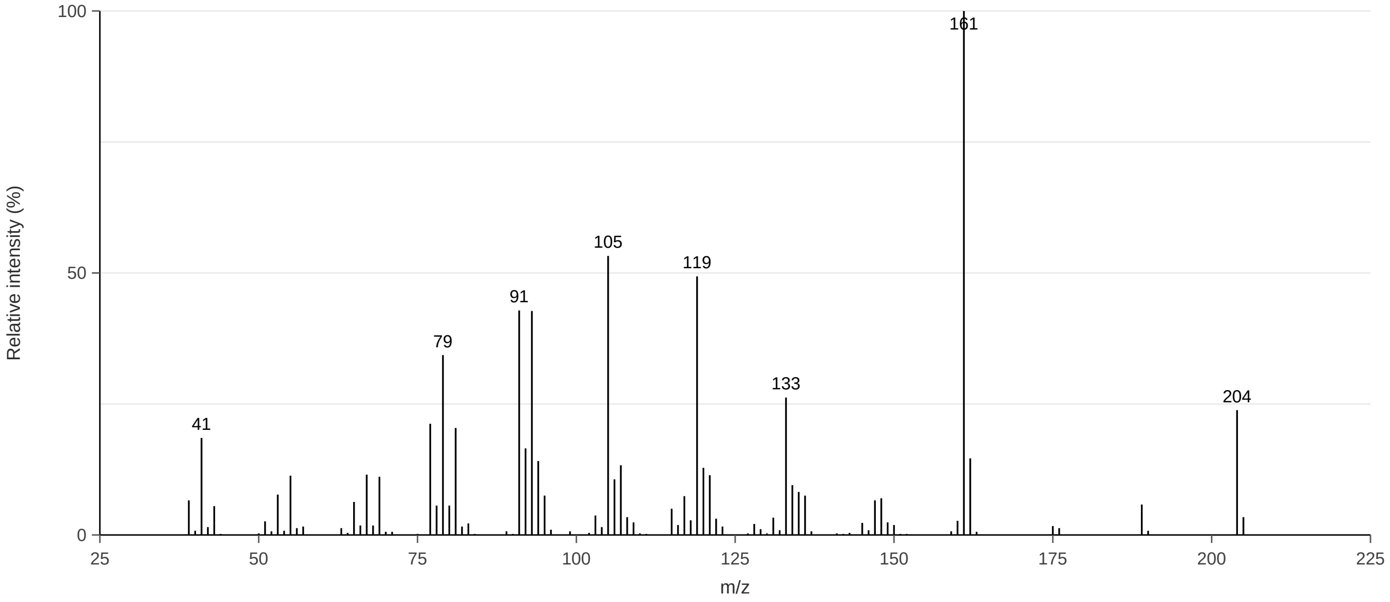

germacrene D

Experimental LRI: 1482

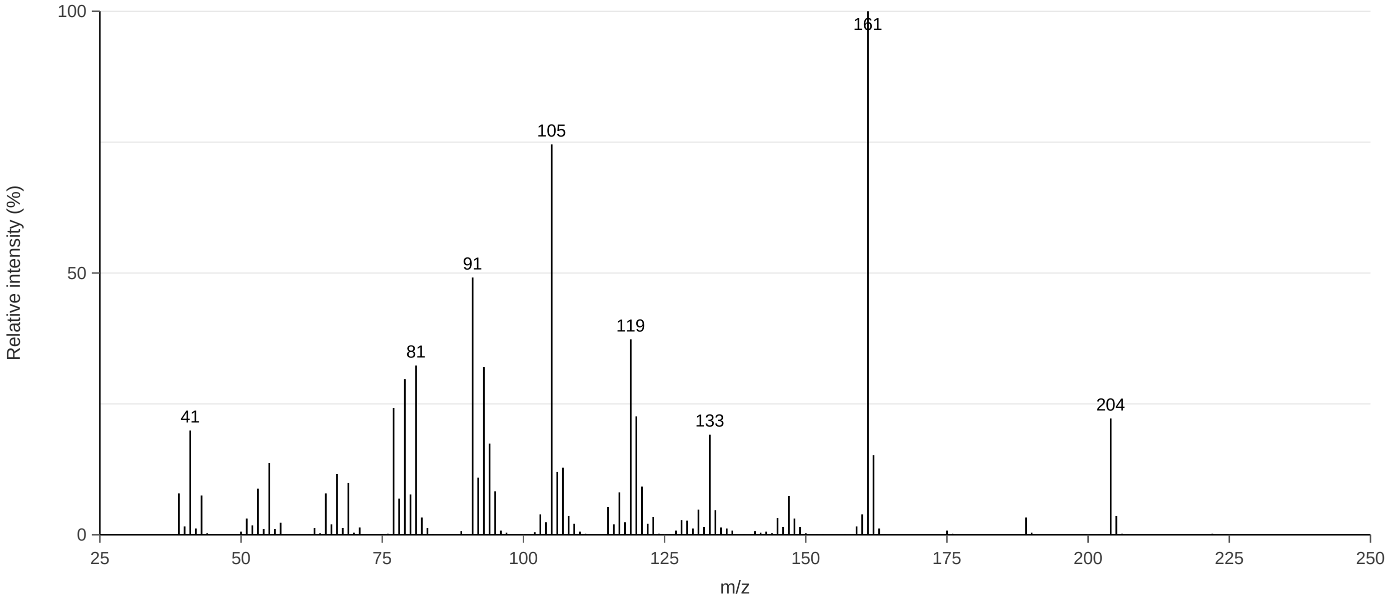

beta-selinene Experimental LRI: 1487

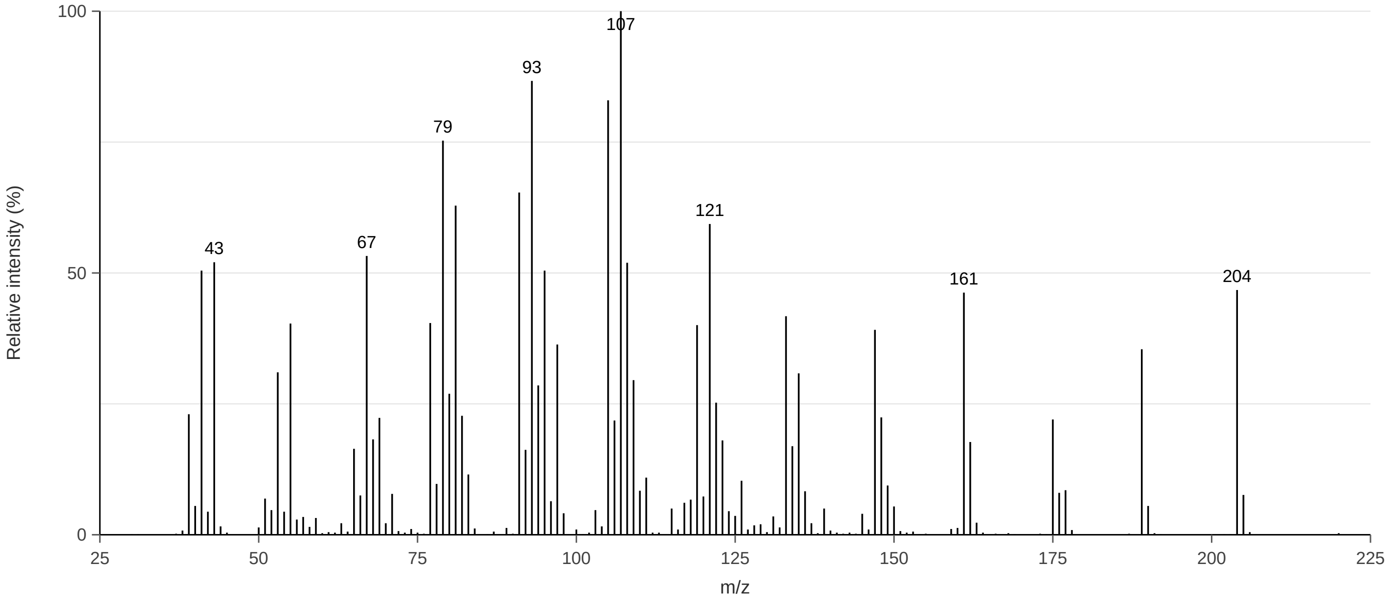

viridiflorene Experimental LRI: 1494

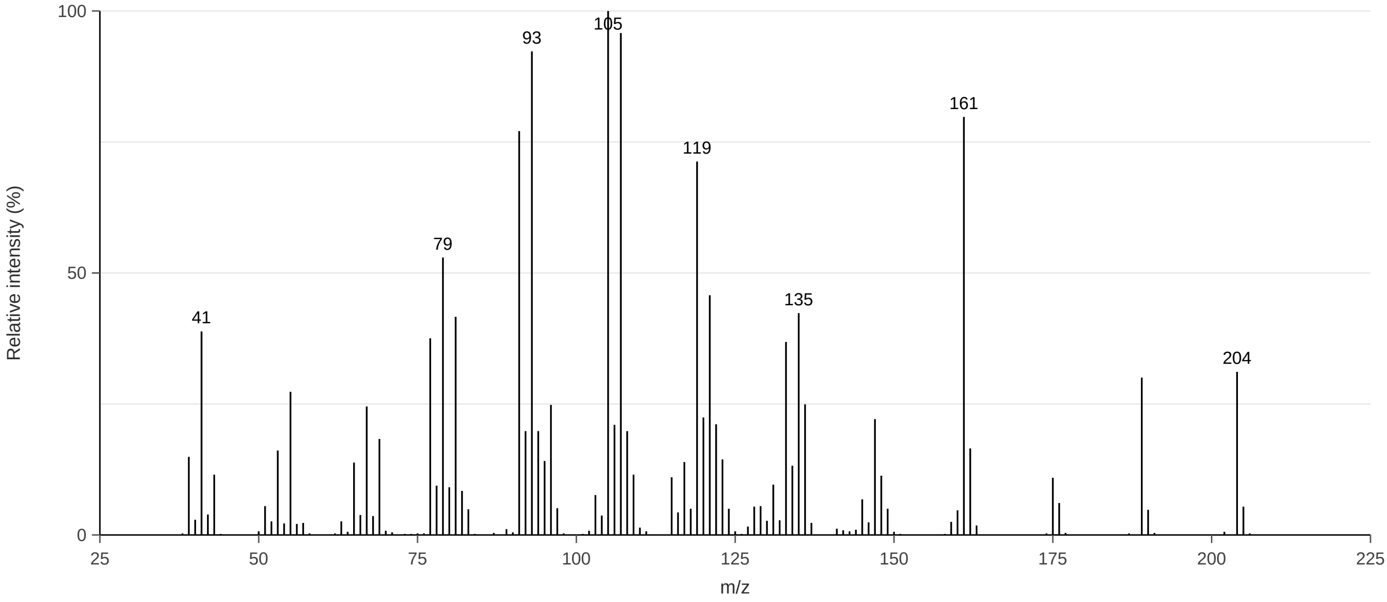

bicyclogermacrene Experimental LRI: 1496

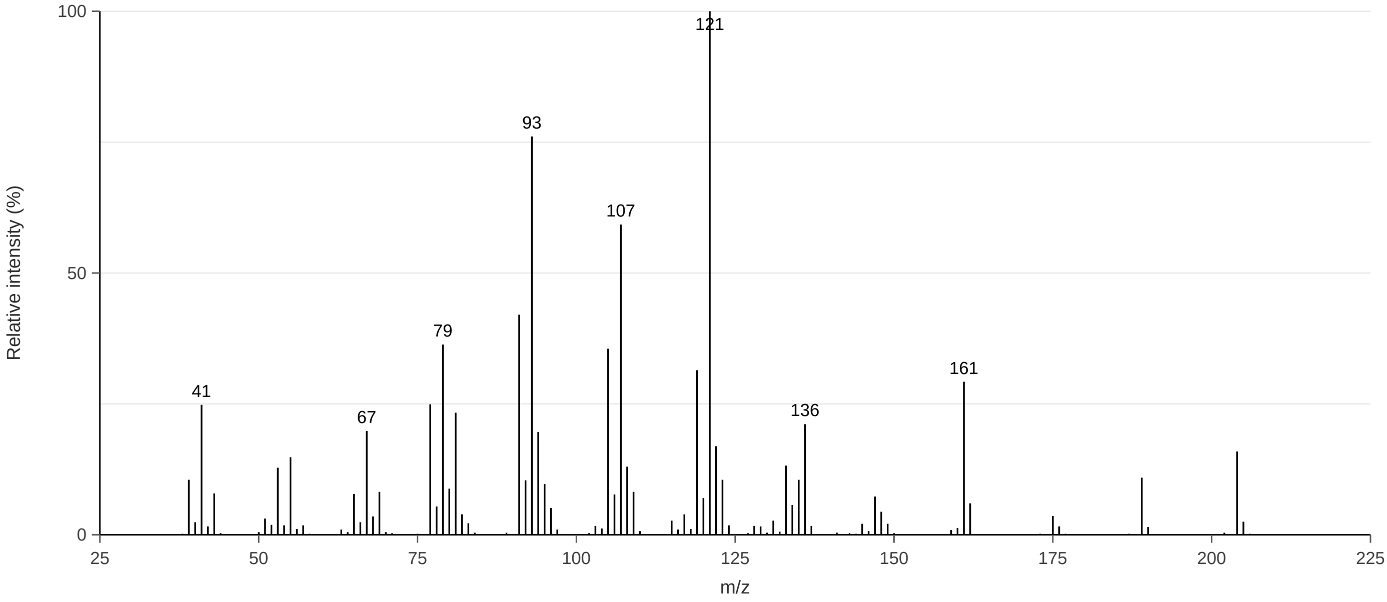

cuparene

Experimental LRI: 1505

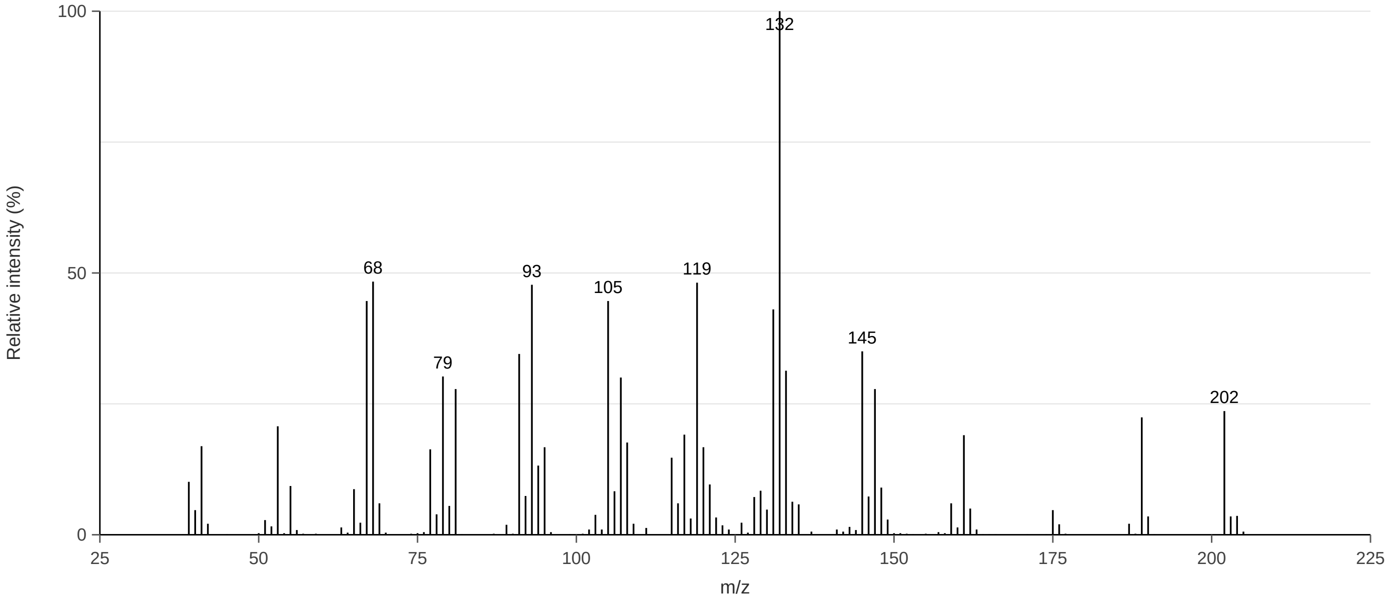

delta-cadinene

Experimental LRI: 1524

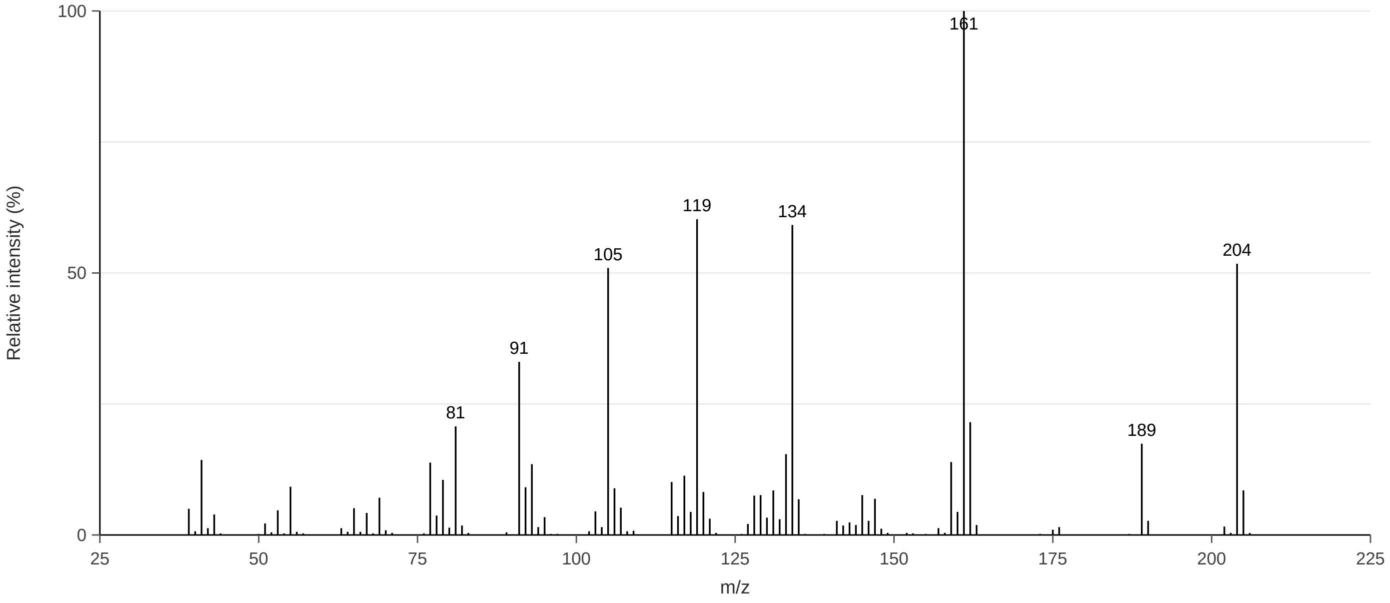

germacrene B

Experimental LRI: 1557

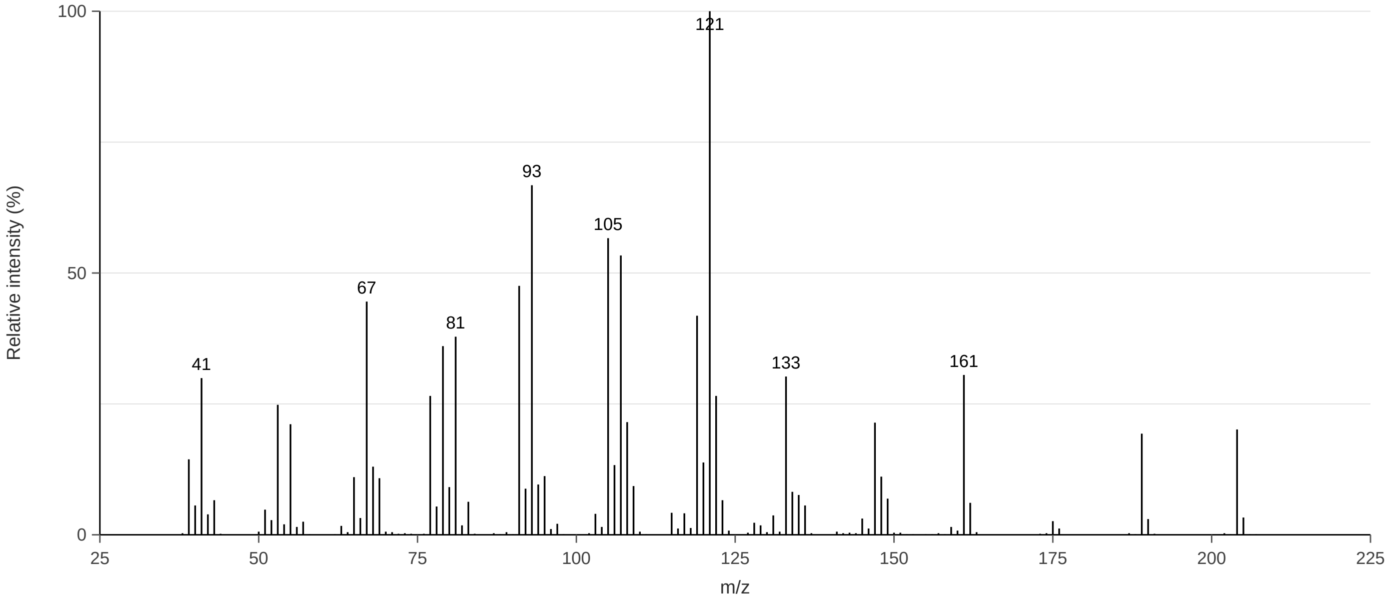

(E)-nerolidol

Experimental LRI: 1564

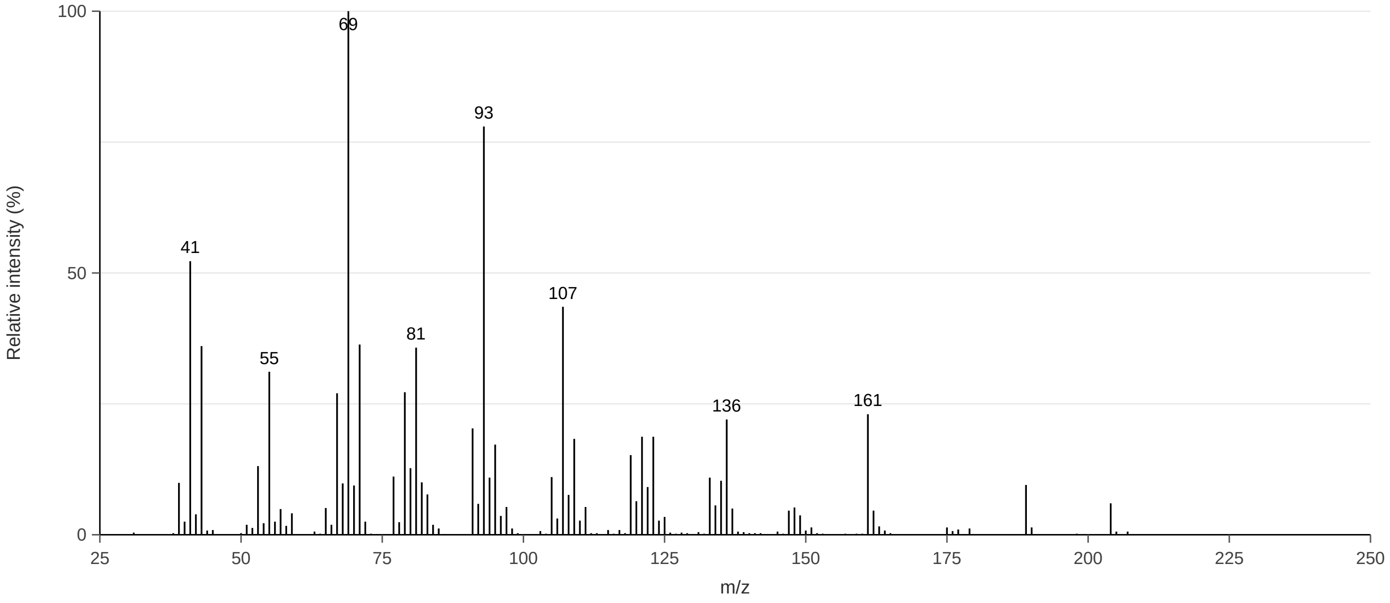

ledol

Experimental LRI: 1565

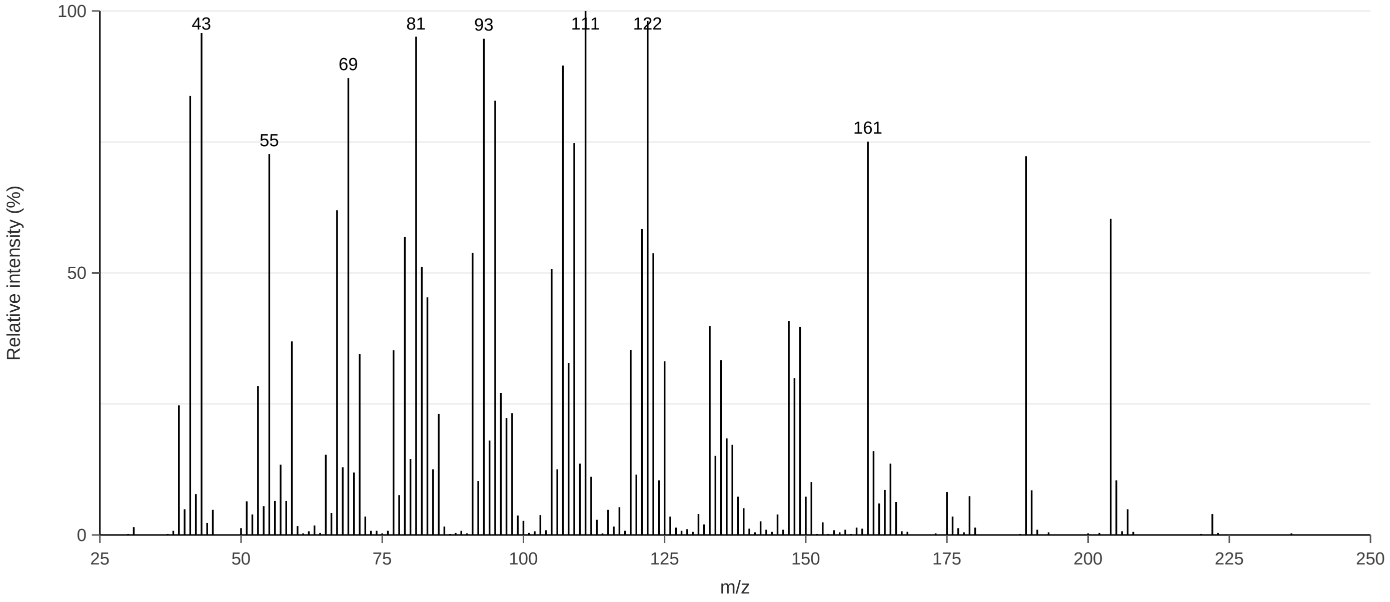

spathulenol

Experimental LRI: 1576

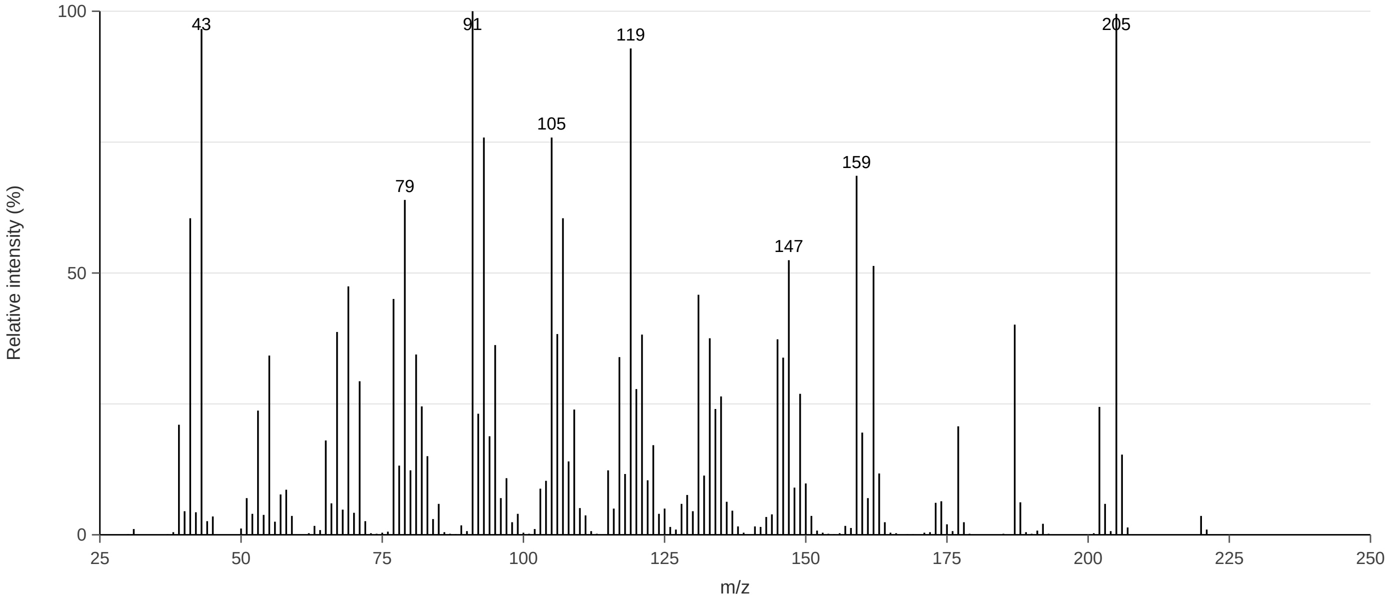

globulol

Experimental LRI: 1584

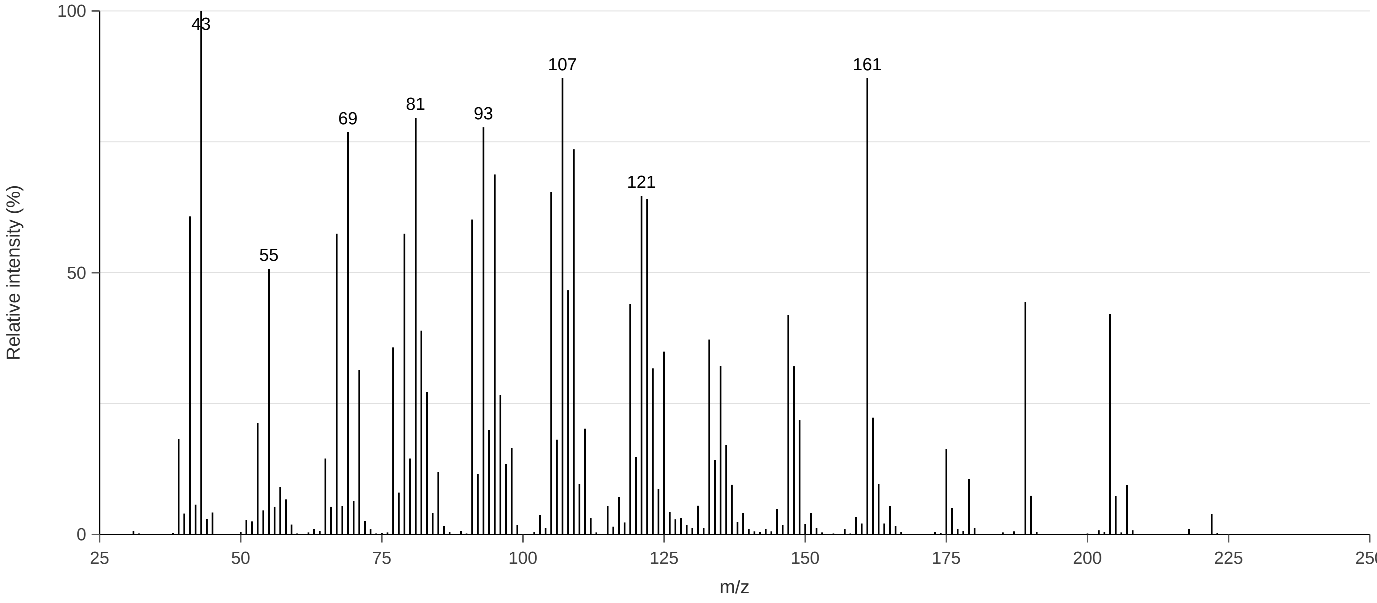

viridiflorol

Experimental LRI: 1591

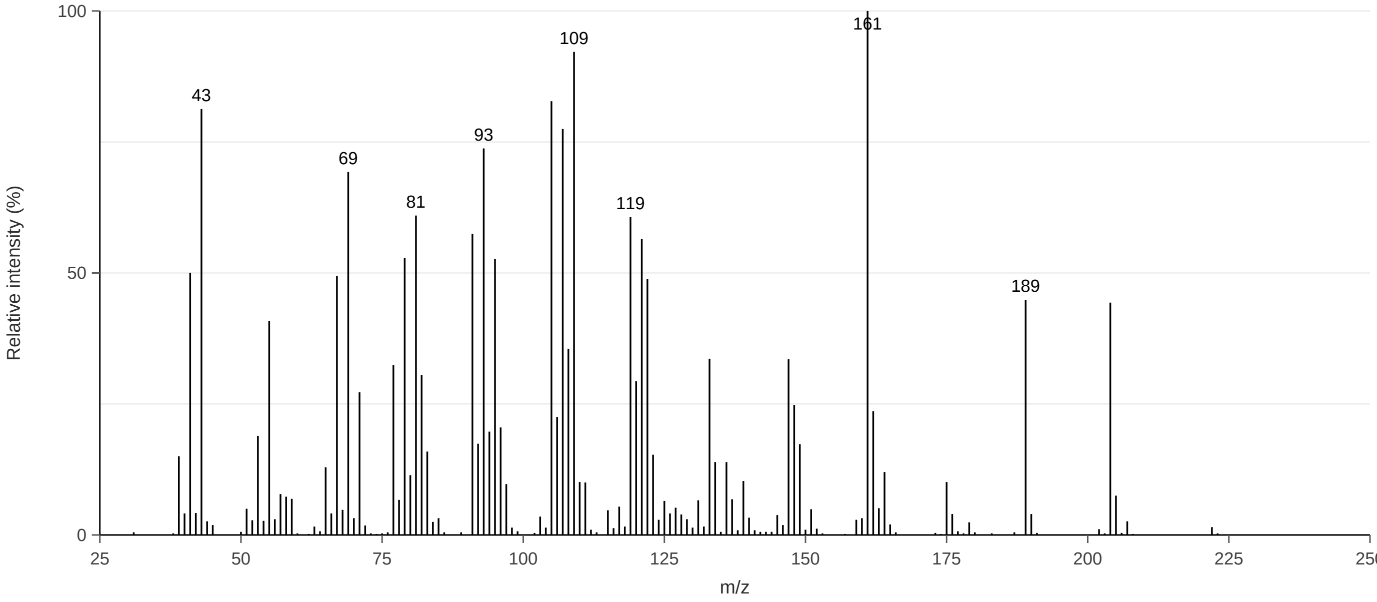

rosifoliol

Experimental LRI: 1601

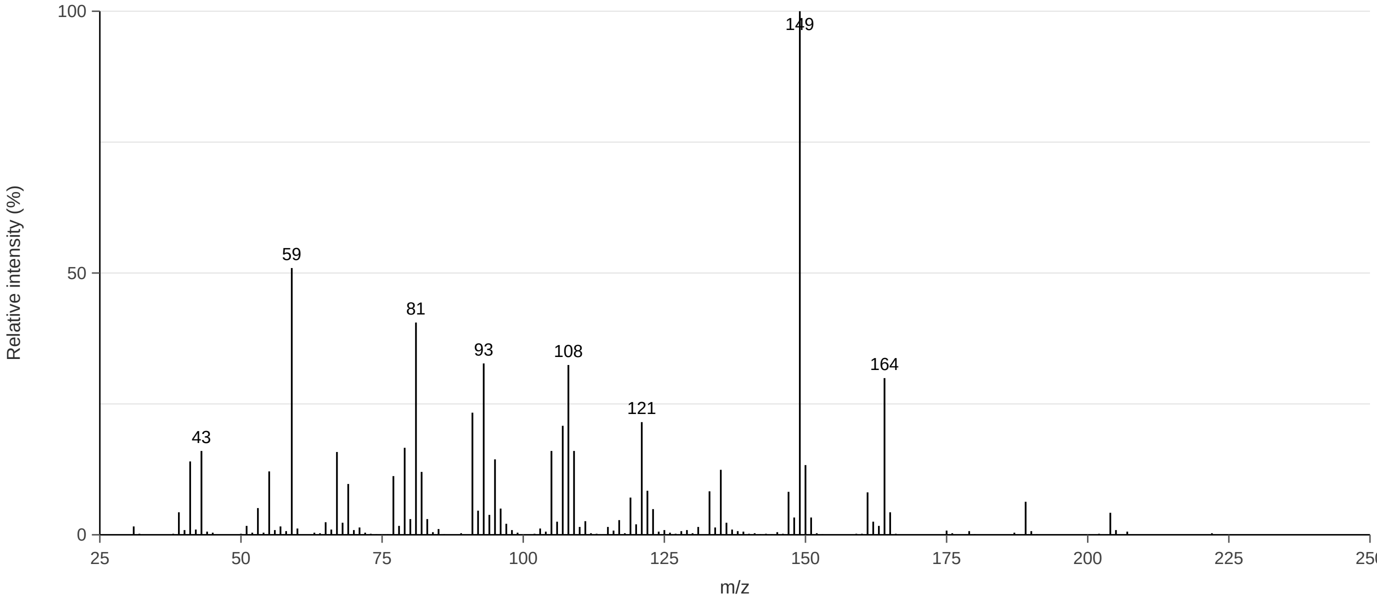

1-epi-cubenol

Experimental LRI: 1629

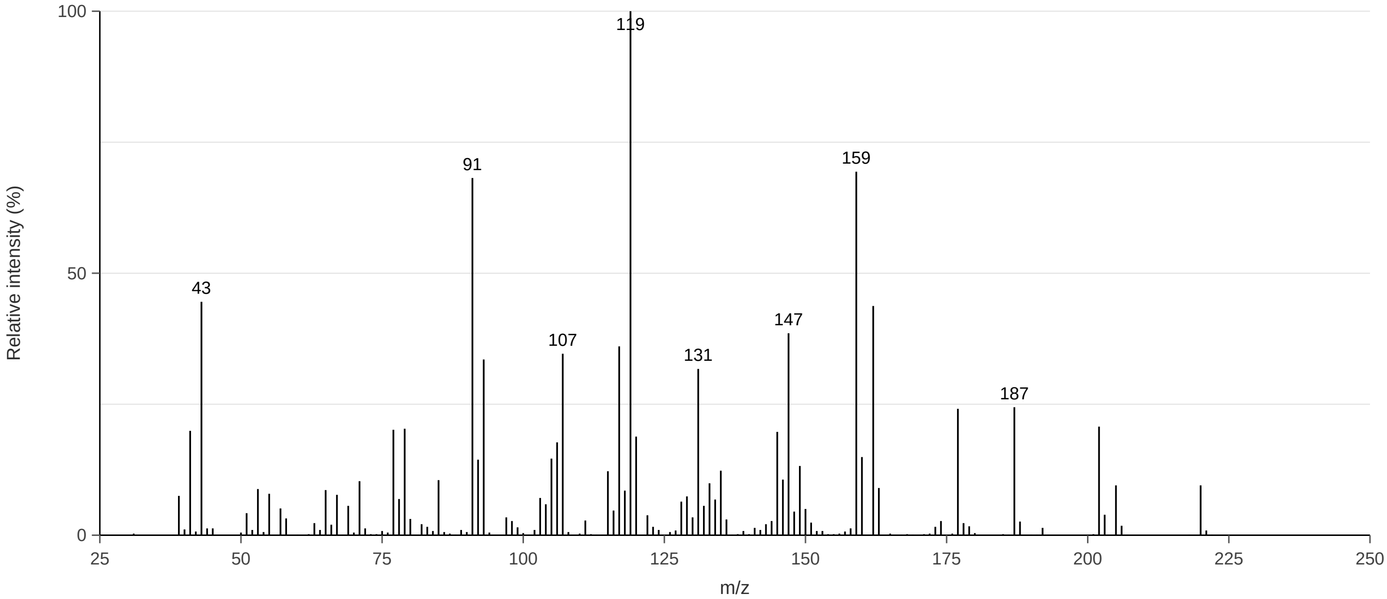

gamma-eudesmol

Experimental LRI: 1630

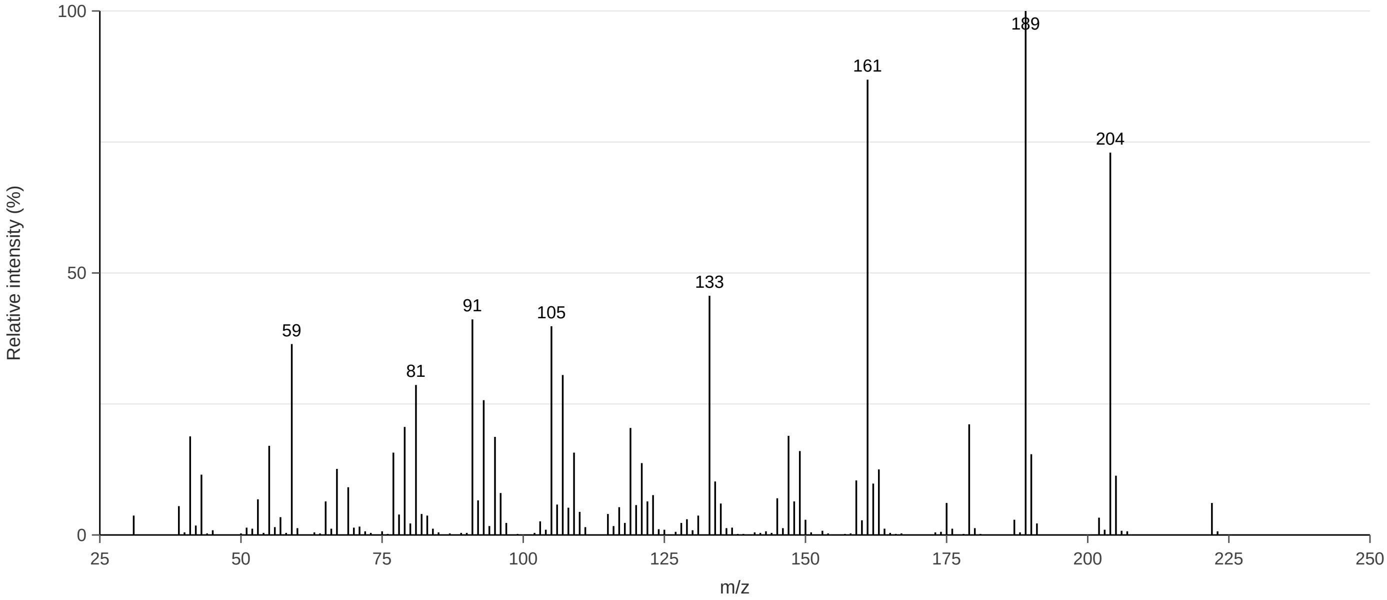

T-cadinol

Experimental LRI: 1641

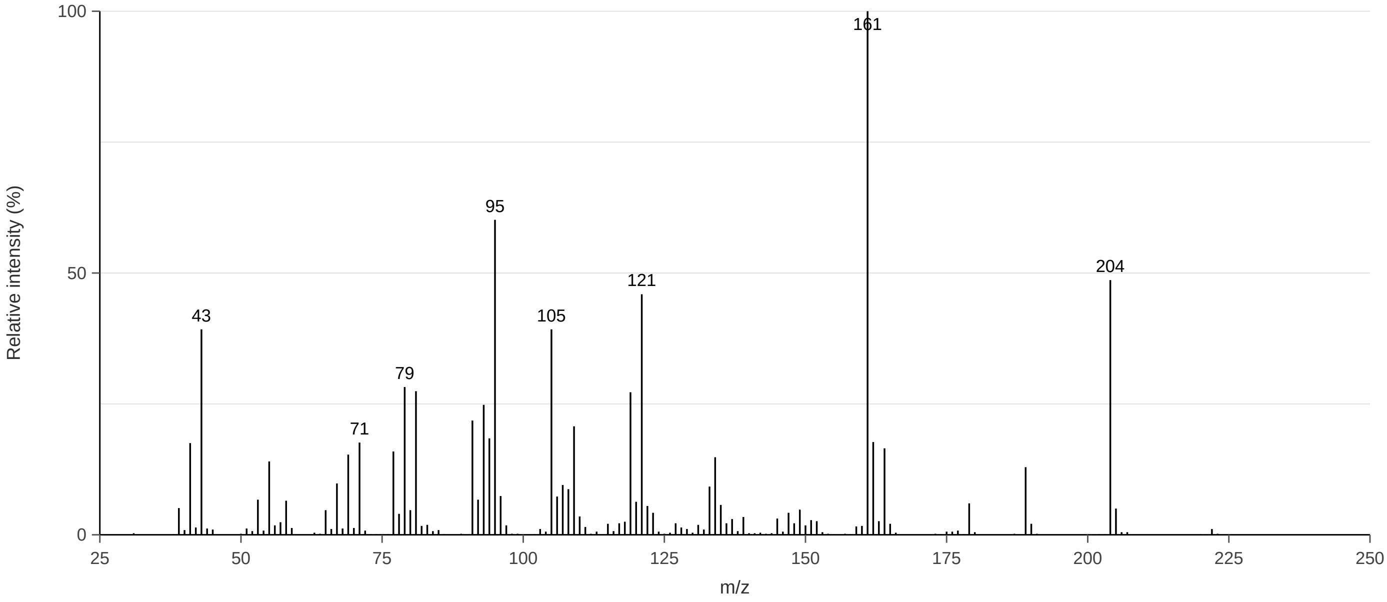

T-muurolol

Experimental LRI: 1642

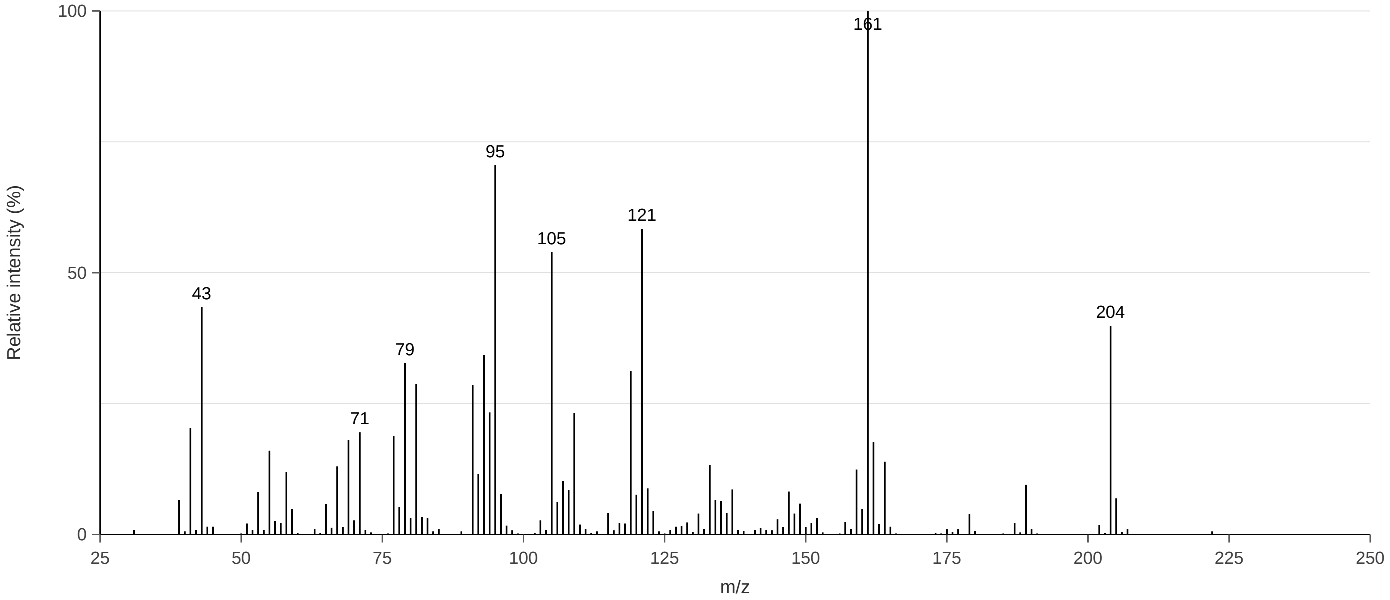

beta-eudesmol

Experimental LRI: 1650

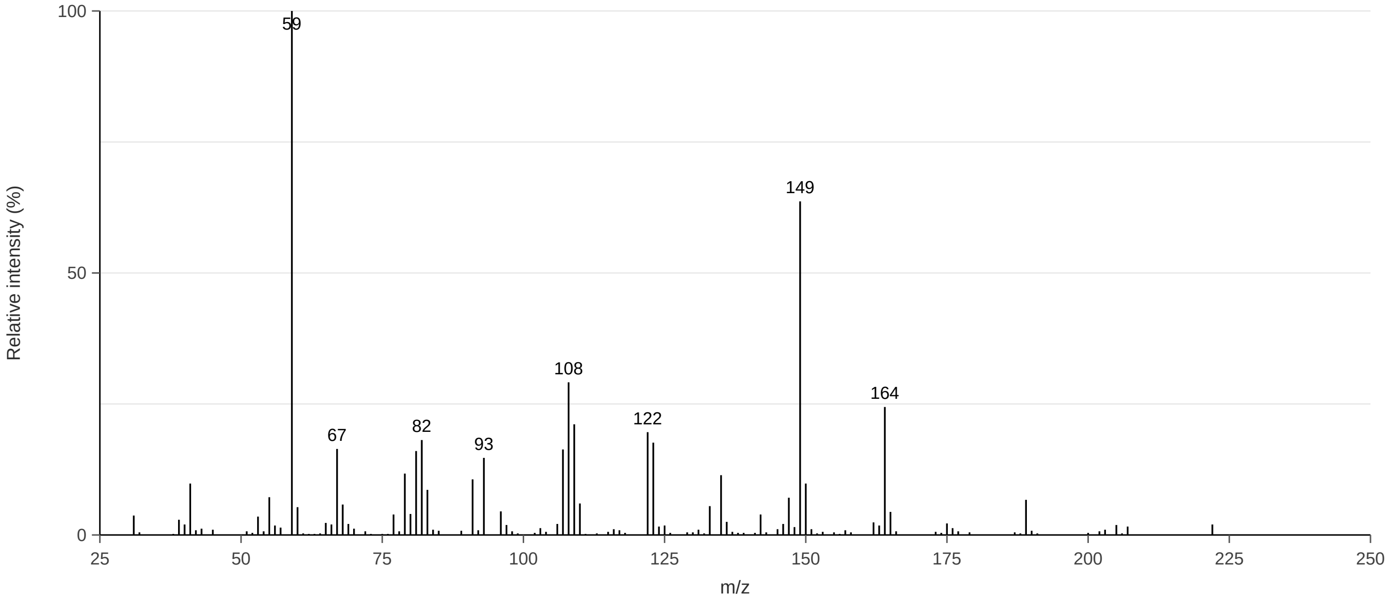

alpha-eudesmol

Experimental LRI: 1652

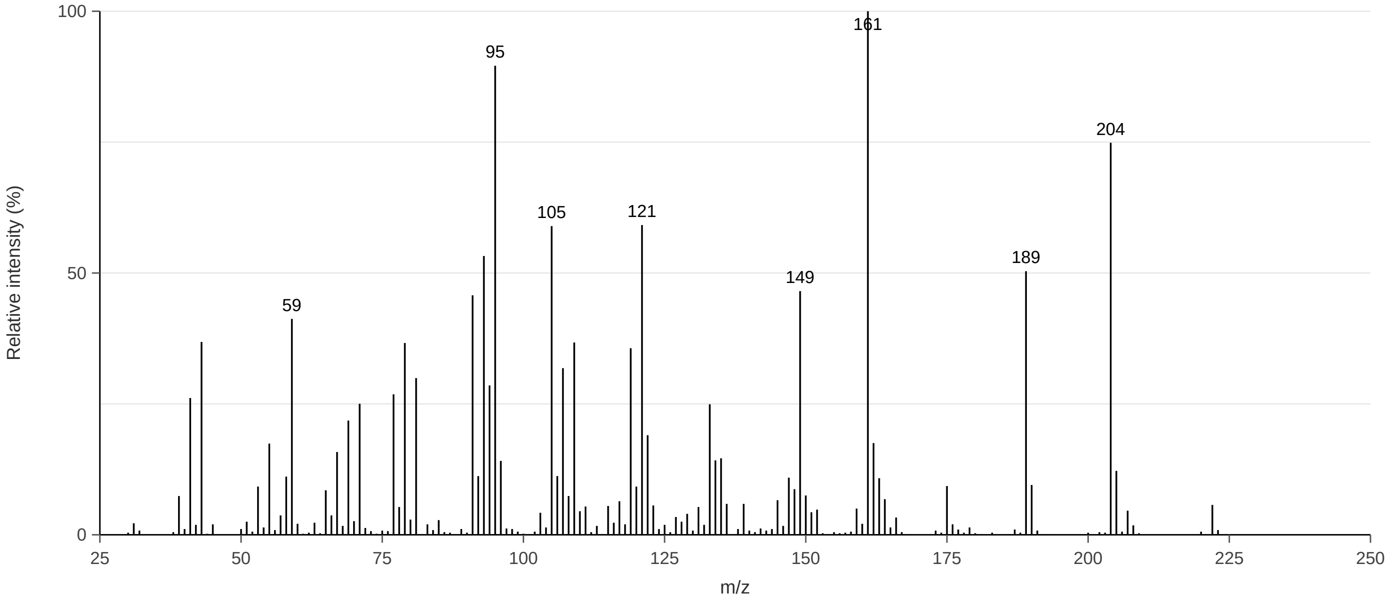

alpha-cadinol

Experimental LRI: 1653

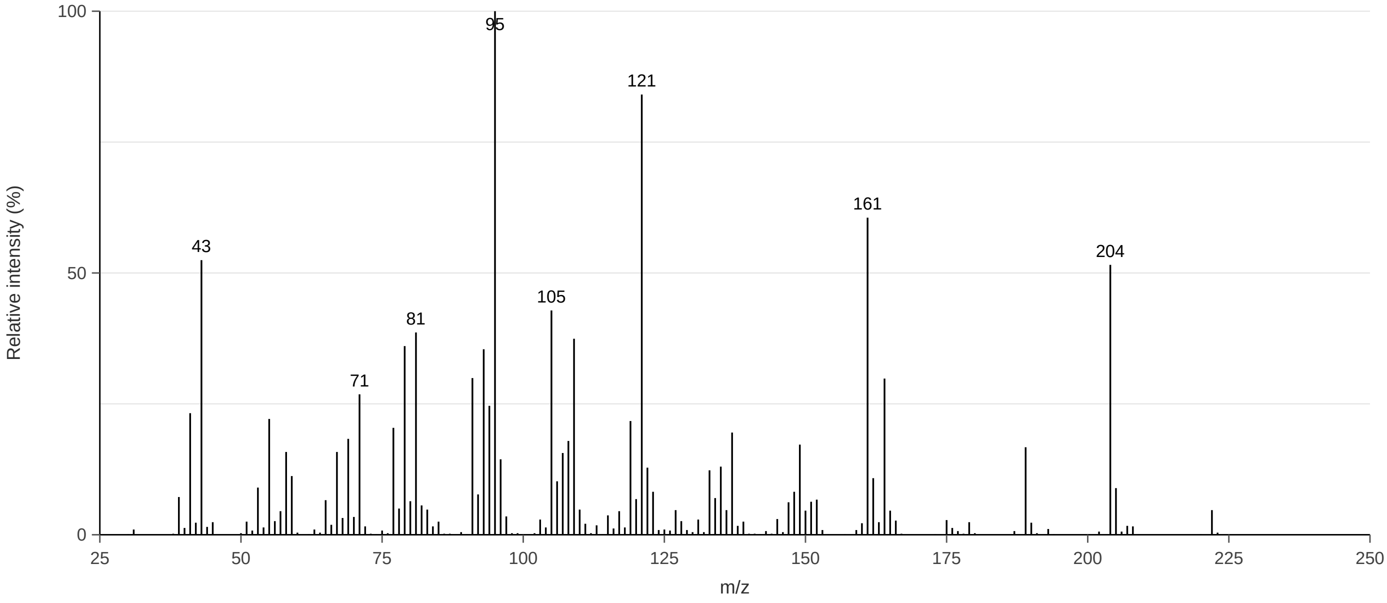

(E,E)-farnesol

Experimental LRI: 1723

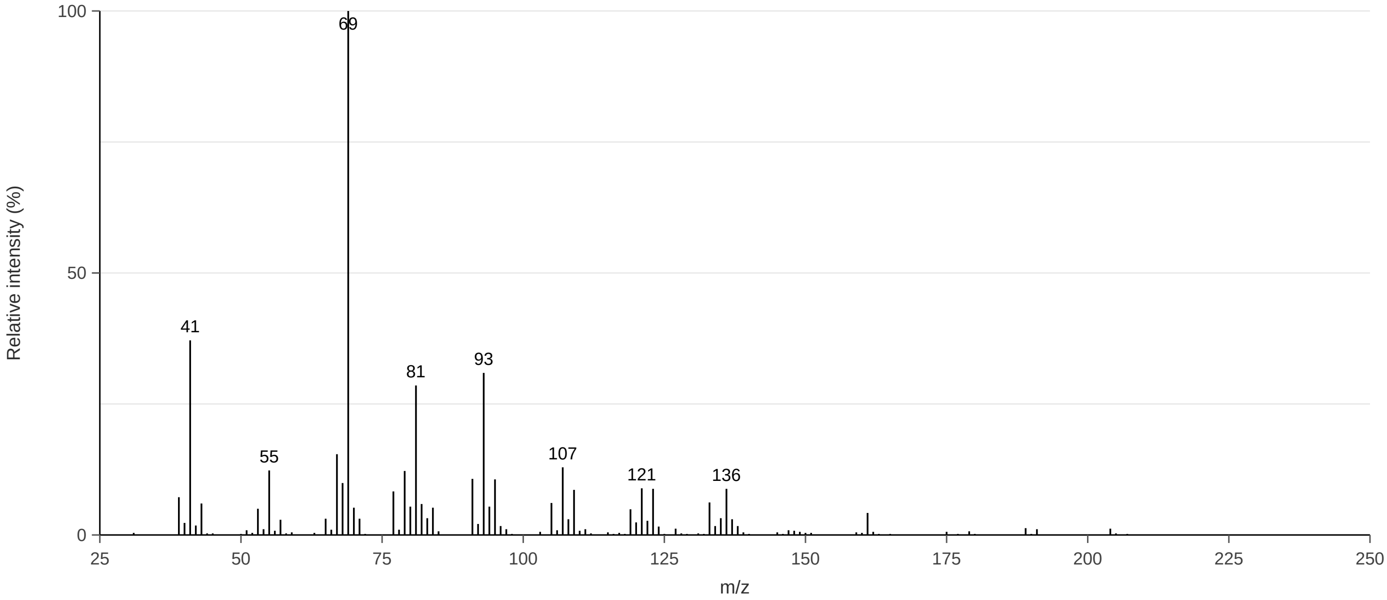

m-camphorene

Experimental LRI: 1960

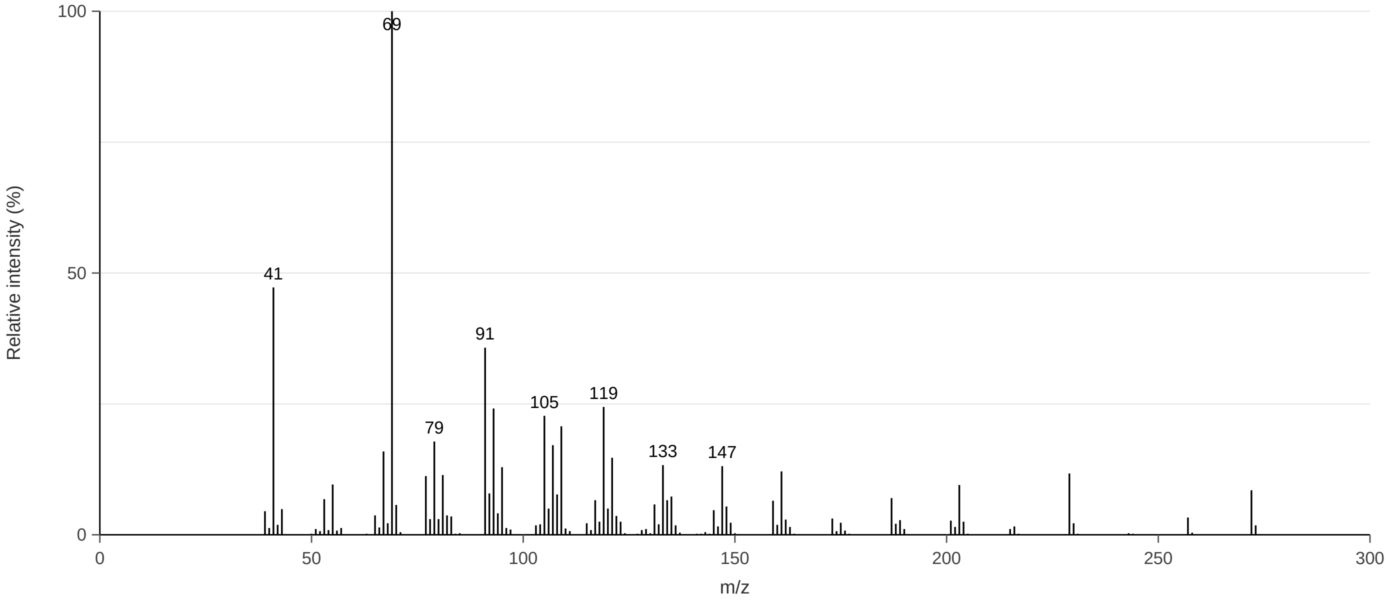

p-camphorene

Experimental LRI: 1995

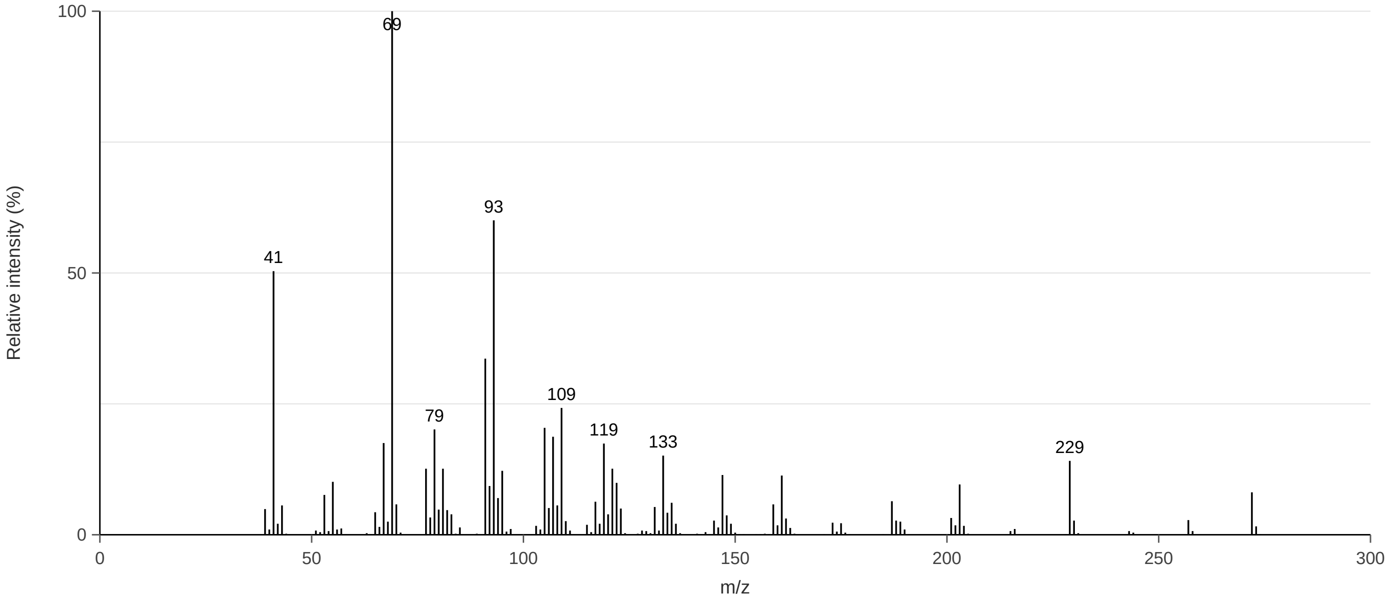

Supplement: Supplementary file 1 [file molecules-31-03487-s001.zip › Supplementary File S1.pdf]
